# New tumor suppressor microRNAs target glypican-3 in human liver cancer

**DOI:** 10.18632/oncotarget.17162

**Published:** 2017-04-17

**Authors:** Flora Cartier, Emilie Indersie, Sarah Lesjean, Justine Charpentier, Katarzyna B. Hooks, Amani Ghousein, Angélique Desplat, Nathalie Dugot-Senant, Véronique Trézéguet, Francis Sagliocco, Martin Hagedorn, Christophe F. Grosset

**Affiliations:** ^1^ University of Bordeaux, Inserm, Groupe de Recherche pour l’Etude du Foie, GREF, U1053, F-33076 Bordeaux, France; ^2^ University of Bordeaux, Inserm, Biothérapies des Maladies Génétiques Inflammatoires et Cancers, BMGIC, U1035, F-33076 Bordeaux, France; ^3^ INSERM US005 - TBM Core, Service for Experimental Histopathology, F-33000 Bordeaux, France; ^4^ University of Bordeaux, F-33000 Bordeaux, France; ^5^ CNRS, UMR5248, Chimie & Biologie des Membranes & des Nano-objets, CBMN, Allée Geoffroy Saint-Hilaire, Bât B14, F-33600 Pessac, France

**Keywords:** liver, cancer, hepatocellular carcinoma, microRNA, glypican-3

## Abstract

Glypican-3 (GPC3) is an oncogene, frequently upregulated in liver malignancies such as hepatocellular carcinoma (HCC) and hepatoblastoma and constitutes a potential molecular target for therapy in liver cancer. Using a functional screening system, we identified 10 new microRNAs controlling *GPC3* expression in malignant liver cells, five of them e.g. miR-4510, miR-203a-3p, miR-548aa, miR-376b-3p and miR-548v reduce *GPC3* expression. These 5 microRNAs were significantly downregulated in tumoral compared to non-tumoral liver and inhibited tumor cell proliferation. Interestingly, miR-4510 inversely correlated with *GPC3* mRNA and protein in HCC samples. This microRNA also induced apoptosis of hepatoma cells and blocked tumor growth *in vivo* in the chick chorioallantoic membrane model. We further show that the tumor suppressive effect of miR-4510 is mediated through direct targeting of *GPC3* mRNA and inactivation of Wnt/β-catenin transcriptional activity and signaling pathway. Moreover, miR-4510 up-regulated the expression of several tumor suppressor genes while reducing the expression of other pro-oncogenes. In summary, we uncovered several new microRNAs targeting the oncogenic functions of GPC3. We provided strong molecular, cellular and *in vivo* evidences for the tumor suppressive activities of miR-4510 bringing to the fore the potential value of this microRNA in HCC therapy.

## INTRODUCTION

MicroRNAs (miRNAs) actively participate in gene regulation in hepatocellular carcinoma (HCC), a primary cancer of the liver affecting adults [1], and in hepatoblastoma (HBL), a rare childhood neoplasm of the liver [2]. HCC is the 5^th^ most common cancer worldwide and the 2^nd^-leading cause of death from cancer, due to its aggressiveness [1]. HCC is a heterogeneous malignancy that usually develops silently on a pre-existing diseased liver with severe fibrosis or cirrhosis [1]. It is therefore often diagnosed at advanced stages resulting in an overall 5-year survival of 20% [3]. Consequently, only one-third of patients can benefit from middle-term curative regimens and Sorafenib is the only validated drug option in patients with more advanced disease [1]. In HBL the introduction of pre-operative chemotherapy and efficient surgical practices have improved disease outcome. However, the prognosis of patients suffering from high-risk tumors is still poor [2]. In this context new effective therapeutic options are urgently needed for the treatment of advanced liver tumors in adults and children.

Glypican-3 (*GPC3*) is one of the numerous oncogenes overexpressed in HCC (Supplementary Figure 1) and HBL [4]. This extracellular co-receptor controls many signaling pathways (e.g. Wnt, Hedgehog, FGF), which are often abnormally activated in liver cancer [1]. For instance, GPC3 interacts with both Frizzled receptor and Wnt ligands and stimulates the canonical Wnt/β-catenin pathway [5, 6]. Moreover the increased amount of cell surface GPC3 induced by the oncogenic human sulfatase 2 (SULF2) protein stimulates Wnt/β-catenin and TGF-β signaling pathways in HCC cells [7–9]. Elevated *GPC3* expression is associated with undifferentiated and proliferative state of cancerous hepatic cells, aggressiveness of HCC tumors, poor prognosis and short overall survival of patients [10]. *GPC3* silencing by small interfering RNAs (siRNAs) or miRNAs inhibits hepatic cancer cell growth [11–13]. Thus *GPC3* clearly constitutes a relevant molecular target in HCC.

MiRNAs are small non-coding RNAs capable of modulating gene expression at the post-transcriptional level [14] by interacting with specific sites, usually located in the 3’ untranslated region (UTR) [11, 15, 16]. As regulators of most cellular functions, miRNAs are intricately involved in human diseases including cancer [17, 18]. Although the effect mediated by some miRNAs on any particular target is modest, the simultaneous regulation of a broad array of target genes by one miRNA can lead to a profound genetic reprogramming and cell-phenotypic changes [17, 18]. Due to their functional redundancy (e.g. multigene targeting) and regulatory properties miRNAs can be used as drugs with promising therapeutic applications in cancer. For instance MRX34, a synthetic encapsulated form of miR-34a-5p, is currently tested for the treatment of primary adult liver cancer [17, 18]. Therefore, miRNA-based therapy might be a solution for the improvement of life expectancy in patients with advanced HCC or high-risk HBL.

Using a functional screening system, the Dual Fluorescence (DF)-FunREG system [11, 19], we uncovered new miRNAs inhibiting *GPC3* expression in HCC cells. Then, we measured expression of these miRNAs in tumoral and non-tumoral liver samples and studied their propensity to act as tumor suppressors *in vitro* using cell-based methods. Finally, the tumor suppressive effect of the most promising *GPC3*-regulating miRNA was further investigated using molecular and functional tools, as well as the chick chorioallantoic membrane model.

## RESULTS

### Selection of fourteen *GPC3*-regulating miRNAs by functional screening

**Figure 1:**
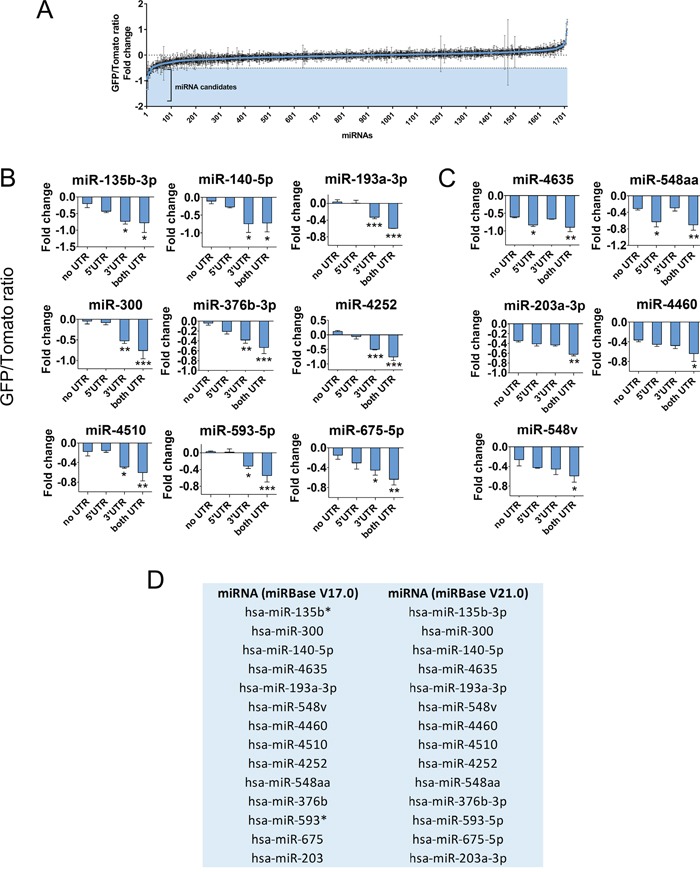
The DF-FunREG screening identifies fourteen potential *GPC3*-regulating miRNAs Huh7 cells co-expressing the Tomato and GFP-5’+3’UTR-GPC3 transgenes were transfected by each miRNA mimic of a library comprising 1712 human miRNAs or by control RNA (Ctrl). MiRNAs decreasing the GFP/Tomato ratio compared to Ctrl were selected as candidates. **(A)** First step of DF-FunREG screening. The graph shows GFP/Tomato ratio fold change variations for the 1712 tested miRNAs. Dots represent means +/- standard deviation (SD) (n=3). **(B-C)** Second step of DF-FunREG screening. Fourteen miRNAs down-regulate the GFP/Tomato ratio fold change through *GPC3* 3’-UTR **(B)**, 5’-UTR or both **(C)**. Bars represent means + SD (n=3, ANOVA p<0.01). **(D)** The fourteen miRNAs retained following the DF-FunREG screening are shown with their names in the V17.0 and V21.0 versions of miRBase. In this Figure and the following, the ANOVA test was followed by a multiple comparison post-test (see Statistical analyses section for details), *: p < 0.05, **: p < 0.01, ***: p < 0.001.

To identify novel miRNAs negatively regulating *GPC3* through its two UTRs, we performed a new *in vitro* functional screening of a library of 1712 individual miRNAs using the HCC-derived Huh7 cell line and an updated version of the DF-FunREG system [11]. Twenty out of 1712 miRNAs were retained according to a decrease of -0.5 or more of GFP/Tomato ratio fold change (Figure 1A and Supplementary Table 1). Such threshold is indicative of a significant decrease resulting from the specific action of these miRNA on the UTRs of GPC3 bordering a gene reporter (GFP) in comparison to a reference gene (Tomato). Three out of these 20 miRNAs e.g. miR-96-5p, miR-1271-5p and miR-1973 were excluded from further analyses because they were subject of early investigations by our team [11, 13]. The remaining 17 candidate miRNAs were transfected into Tomato-positive Huh7 cells co-expressing the “GFP” transgene lacking the *GPC3* UTRs to eliminate false positive miRNAs or either the “GFP-5’UTR-GPC3” or the “GFP-GPC3-3’UTR” transgene to determine their UTR preference. These control experiments allowed us to exclude three false positive miRNAs (Supplementary Figure 2) and to keep only 14 miRNAs (Figure 1B-1D). As expected, most of them act through the 3’-UTR (Figure 1B) but some exert a moderate effect either through *GPC3* 5’-UTR [16] or both UTRs [20] (Figure 1C). Contrarily to miRNA paralogs miR-96-5p and miR-1271-5p [11, 13], the previously described *GPC3*-regulating miRNAs, miR-219-5p and miR-520c-3p [21, 22] were below the cut-off (Supplementary Table 1) and thus not selected in our screening. The final outcome of selection by the DF-FunREG screening is a total of 14 new and potent *GPC3*-regulator miRNAs (Figure 1D).

### Ten miRNAs regulate GPC3 expression in HCC cells

**Figure 2:**
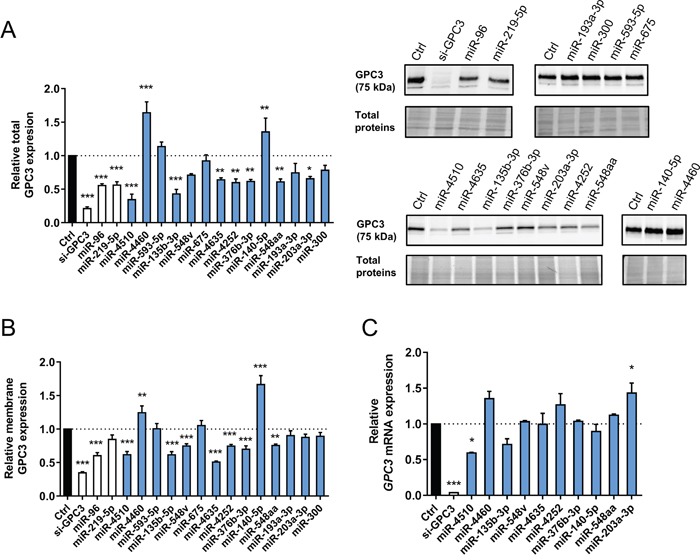
Ten miRNAs regulate GPC3 expression **(A)** The relative expression of total GPC3 protein was measured by western blot in Huh7 cells following transfection with small RNAs (left panel). The amount of GPC3 protein was normalized to the total amount of loaded proteins (see Materials and Methods section). Bars represent means + standard error of the mean (SEM) (n=5, ANOVA p<0.0001). The negative control is shown as a black bar. The si-*GPC3* and previously reported *GPC3*-regulating miRNAs are shown as white bars and the fourteen retained miRNAs as blue bars. Representative western blots of 5 independent experiments are shown on the right panel. The top blots show results obtained with control RNAs (left) and ineffective miRNAs (right). The bottom blots show miRNAs inhibiting (left) or increasing (right) the amount of GPC3. Protein size is shown in brackets on the left of the blot. All cropped blots retained at least 6 bandwidths above and below the bands. SYPRO Ruby-labeled proteins cropped blots correspond to the middle part of the labeled membrane. **(B)** The relative expression of membrane-anchored GPC3 protein was measured by FACS in Huh7 cells transfected with the indicated small RNAs using the anti-human GPC3-Allophycocianin (APC) monoclonal antibody. Results are shown as Mean Fluorescence Intensity ratios. Bars represent means + SEM (n=4, ANOVA p<0.0001). See panel A for legend of colored bars. **(C)** The relative expression of *GPC3* mRNA was measured by real-time quantitative RT-PCR in Huh7 cells following small RNA transfection. Bars represent means + SEM (n=3, ANOVA p<0.0001). See panel A for legend of colored bars. *: p < 0.05, **: p < 0.01, ***: p < 0.001.

The next step was to evaluate the regulatory effect of the 14 selected miRNAs on endogenous GPC3 protein expression in Huh7 cells. A siRNA against *GPC3* and the miRNAs miR-96-5p and miR-219-5p were used as positive controls [11, 13, 22]. Among the 14 miRNAs, nine were able to control endogenous GPC3 protein expression (seven negatively and two positively, Figure 2A). Since GPC3 is anchored at the external side of the membrane, we also measured its levels at the membrane after miRNA transfection. The regulatory effect of miR-4510, miR-4460, miR-135b-3p, miR-4635, miR-4252, miR-376b-3p, miR-140-5p and miR-548aa on membrane GPC3 levels was confirmed (Figure 2B). However, miR-203a-3p and the control miR-219-5p had no significant inhibitory effect (Figure 2B). Moreover, miR-548v specifically decreased GPC3 membrane levels (Figure 2B) confirming the trend observed with the endogenous protein (Figure 2A). Together, these investigations validated ten miRNAs regulating GPC3 expression in Huh7 cells. Finally, we assessed the effect of these ten miRNAs on *GPC3* mRNA expression. MiR-4510, which exerted the strongest inhibitory effect on total GPC3 protein (Figure 2A-2B), also decreased *GPC3* mRNA expression (Figure 2C). Altogether, we noticed few differences between the screening results (Figure 1) and GPC3 expression following miRNA transfection (Figure 2). Those differences may be due to the pleiotropic effects of miRNAs on multiple targets or the involvement of other post-transcriptional regulators [11, 14, 16]. To summarize, our investigations led to the validation of ten miRNAs regulating *GPC3* protein synthesis in HCC cells: miR-4510, miR-4460, miR-135b-3p, miR-548v, miR-4635, miR-4252, miR-376b-3p, miR-140-5p, miR-548aa and miR-203a-3p. *In silico* predictions indicate that some of them (e.g. miR-4510) may function through a direct miRNA:*GPC3* mRNA interaction (Supplementary Table 2).

### Downregulation of five *GPC3*-regulating miRNAs in HCC

**Figure 3:**
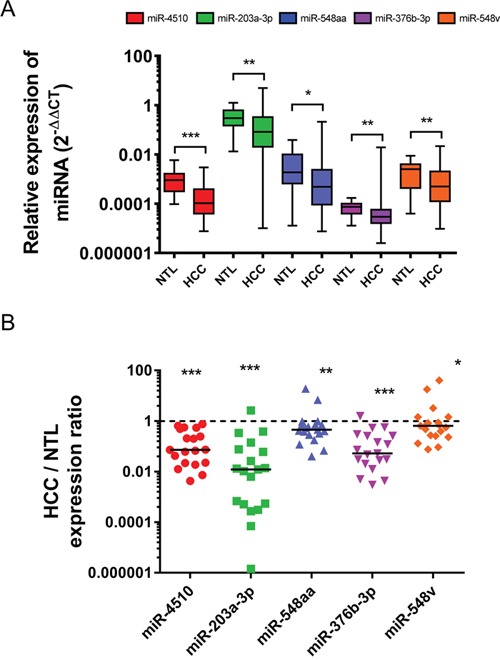
Five *GPC3*-regulating miRNAs are downregulated in HCC **(A)** The relative expression of miR-4510, miR-203a-3p, miR-548aa, miR-376b-3p and miR-548v was measured by real-time quantitative RT-PCR in 19 NTL and 98 HCC. Data are presented as box and whiskers with minimal and maximal values (two-tailed unpaired *t* test). **(B)** The relative expression of miR-4510, miR-203a-3p, miR-548aa, miR-376b-3p and miR-548v was measured by real-time quantitative RT-PCR in 19 pairs of HCC and adjacent NTL. Results are presented as HCC/NTL expression ratios. The median is shown as a full line and the reference ratio value “1” is shown as a dotted line. The statistical analyses were done with the two-tailed Wilcoxon matched-pairs signed ranked test. *: p < 0.05, **: p < 0.01, ***: p < 0.001.

The expression of the 10 novel *GPC3*-regulating miRNAs was measured by RT-qPCR in a first cohort of 19 non-tumorous livers (NTL) and 98 HCC samples. In parallel, expression of these miRNAs was compared in a second cohort of 19 pairs of liver tumor/adjacent NTL from patients with HCC. MiR-4460 was not detected in liver, whereas miR-135b-3p, miR-4635, miR-4252 and miR-140-5p were not deregulated in tumors compared to NTL (Supplementary Figure 3A-3B). Instead, miR-4510, miR-203a-3p, miR-548aa, miR-376b-3p and miR-548v were significantly decreased in HCC (Figure 3A-3B). The expression of each miRNA was correlated to HCC patients’ clinical parameters. While miR-548aa downregulation in HCC was associated with the presence of satellite nodules, miR-376b-3p downregulation was associated with tumors harboring p53 mutations (Supplementary Table 3). Altogether, our data showed that the five *GPC3*-regulating miRNAs miR-4510, miR-203a-3p, miR-548aa, miR-376b-3p and miR-548v are significantly downregulated in HCC compared to NTL.

### The five HCC down-regulated miRNAs exert antitumor effects

**Figure 4:**
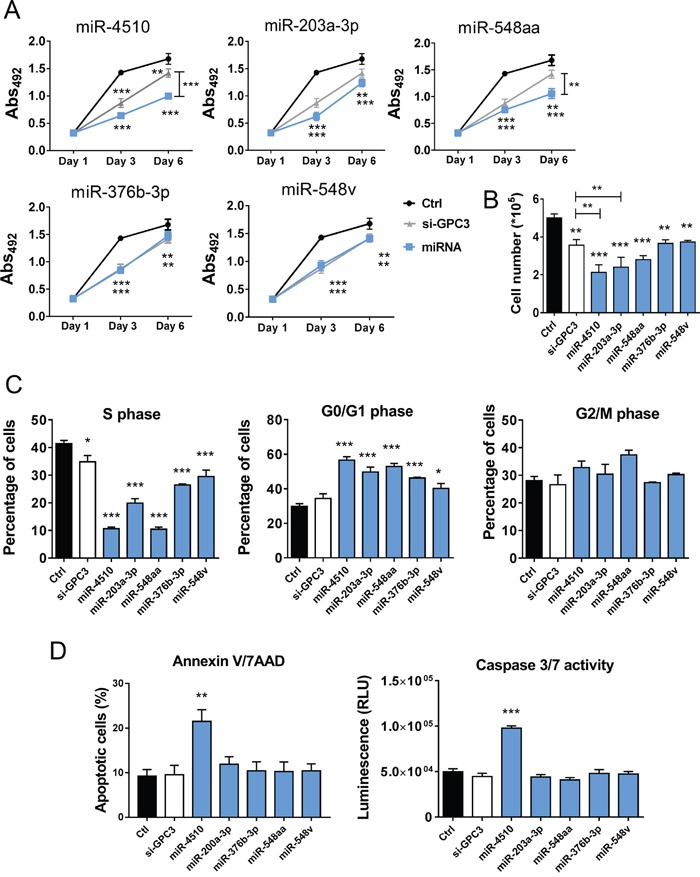
MiR-4510, miR-203a-3p, miR-548aa, miR-376b-3p and miR-548v exert an antitumor effect on HCC cells **(A-D)** Huh7 cells were transfected with miRNAs (**A**, blue lines + symbols; **B-D**, blue bars), si-GPC3 (**A**, grey lines + symbols; **B-D**, white bars) or controls (Ctrl RNA; **A**, black lines + symbols; **B-D**, black bars) and different cell-based assays were performed. **(A)** Cell growth was measured at the indicated time points using the Sulforhodamine B colorimetric assay (Abs 492). Results are presented as mean +/- SEM (n=5, ANOVA p<0.0001). **(B-C)** Three days after transfection, cell proliferation was determined by cell counting **(B)** and cell cycle was measured by APC/BrdU staining (percentages of cells in S, G0/G1 and G2/M phases, **C**). **(D)** In parallel, the percentage of apoptotic cells was determined by annexin/7-ADD staining (left panel) and caspase 3/7 activity was measured by a luminescent assay (right panel). **(B-D)** Bars represent means + SEM (n=3, ANOVA p<0.01). *: p < 0.05, **: p < 0.01, ***: p < 0.001.

We next investigated the effect of the five down-regulated miRNAs on tumor cells *in vitro*. All miRNAs significantly inhibited Huh7 cell growth and proliferation (Figure 4A-4B). However, compared to miR-4510 alone, different combinations of the miRNAs did not further potentiate growth inhibition (data not shown). The inhibitory effect of each miRNA on Huh7 cell proliferation could be abolished by the simultaneous use of corresponding anti-miRs (Supplementary Figure 4). The specific blocking of four out of five endogenous miRNAs led to a slight increase in Huh7 cell proliferation (Supplementary Figure 4). We further studied the effect of these five miRNAs on different phases of cell division. All five miRNAs increased the percentage of cells in G0/G1 phase and reduced the percentage of cells in S phase (Figure 4C). Finally, we investigated the ability of these miRNAs to induce apoptosis using two complementary assays. Annexin V/7AAD staining and caspase 3/7 activity revealed that only miR-4510 induced Huh7 apoptosis (Figure 4D and Supplementary Figure 5). Altogether these results demonstrate that miR-4510, miR-203a-3p, miR-548aa, miR-376b-3p and miR-548v are potent inhibitors of HCC cell proliferation.

### MiR-4510 is a powerful antitumoral agent in liver cancer and acts through *GPC3* 3’-UTR targeting

**Figure 5:**
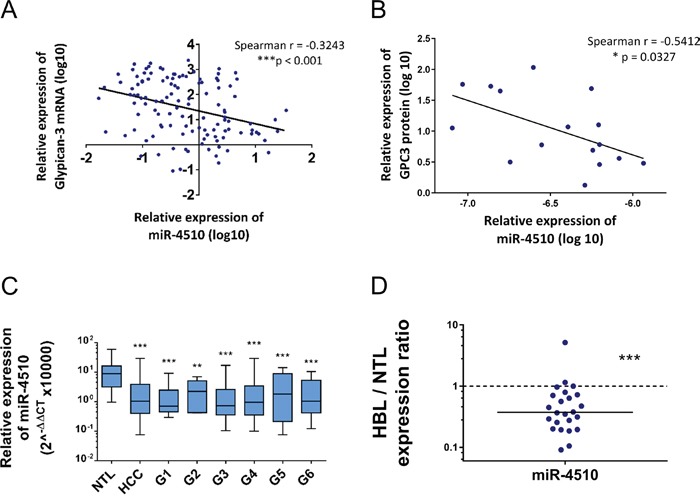
Expression of miR-4510 in HCC and HBL and correlation with *GPC3* mRNA expression **(A)** Inverse correlation between *GPC3* mRNA and miR-4510 expressions measured by real-time quantitative RT-PCR in 98 HCC. Spearman r correlation = -0.3243, ***p<0.001. **(B)** Inverse correlation between *GPC3* protein level and miR-4510 expression measured in 16 HCC samples by immunoblotting and real-time quantitative RT-PCR, respectively. Spearman correlation, r = -0.5412, *p<0.05. **(C)** Relative expression of miR-4510 in HCC subgroups. Data are presented as box and whiskers plots with minimal and maximal values (ANOVA p<0.0001). Dunnett's multiple comparisons test. **(D)** Relative expression of miR-4510 in 24 pairs of HBL and adjacent normal liver samples. Results are presented as HBL/NTL expression ratios. The median is shown as a full line and the reference ratio value “1” is shown as a dotted line. Two-tailed Wilcoxon matched-pairs signed ranked test. **: p < 0.01, ***: p < 0.001.

**Figure 6:**
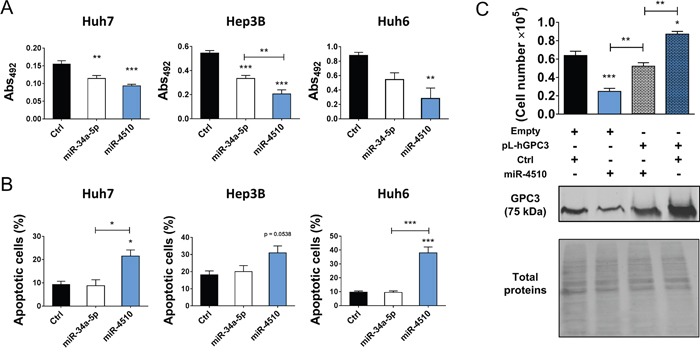
MiR-4510 acts as a tumor suppressor *in vitro* **(A)** Six days after transfection, the effect of miR-4510 and miR-34a-5p on the growth of Huh7, Hep3B and Huh6 cells was compared using the Sulforhodamine B colorimetric assay (Abs 492nm). Bars represent means + SEM (n=4, ANOVA p<0.01). **(B)** Three days after transfection, the effects of miR-4510 and miR-34a-5p on the apoptosis of Huh7, Hep3B and Huh6 cells were compared by annexin/7-ADD staining. Bars represent means + SEM (n=3, ANOVA p<0.05). **(C)** Huh7 cells were transduced with lentiviruses containing the *GPC3* transgene lacking the 5’- and 3’UTR (pL-hGPC3) or an empty transgene (Empty). Three days later, cells were transfected with miR-4510 or Ctrl. Finally, cell number was measured by cell counting and the amount of GPC3 protein was assessed by western blotting three days later. Top panel: bars represent means + SEM (n=3, ANOVA p<0.0001). Bottom panel: one representative immunoblot of 3 independent experiments is shown. *: p < 0.05, **: p < 0.01, ***: p < 0.001.

**Figure 7:**
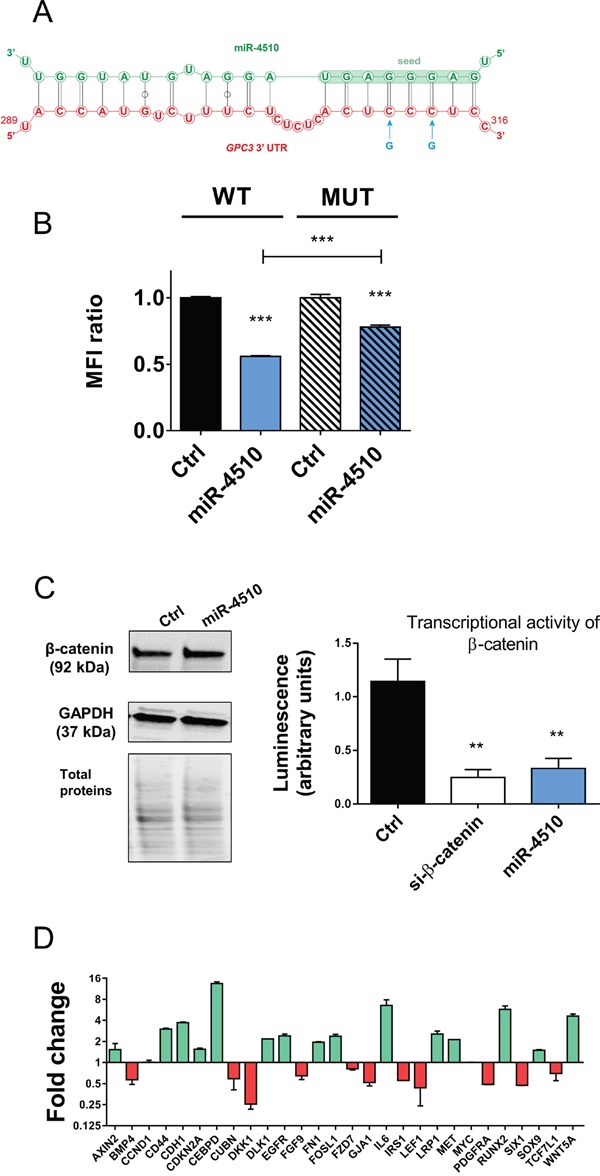
miR-4510 directly binds *GPC3* 3’UTR and inhibits Wnt/β-catenin signaling pathway **(A)** Schematic representation of miR-4510:*GPC3* mRNA interactions predicted by RNAhybrid. Through its seed sequence, miR-4510 (green sequence) interacts with *GPC3* 3’-UTR (red sequence) at position 308-315. Blue arrows correspond to the 2 mutated nucleotides (r.311C>G and r.313C>G). **(B)** Huh7 cells expressing the GFP and wild type *GPC3*-3’UTR transgene (WT cells) or the mutated *GPC3-*3’UTR transgene (Mut cells) were transfected with miR-4510 or Ctrl. The Mean Fluorescence Intensity (MFI) ratio was measured in each cell population using the FunREG method. Bars represent means + SEM (n=3, ANOVA p<0.0001). **(C)** Effect of miR-4510 on Wnt/β-catenin pathway. The expression of β-catenin and GAPDH was assessed by Western blotting in Huh7 cells 72hr after transfection (left panel; one representative blot of 3 independent experiments is shown). Protein size is shown in brackets on the left of the blot. All cropped blots retained at least 6 bandwidths above and below the bands. SYPRO Ruby-labeled proteins cropped blots correspond to the middle part of the labeled membrane. The transcriptional activity of β-catenin was measured by TOPflash/FOPflash assay 72hr after transfection with siRNA targeting β-catenin as positive control or miR-4510 (right panel). Bars represent means + SEM (n=3, ANOVA p<0.0001). **(D)** miR-4510 induces the up- or down-regulation of Wnt/β-catenin pathway-associated genes mRNA. Data are presented as Log_2_ fold-change ratio between miR-4510- and Ctrl-transfected Huh7 cells. Bars represent means + or - SEM (n=2). **: p < 0.01, ***: p < 0.001.

We next investigated in more details expression of miR-4510 and *GPC3* mRNA in liver tumor samples. We noticed that miR-4510 was constantly decreased in HCC (Figure 3B) and inversely correlated with *GPC3* mRNA (Figure 5A) and protein (Figure 5B) in HCC samples, making a strong and direct connection between the reduction of this miRNA and *GPC3* overexpression. No inverse correlation was observed when we compared expression of other miRNAs (miR-203a-3p, miR-548aa, miR-376b-3p and miR-548v) and the amount of *GPC3* mRNA or protein in patient samples (Supplementary Figure 6A-6B). MiR-4510 decrease was independent of HCC subgroup clustering (Figure 5C) [23] and was also observed in HBL tumors (Figure 5D). Thus a decrease of this miRNA constitutes a good indicator of liver tumorigenesis. Because miR-4510 efficiently impaired cell proliferation and induced apoptosis of Huh7 cells (Figure 4), we compared its effects on hepatoma cells with that of miR-34a-5p, a miRNA currently tested in clinic (MRX34) [1, 18, 24]. MiR-4510 inhibited the growth of HCC-derived Huh7 and Hep3B cells and of HBL-derived Huh6 cells (which carry a missense G34V mutation in *β-catenin/CTNNB1* gene) and it was significantly more effective than miR-34a-5p in Hep3B cells (Figure 6A). Moreover, it induced apoptosis in the three hepatoma cell lines tested while miR-34a-5p had no pro-apoptotic effect (Figure 6B). Finally, the proliferative capacity of miR-4510-transfected Huh7 cells was partly rescued by the ectopic expression of a *GPC3* transgene devoid of its 5’- and 3’-UTRs (pL-hGPC3, Figure 6C). Finally, according to the screening data (Figure 1B) and *in silico* predictions, a target site of miR-4510 was located in *GPC3* 3’-UTR at position 308-315 (Figure 7A). To assess the relevance of this site, two point mutations (r.311C>G and r.313C>G) were inserted in this 3’-UTR sequence. Then FunREG analysis was performed [25, 26]. As expected, miR-4510 decreased the fluorescence of GFP with a wild-type *GPC3* 3’-UTR (Figure 7B) demonstrating its ability to regulate *GPC3* at a post-transcriptional level. Interestingly, this decrease was partly abrogated when the 3’UTR contained the mutated sequence (Figure 7B) showing that miR-4510 physically interacts with *GPC3* 3’-UTR at this position. Altogether these data demonstrate that miR-4510 is a powerful antitumoral agent in liver cancer and acts in part through *GPC3* downregulation.

### MiR-4510 inhibits Wnt/β-catenin signaling pathway

Because miR-4510 inhibits liver cancer cell proliferation through GPC3, which is involved in Wnt/β-catenin pathway activation [5, 6], we investigated miR-4510 effect on this pathway. MiR-4510 inhibited the transcriptional activity of β-catenin in Huh7 and Huh6 cells without affecting β-catenin expression (Figure 7C and Supplementary Figure 7A) or its subcellular localization (Supplementary Figure 8). The inhibition of Wnt pathway by miR-4510 in Huh7 cells was accompanied by the decrease of associated genes including two transcriptional cofactors of β-catenin, such as *LEF1* and *TCF7L1* [27, 28], direct targets of Wnt/β-catenin signaling (*IRS1*, *BMP4*, *FGF9* and *LEF1*), and various genes associated with poor-prognosis HCC such as *BMP4*, *PDGFRA* and *SIX1* (Figure 7D). On the other hand, miR-4510 treatment led to an increase in several important regulators of β-catenin functions and tumor suppressors such as *AXIN2*, *E-cadherin* (*CDH1*), *LRP1* and *WNT5A* (Figure 7D). The expression of several pro-oncogenic genes in liver (*GJA1*, *IRS1*, *PDGFRA* and *SIX1*) was also decreased in miR-4510-transfected Huh7 cells (Figure 7D). Surprisingly, we did not observe any effect of miR-4510 on *MYC* and *Cyclin D1* mRNA in Huh7 but this might be due to the wild-type status of β-catenin or the possible influence of other signaling pathways such as p53 in these cells [1, 28]. In contrast, miR-4510 decreased the amount of Cyclin D1 protein in Huh6 cells (Supplementary Figure 7B). Altogether these data showed that miR-4510 inhibits Wnt/β-catenin pathway.

### MiR-4510 inhibits tumor growth and induces apoptosis of HCC cells *in vivo*

**Figure 8:**
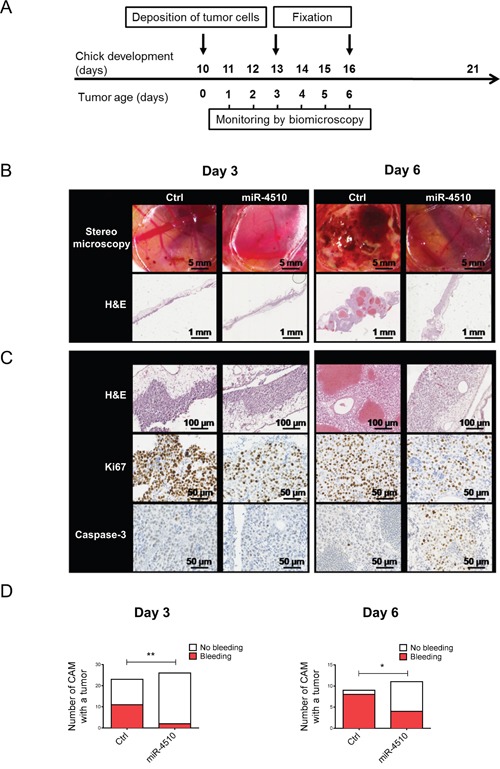
MiR-4510 inhibits HCC tumor development *in vivo* **(A)** Twenty-four hours after transfection with miR-4510 or Ctrl miRNA, Huh7 cells were collected and grafted on the chicken CAM. Tumor growth was monitored from day 1 to day 6. Tissue fixation was done at day 3 and day 6. **(B)** Photographs of tumors (top panels) and hematoxylin and eosin (H&E) staining (bottom panels) were performed 3 and 6 days after cells implantation. **(C)** Hematoxylin and eosin (H&E), Ki67 and cleaved Caspase-3-staining was performed on sections of tumors treated with miR-4510 or Ctrl. Magnification scale bars are as indicated on each microscopic image. **(D)** The number of CAMs with tumor presenting or not bleeding in Ctrl *versus* miR-4510 at Day 3 (left panel) and Day 6 (right panel) is shown as bars. Two-sided Fisher's exact test, *: p < 0.05, **: p < 0.01.

We next evaluated the antitumor activity of miR-4510 *in vivo* using the CAM model. Critical biological features of human tumor progression such as cell proliferation, angiogenesis, normal tissue invasion, and tumor cell-host interactions have been previously successfully reproduced in this model [29, 30]. Moreover, the CAM model is useful for testing small non-coding RNA-mediated gene knockdown on tumor growth and angiogenesis [31]. We transfected Huh7 cells with miR-4510 or Ctrl and validated the inhibition of GPC3 by miR-4510 (Supplementary Figure 9A-9B). We deposited these Huh7 cells on the CAM and monitored tumor growth on days 3 and 6 (Figure 8A). At day 3 no obvious macroscopic differences were observed between Ctrl and miR-4510 in tumor appearance or size (Figure 8B, upper panels). Staining of tissue cross-sections with Hematoxylin and Eosin did not reveal any difference either (Figure 8B-8C, H&E panels). However, there was a significantly lower number of tumors with bleeding in presence of miR-4510 indicating a reduction of Huh7 cells aggressiveness (Figure 8D, left panel). GPC3 protein expression was also decreased in tumors transfected with miR-4510 compared to control (Supplementary Figure 9C). As expected, miR-4510 levels were increased while *GPC3* mRNA was unchanged (Supplementary Figure 9D-9E). At day 6, miR-4510 levels remained high and *GPC3* mRNA expression significantly decreased (Supplementary Figure 9D-9E). At this stage, the growth of miR-4510 tumors was noticeably impeded compared to control tumors (Figure 8B-8C, upper panels), as assessed by a disappearance of bloody and coagulation areas (Figure 8B, upper panels) and large vessels in tumoral tissue (Figure 8B, lower panels and 8C, upper panels). As shown in Figure 8D, right panel, 80% of Ctrl tumors and only 30 % of miR-4510 tumors were characterized by bleeding at Day 6, further indicating a reduction of the aggressiveness of miR-4510-transfected HCC cells during tumor development. Next, we characterized the effects of miR-4510 on tumor cell proliferation and apoptosis, respectively by staining of Ki67 and cleaved Caspase 3. The decrease of the proliferative marker Ki67 in miR-4510 transfected-tumors was obvious both at day 3 and day 6 of tumor growth (Figure 8C, middle panels), indicating that miR-4510 was capable of inhibiting the proliferation of HCC. As for apoptosis analysis, cleaved Caspase-3 staining was not detectable at day 3 and 6 in control tumors. However, tumors transfected with miR-4510 were highly positive for cleaved Caspase-3 at day 6 suggesting that miR-4510 induces HCC cell apoptosis at later stages of tumor development (Figure 8C, lower panels). Altogether these results showed that miR-4510 induces apoptosis and inhibits the growth and angiogenesis of HCC tumors *in vivo*.

## DISCUSSION

In this work we identified new miRNAs regulating *GPC3*. Among them, 5 (namely miR-203a-3p miR-4510, miR-548aa, miR-376b-3p and miR-548v) inhibited GPC3 expression in HCC cells and acted as tumor suppressors in liver by inhibiting HCC cell growth and proliferation. So far, only miR-203a-3p has been described as a mediator of liver carcinogenesis [32]. Therefore, miR-4510, miR-548aa, miR-376b-3p and miR-548v are new actors in liver cancer. Remarkably, these 5 miRNAs were significantly decreased in HCC tumors (Figure 3). These results suggest that the upregulation of *GPC3* in liver cancer originates in part from the simultaneous downregulation of multiple specific miRNAs as already described for other cancers [24, 33, 34]. Thus this might be a more general mechanism developed by cancer cells to maintain oncogenicity as discussed previously [33, 35, 36]. Other mechanisms responsible for the oncogenic overexpression of *GPC3* in HCC have been shown before, including transcriptional activation by Myc [37] and increase in *GPC3* copy number [38]. Therefore, miRNA downregulation might be one of the molecular processes set up by HCC cells to maintain high level of GPC3 and activate oncogenic downstream signaling including the Wnt pathway [1].

Several microRNAs negatively regulating *GPC3* have been described in liver cancer cells [11, 13, 16, 21, 22]. Surprisingly, while we confirmed the inhibition of *GPC3* by miR-219-5p (Figure 2A), this miRNA and miR-520c-3p [21, 22] were not retained in our screen using *GPC3* 5’- and 3’-UTRs as baits (Supplementary Table 1). These data suggest that those two miRNAs may target *GPC3* mRNA indirectly or through its coding region. Only miR-1271-5p and miR-219-5p are decreased in HCC samples [11, 22] and we previously reported that *GPC3* mRNA inversely correlates with miR-1271-5p in HBV-positive HCC subgroup [11]. Here, we report the decrease of 5 new *GPC3*-regulating miRNAs in two independent cohorts of liver cancer tissues. While we cannot exclude the possibility that different miRNAs (including miR-219-5p and miR-1271-5p) play a role in HCC-associated *GPC3* overexpression, our data suggest that miR-4510 is of particular importance in this cancer as it strongly and inversely correlated with *GPC3* mRNA and protein levels in HCC tumors (Figure 5A-5B). MiR-4510 may also play a tumor suppressive role in colorectal adenocarcinoma in which it is decreased [39]. Therefore, we speculate that miR-4510 plays a major role in the deregulation of *GPC3* in liver cancer.

Among the 10 new miRNAs identified, miR-4510 was the most potent inhibitor of *GPC3* expression. It induced proliferation arrest and apoptosis in all tested cell lines more efficiently than miR-34a-5p, the first miRNA-mimic tested in clinic. Our data show that the tumor suppressor effect of miR-4510 is related to the reduction of GPC3 but also to Wnt signaling inactivation. Importantly, the inhibition of Wnt pathway mediated by miR-4510 was independent of β-catenin expression and localization. Rather, miR-4510 inactivates Wnt/β-catenin signaling not only at its initiation step through GPC3 but also at a later stage by silencing LEF1 and TCF7L1 (Figure 7D) [28]. Moreover, it decreases expression of several oncogenic genes under the control of Wnt/β-catenin pathway including *GJA1*, *IRS1*, *PDGFRA* and *SIX1* [40–42]. We speculate that miR-4510-transfected cells reactivate the inhibited Wnt pathway by upregulating the pro-inflammatory factor IL6 and several Wnt cofactors such as CDH1/E-cadherin, LRP1 and WNT5A (Figure 7D). Thus, miR-4510 directly and indirectly inhibits several oncogenes in liver tissues and might constitute an important tumor suppressor in liver that antagonizes key tumoral processes (e.g. uncontrolled proliferation and cell survival) and inactivates signaling cascades (e.g. Wnt pathway). Further experiments aiming at identifying the other targets of miR-4510 in HCC cells should shed some light on its precise role in liver carcinogenesis.

There is a growing therapeutic interest in the use of miRNAs in clinical oncology because of their small size, easy manufacturing and pleiotropic effect [1, 18, 43]. MiR-34a-5p, which constitutes a miRNA mimic encapsulated in ionizable liposomes (MRX34), is being tested since spring 2013 in a multicentric clinical trial [24]. Another miRNA, miR-16, is in clinical trials since early 2015 for mesothelioma and lung cancer [43]. Given the progress of siRNA- and miRNA-replacement therapy in cancer [1, 18, 43], our data provides strong molecular, cellular and *in vivo* evidences supportive of miR-4510-based drugs as an option for the treatment of patients with HCC or HBL. The tumor growth inhibition mediated by miR-4510 *in vivo* further supports its relevance as a candidate for miRNA-replacement therapy for the treatment of patients with advanced liver cancer.

In conclusion, our report further demonstrates the active role of miRNA deregulations in oncogenic processes and brings new information about the complex miRNA:*GPC3* relationships occurring in liver tumors. We therefore expect than the data presented in this report will favor the development of new therapeutic solutions for the treatment of patient with HCC or HBL.

## MATERIALS AND METHODS

### Plasmids construction

The lentiviral plasmids pTRIP-0, pL-GFP, pL-Tomato and pL-GFP-GPC3 (bearing the *GPC3* 3’-UTR and renamed pL-GFP-GPC3-3’UTR) have been previously described [11, 13, 26]. The pL-GFP-5’+3’UTR-GPC3 and pL-GFP-5’UTR-GPC3 constructs were obtained by inserting the *GPC3* 5’-UTR in the pL-GFP-GPC3-3’UTR and pL-GFP plasmids, respectively. The pL-GFP-GPC3-3’UTR-Mut plasmid was constructed as follows: the mutated *GPC3* 3’-UTR sequence bearing the two-point mutations r.311C>G and r.313C>G was obtained by gene synthesis (MWG Biotech) and cloned in the pEX-A2 plasmid. Then the insert was subcloned into the XhoI-KpnI-digested pL-GFP-GPC3-3’UTR. To construct pGEM-T-hGPC3 plasmid, the *GPC3* Open Reading Frame (ORF) was amplified by PCR using the primers 5’-ATTCTCTAGAGAATTCGGATCCATGGCCGGGACCGTGCGC-3’ and 5’-CTCACTCTAGAGCGGCCGCTCAGTGCACCAGGAAG-3’ and the pEF-BOS plasmid containing the human *GPC3* cDNA as template, which was kindly provided by Jorge Filmus [44]. After adenylation, the PCR fragment was subcloned into the pGEM®-T vector (Promega). The sequences in all constructions were verified by DNA sequencing. The lentiviral pL-hGPC3 was constructed by subcloning the human *GPC3* ORF from the pGEM-T-hGPC3 plasmid into the BamHI-XbaI-digested pL-GFP plasmid.

### Cell lines

The HCC-derived Huh7 and Hep3B cell lines were grown as described before [11, 13, 26]. The HBL-derived Huh6 cells were grown in DMEM 1g/L (Invitrogen, Carlsbad, California, USA) containing 10% fetal bovine serum (FBS) and penicillin/streptomycin (1000 units/mL). Cell lines were yearly identified using STR profiling (ATCC-LGC Standard) and regularly tested for mycoplasma-free infection. Stable Huh7 cell lines co-expressing Tomato and GFP transgenes (with the *GPC3* 5’-UTR, 3’-UTR, both or neither) were developed by lentiviral transduction using a multiplicity of infection of 1 and cell sorting. Production and titration of infectious lentiviral particles, cell transduction, as well as biosafety considerations and procedures have been described previously [11, 13, 26].

### Liver samples and clinical data

Liver tissues were treated and samples were clinically, histologically, and genetically characterized as previously described [11]. All samples were from patients recruited in accordance with French law and institutional ethical guidelines. Two sets of liver samples (set 1: 98 HCC and 19 NTL samples, Supplementary Table 4; set 2: 19 HCC and their corresponding NTL samples [11]) were collected from patients surgically treated at French University Hospitals.

### Small RNAs, miRNA mimic library and cell transfection

The *GPC3* small interfering RNA (5’-UUCUUGAGCAGCAUGUUGG-3’, Eurofins Genomics, Belgium), miRNA mimics and hairpin inhibitors (Qiagen, Sigma-Aldrich, Exiqon, Thermo Scientific Products) were used for transfecting cells as described previously [11, 13, 26]. The Human miScript miRNA Mimic 96 Set miRBase V17.0 (Qiagen, Courtaboeuf, France) was used in the DF-FunREG screening.

### DF-FunREG screening, FunREG analyses and real-time quantitative PCR and RT-PCR

FunREG and DF-FunREG analyses were performed three days after transfection as previously described [11, 13, 26] with few modifications including fluorescence measurements using the Envision multiplate reader (Perkin Elmer). Real-time quantitative PCR and RT-PCR procedures and *GPC3* primers were as described elsewhere [13]. Taqman microRNA assays (Applied Biosystems) were used to quantify the relative expression levels of mature miRNAs in the first set of 117 liver samples. MiScript Sybergreen assays (Qiagen) were used to quantify the absolute expression of mature miRNAs in the second set of 38 liver paired samples.

### Antibodies, western blot analysis, and flow cytometry

Western blot procedure has been described previously [13]. Specific protein signal was normalized to the amount of total proteins (SYPRO Ruby, Sigma-Aldrich, Lyon, France). The rabbit monoclonal anti-GPC3 (EPR5547, 1:5,000) antibody was purchased from Abcam. The mouse monoclonal anti-GAPDH (FL-335, 1:2,000) and anti-Cyclin D1 (sc-20044, 1:500) antibodies were from Santa Cruz. The mouse monoclonal anti-β-Catenin (C-14, 1:4,000) antibody was from BD Biosciences. The anti-human GPC3-Allophycocianin (APC) monoclonal antibody and IgG2a-APC isotype control were from R&D systems (Abingdon, UK). Immunofluorescence staining were performed as follow.

For flow cytometry analyses, Huh7 cells were washed in PBS, detached with PBS/EDTA, collected and incubated with the fluorescent anti-GPC3 or control antibody. Expression of the membrane GPC3 protein was analyzed by FACS. Cells incubated with the IgG2a-APC isotype control were used as negative control to gate the eGFP-positive cell populations and to measure the basal Mean Fluorescence Intensity of the whole cell population.

### Immunofluorescence assays

For immunostainings, 30,000 cells were transfected with control and miR-4510 and seeded on coverslips in 12-well plates in a volume of 1 ml. Three days later, cells were fixed with 4% PFA for 30 min and permeabilized with 0.1% Triton X-100 in PBS. Blocking was performed with 5% BSA in PBS for 30min. Primary antibody of mouse-anti-beta-catenin (C-14, BD Transduction Laboratories) was diluted to 1:200 in 5% BSA/PBS solution and incubated for 45 min. The secondary antibody Donkey anti-mouse IgG (H+L) 488 (Interchim Fluoprobes, #FP-SA4110) was diluted to 1:200 in blocking solution in presence of Hoechst dye (1:20,000) and incubated for 45min. Coverslips were washed and mounted on slides with Fluoromount G (Interchim). Slides were analyzed using confocal fluorescence microscopy (Leica SP5).

### Cell growth and proliferation assays

Cell growth was measured with the *In vitro* Toxicology assay kit (Sigma) as described previously [11], except that absorbance at 492 nm was measured using the CLARIOstar multiplate reader (BMG Labtech). For cell counting experiments, 200 000 cells were transfected and seeded into 6-well plates in a volume of 2.5 ml. Three days later total cells were counted using a Malassez cell. For the rescue experiments Huh7 cell lines ectopically expressing a *GPC3* transgene lacking the UTRs (pL-hGPC3) or a control empty transgene (pTRIP-0 plasmid) were established by lentiviral transduction. Then cells were transfected by miR-4510 or Ctrl. Three days later cells were counted and GPC3 protein expression was analyzed by Western blotting.

### Cell cycle assay

Cell cycle was studied with the APC BrdU flow kit from BD Pharmingen according to manufacturer's instruction. Briefly, 200 000 cells were transfected and seeded into 6-well plates in a volume of 2.5 ml. Three days later BrdU was added in each well and incorporated into newly synthesized DNA by cells entering and progressing through the S phase of the cell cycle. The incorporated BrdU was detected using an APC anti-BrdU fluorescent antibody and the levels of cell-associated BrdU were then measured by FACS.

### Cell death and caspase assays

Prior to apoptosis detection 200 000 cells were transfected and seeded into 6-well plates in a volume of 2.5 ml. Three days later total cells were collected and apoptosis was analyzed using the Annexin V-PE/7-Amino-Actinomycin (AAD) apoptosis detection kit (BD Pharmingen). Viable cells with intact membranes exclude 7-ADD and are PE Annexin V negative. Fluorescence generated by the cell-bound Annexin V-PE, which measures the percentage of early apoptotic cells, and the 7AAD, which measures the percentage of late apoptotic cells, were analyzed by FACS. Total apoptosis was calculated by adding the percentage of late apoptotic cells (Annexin V-PE ^High^ / 7ADD ^High^) and the percentage of early apoptotic cells (Annexin V-PE ^High^ / 7ADD ^Low^). Activities of Caspases 3 and 7 were measured using the Luminescent Caspase-Glo 3/7 assay from Promega as described previously [11], except that luminescence was measured using the CLARIOstar multiplate reader (BMG Labtech).

### Wnt transcriptional activity and associated reagents

Wnt transcriptional activity was assessed using the TOPflash/FOPflash assay. Firstly 200 000 Huh7 cells were transfected with Ctrl or miR-4510 and seeded into 6-well plates in a volume of 2.5 ml. Cells were collected two days later, 10 000 cells were seeded into 96-well plates in a volume of 100 μL and transfected with the control plasmid pRL-TK-Renilla (Promega) and either the TOPflash or FOPflash plasmids kindly provided by Hans Clevers [45]. Cells were lysed 24 hours later and luciferase activity was measured using the Dual-Luciferase®Reporter Assay System (Promega) according to manufacturer's instructions. The expression of 84 genes related to WNT-mediated signal transduction was estimated using Human Wnt Signaling Targets RT^2^ Profiler PCR Array (Qiagen) in Huh7 cells 72hr after transfection.

### Chick CAM assays

Animal procedures were carried out in agreement with the European (directive 2010/63/UE) and French (decree 2013-118) guidelines. Embryos were received at the stage of segmentation and then incubated at 37.4°C at 70% humidity. At day three of development, the eggshell was opened on the top and the opening sealed with medical-grade Durapore tape. When embryos were at day 9 of development, Huh7 cells were transfected with either control or miR-4510 as described above. The next day cells were washed in PBS and 2 million Huh7 cells were deposited on the CAM, in the center of a Thermanox plastic ring. Photographs of the tumor growth were taken every day until day sixteen using a stereomicroscope (SMZ745T) and a camera (DS-Fi2, Nikon) and then analyzed with the NSI Element D software. Three and 6 days after deposition of tumor cells CAMs were fixed and processed for histology. Tumor-containing CAM were cut in 4 μm-thick sections and stained with Eosin-hematoxylin or rabbit polyclonal anti-cleaved Caspase-3 antibody (AF835; 1:200) (R&D systems), mouse monoclonal anti-GPC3 (C-1G12; 1:50) antibody (Zytomed) or a mouse monoclonal anti-Ki-67 (MIB-1; 1:75) antibody (Dako). Finally, tumor-CAM sections were scanned using a Hamamatsu Nanozoomer 2.0HT (Bordeaux Imaging Center, Bordeaux University).

### Bioinformatic tools

Different algorithms of prediction were used to investigate target:miRNA interactions including TargetScan, miRDB, TargetMiner, miRanda, RNA Hybrid, PICTAR5, DIANAmt and Diana lab. The list of miR-4510-target genes generated from miRDB was imported to Ingenuity Pathways Analysis (IPA) to investigate the cellular functions and molecular pathways of miR-4510 target genes.

### Statistical analyses

Statistical analyses were performed using GraphPad Prism 6.0 software. Analyses with the Mann-Whitney and ANOVA tests were done as described elsewhere [11, 13]. When experiment contained two paired groups, the two-tailed Wilcoxon matched-pairs signed ranked test was used. When experiment contained two groups of categorical variable (e.g. bleeding versus no bleeding), the two-sided Fisher's exact test was used. The p-value is indicated at the bottom of each figure legend. The ANOVA test was followed by the Dunnett's multiple-comparison post-test when all data were compared to control or by the Tukey's multiple-comparison post-test when all data were compared. In each figure the number of independent experiments (n) and the ANOVA p-value is indicated in brackets. Results were considered significant when p < 0.05. For all data in figures, *: p < 0.05, **: p < 0.01, ***: p < 0.001.

## SUPPLEMENTARY MATERIALS FIGURES AND TABLES





## Abbreviations

APC: Allophycocianin
CAM: chick chorioallantoic membrane
CDH1: E-cadherin
Ctrl: control RNA
DF-FunREG: Dual Fluorescence-FunREG
FACS: Fluorescence-activated cell sorting
FBS: fetal bovine serum
GPC3: Glypican-3
HBL: hepatoblastoma
HCC: hepatocellular carcinoma
IF: immunofluorescence
IHC: immunohistochemistry
miRNA: microRNA
NTL: non-tumorous liver
ORF: open reading frame
SD: standard deviation
SEM: standard error of the mean
SULF2: sulfatase 2
siRNA: small interfering RNA
UTR: untranslated region
