# Supplementary material for: New tumor suppressor microRNAs target glypican-3 in human liver cancer
Source: Oncotarget. 2017 Apr 17;8(25):41211–26. doi: 10.18632/oncotarget.17162 (PMC5522324; doi:10.18632/oncotarget.17162)
Supplement: Supplementary file 1 [file oncotarget-08-41211-s001.pdf]

New tumor suppressor microRNAs target glypican-3 in human liver cancer

SUPPLEMENTARY MATERIALS

SUPPLEMENTARY FIGURES AND TABLES

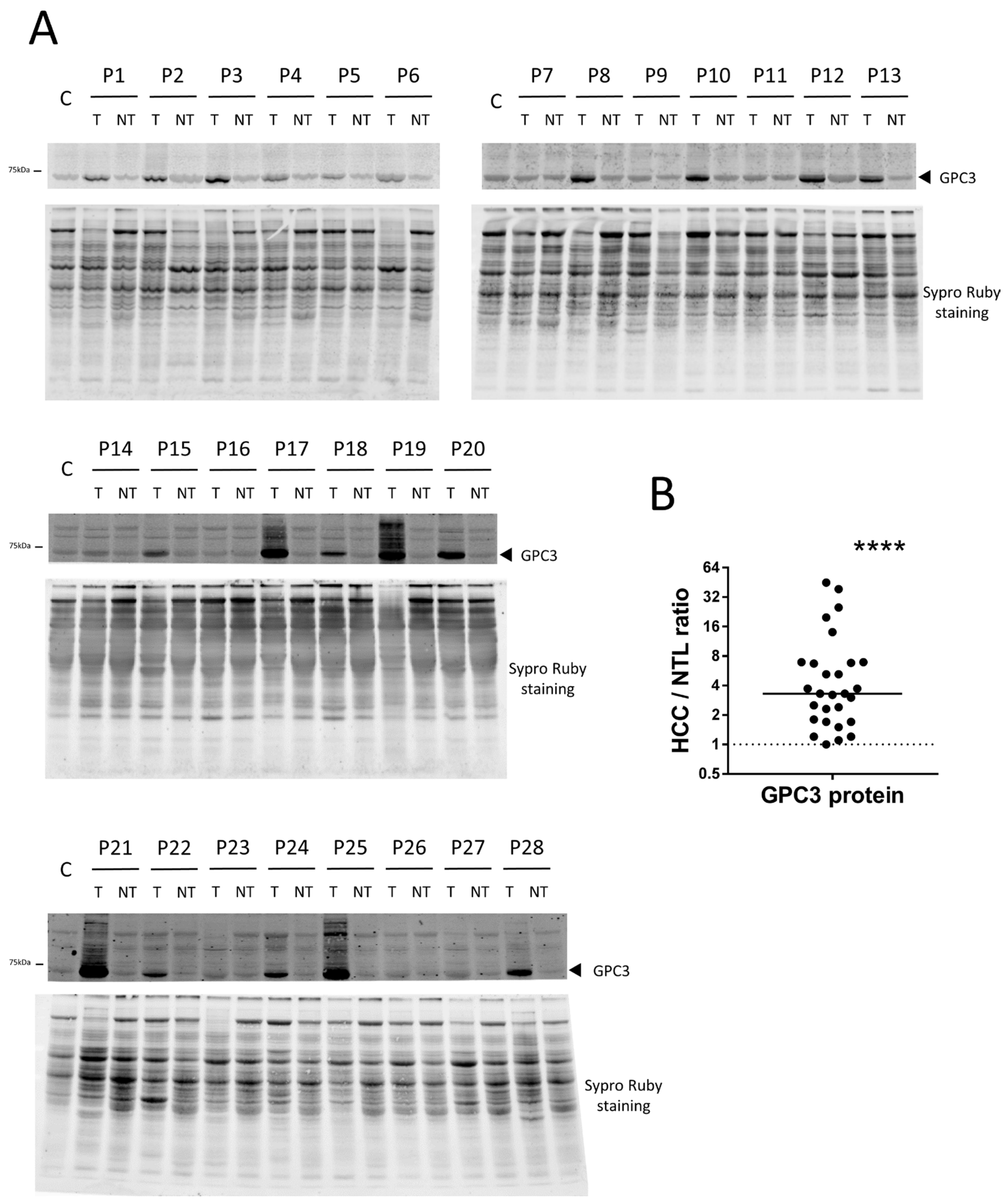

Supplementary Figure 1: Glypican-3 protein is overexpressed in HCC compared to non-tumoral liver (NTL). (A) Total proteins were extracted from 28 pairs of HCC and adjacent NTL. Then, Glypican-3 (GPC3) protein expression was quantified by western blotting using Huh7 cell extract as internal calibrator (C) and the amount of loaded total proteins for normalization. **(B)** Expression of GPC3 protein in 28 pairs of HCC and adjacent NTL. Results are presented as HCC/NTL expression ratios. The median is shown as a full line and the reference ratio value "1" is shown as a dotted line. The statistical analyses were done with the two-tailed Wilcoxon matched-pairs signed ranked test. ****: $p < 0.0001$.

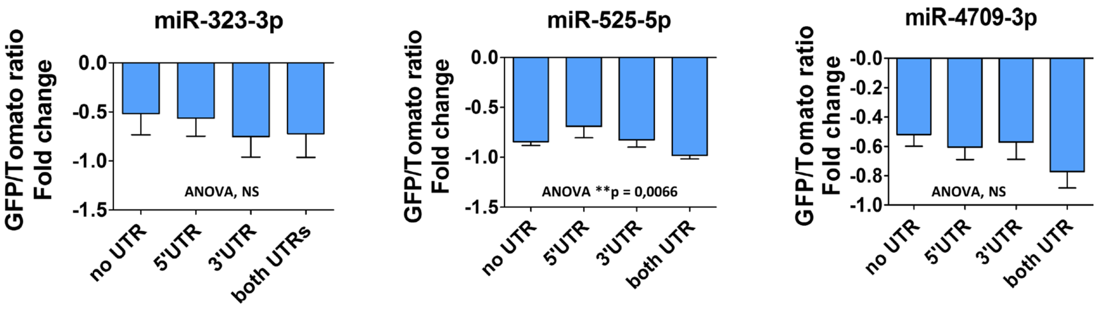

Supplementary Figure 2: The secondary step of the DF-FunREG screening eliminates three false-positive miRNAs. According to the DF-FunREG screening, three miRNAs, which decreased the GFP/Tomato ratio fold change independently of *GPC3* UTRs, were eliminated as false positive candidates. Bars represent mean + SD (n=3). Otherwise stated, for all data in this figure and the following, the ANOVA test was followed by a Dunnett's multiple comparison posttest. When significant, ANOVA p value is as shown on the corresponding graph. NS, not significant.

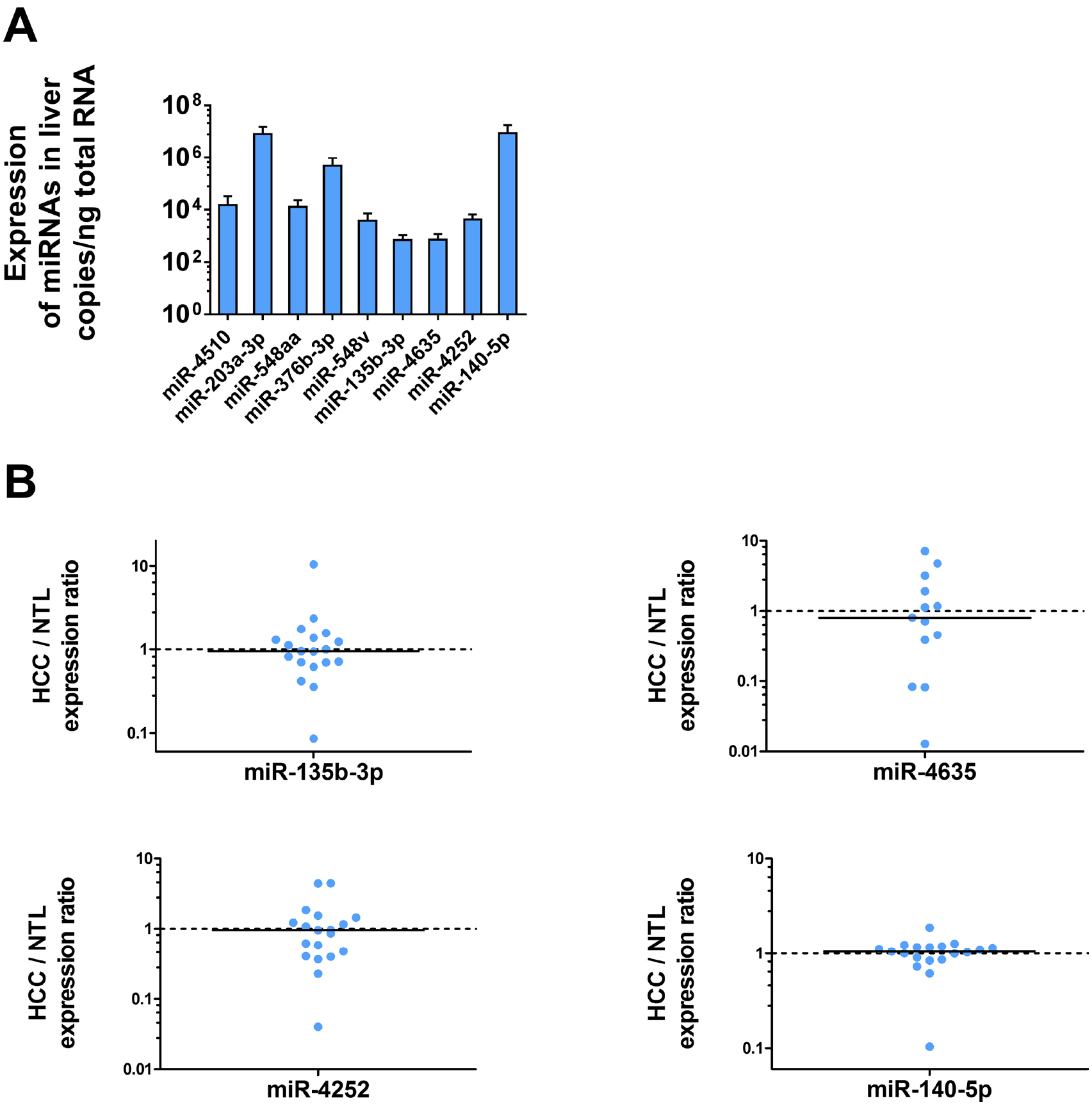

Supplementary Figure 3: Expression of *GPC3*-regulating miRNAs in normal and tumoral livers. **(A)** Expression of *GPC3*-regulating miRNAs in NTL. Results are expressed as copies per µg of total RNA. Bars represent means + SEM. MiR-4460 was not detected. **(B)** Relative expression of the corresponding miRNAs in 19 pairs of HCC and adjacent NTL samples. Results are presented as HCC/NTL expression ratios. The median is shown as a full line and the reference ratio value "1" is shown as a dotted line. Two-tailed Wilcoxon matched-pairs signed ranked test.

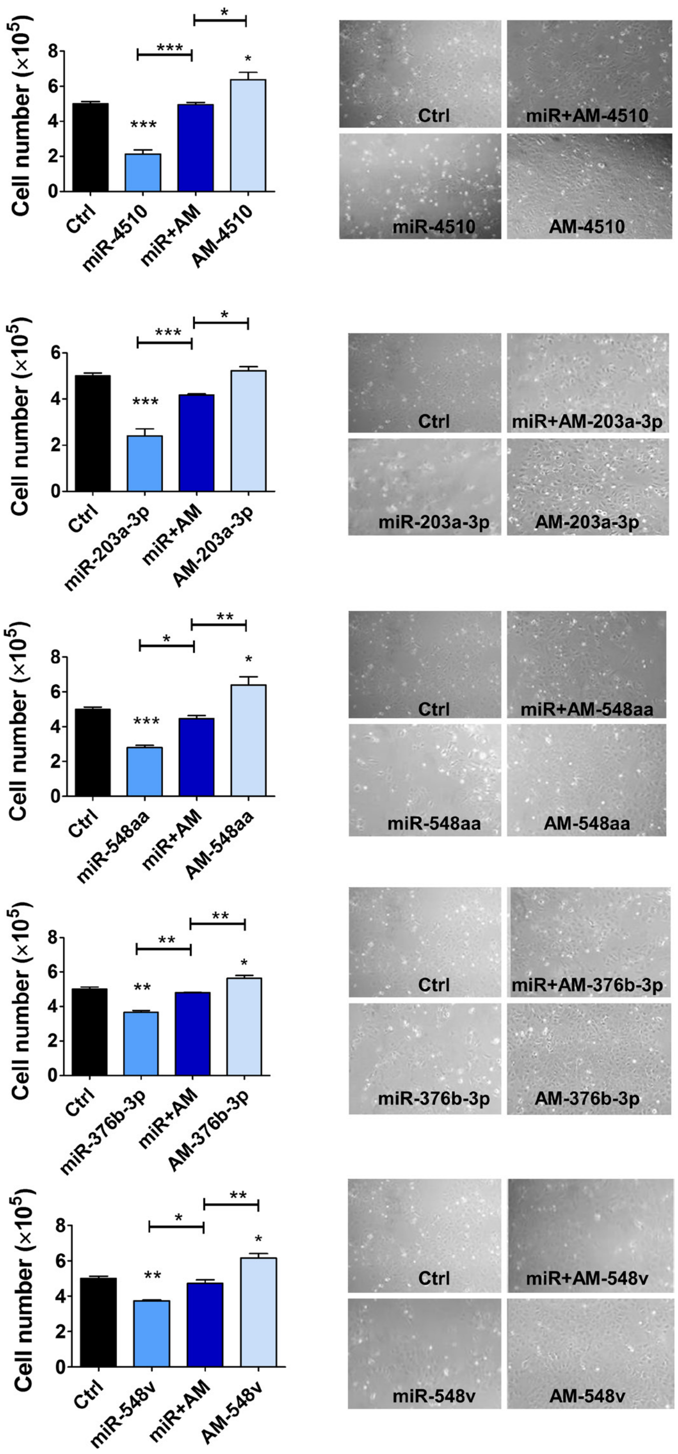

Supplementary Figure 4: Functional specificity of *GPC3*-regulating miRNAs on Huh7 cell proliferation. Three days after transfection with Ctrl, the indicated miRNA and/or a specific anti-miR (AM), Huh7 cells were counted (left graph). Bars represent means + SEM (n=3, ANOVA p<0.0001). Pictures of cultured cells three days after transfection are shown on the right panel (Representative of 3 independent experiments). Dunnett's multiple-comparison post-test: *p<0.05, **p<0.01, ***p<0.001.

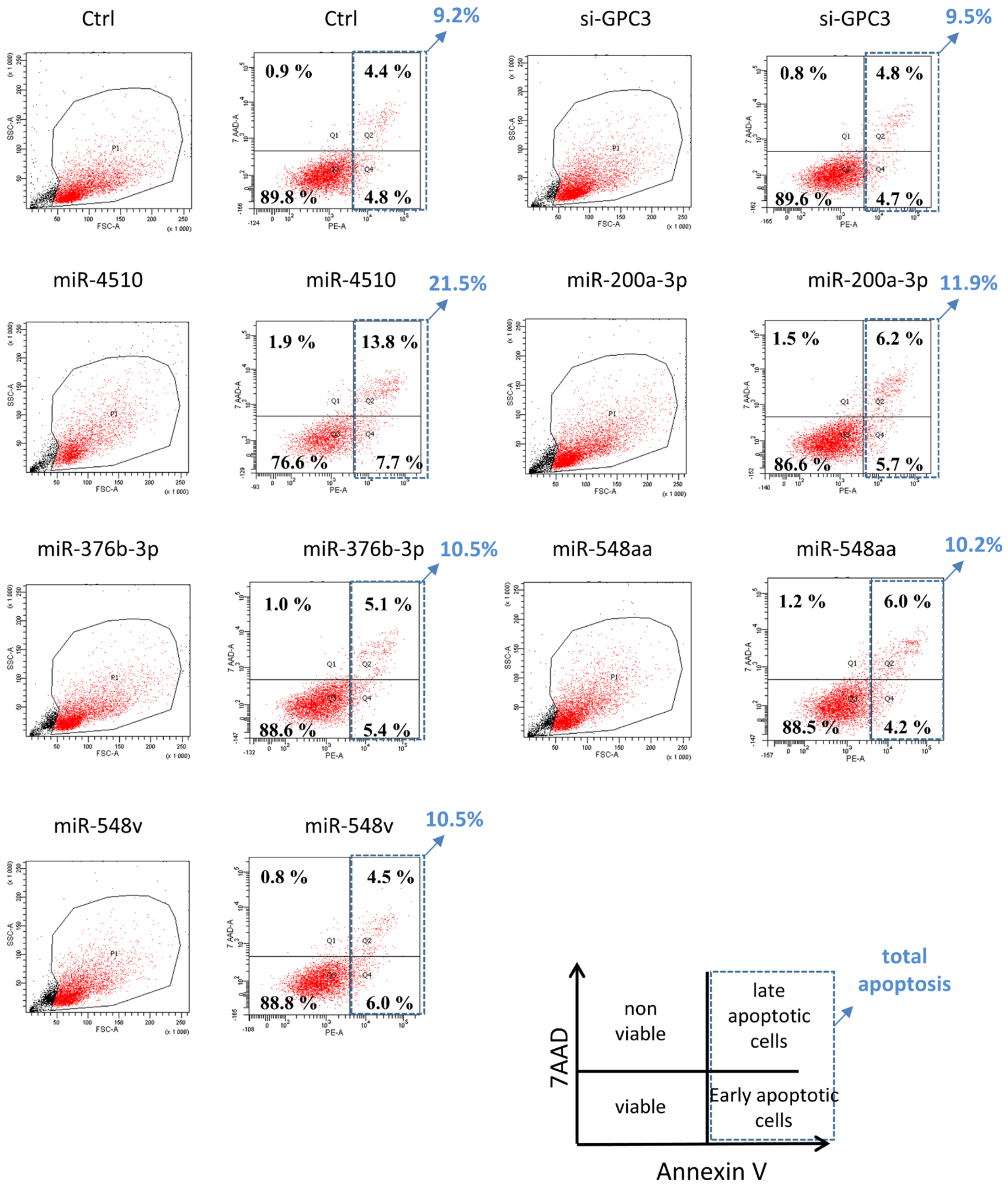

Supplementary Figure 5: Only miR-4510 induces apoptosis of Huh7 cells. Representative data of Annexin V/7AAD staining measured by flow cytometry in Huh7 cells transfected with the indicated small RNAs. As shown in the bottom right panel, total apoptosis was calculated by adding the percentage of late apoptotic cells (Annexin V-PE ^{High} / 7ADD^{High}) and the percentage of early apoptotic cells (Annexin V-PE ^{High} / 7ADD^{Low}). For each small RNA, the left graph represent the cell population selected to analyze apoptosis in the right graph. Q1, non-viable cells; Q2, late apoptotic cells; Q3: viable cells; Q4: early apoptotic cells.

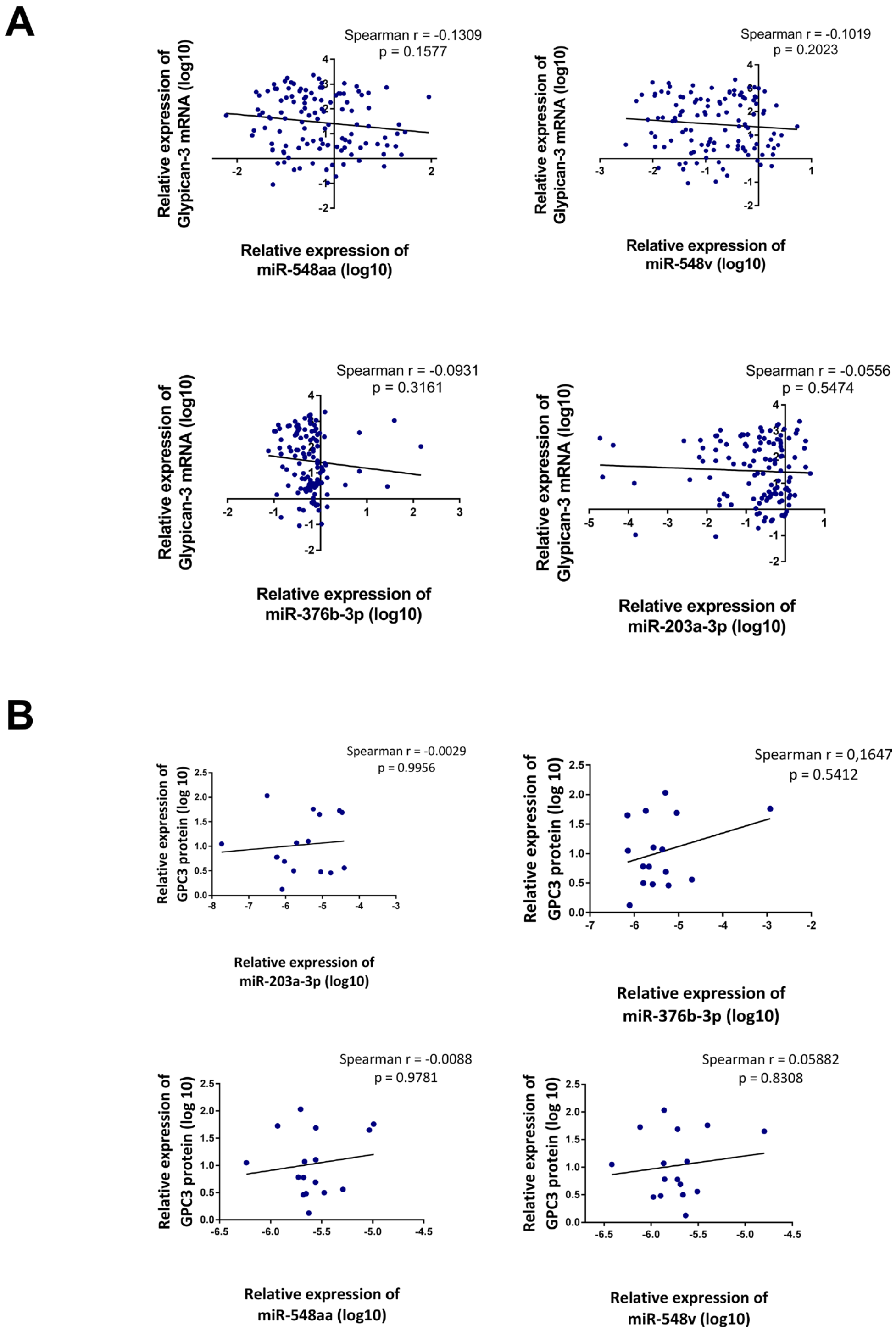

Supplementary Figure 6: The amount of miR-203a-3p, miR-376b-3p, miR-548v and miR-548aa does not correlate with GPC3 expression in patient samples. **(A)** Inverse correlation between *GPC3* mRNA level and indicated miRNA expression measured by real-time quantitative RT-PCR in 98 liver samples. **(B)** Inverse correlation between GPC3 protein level and indicated miRNA expression measured in 16 HCC by immunoblotting and real-time quantitative RT-PCR, respectively. Spearman r correlations and p values are as shown on corresponding graph.

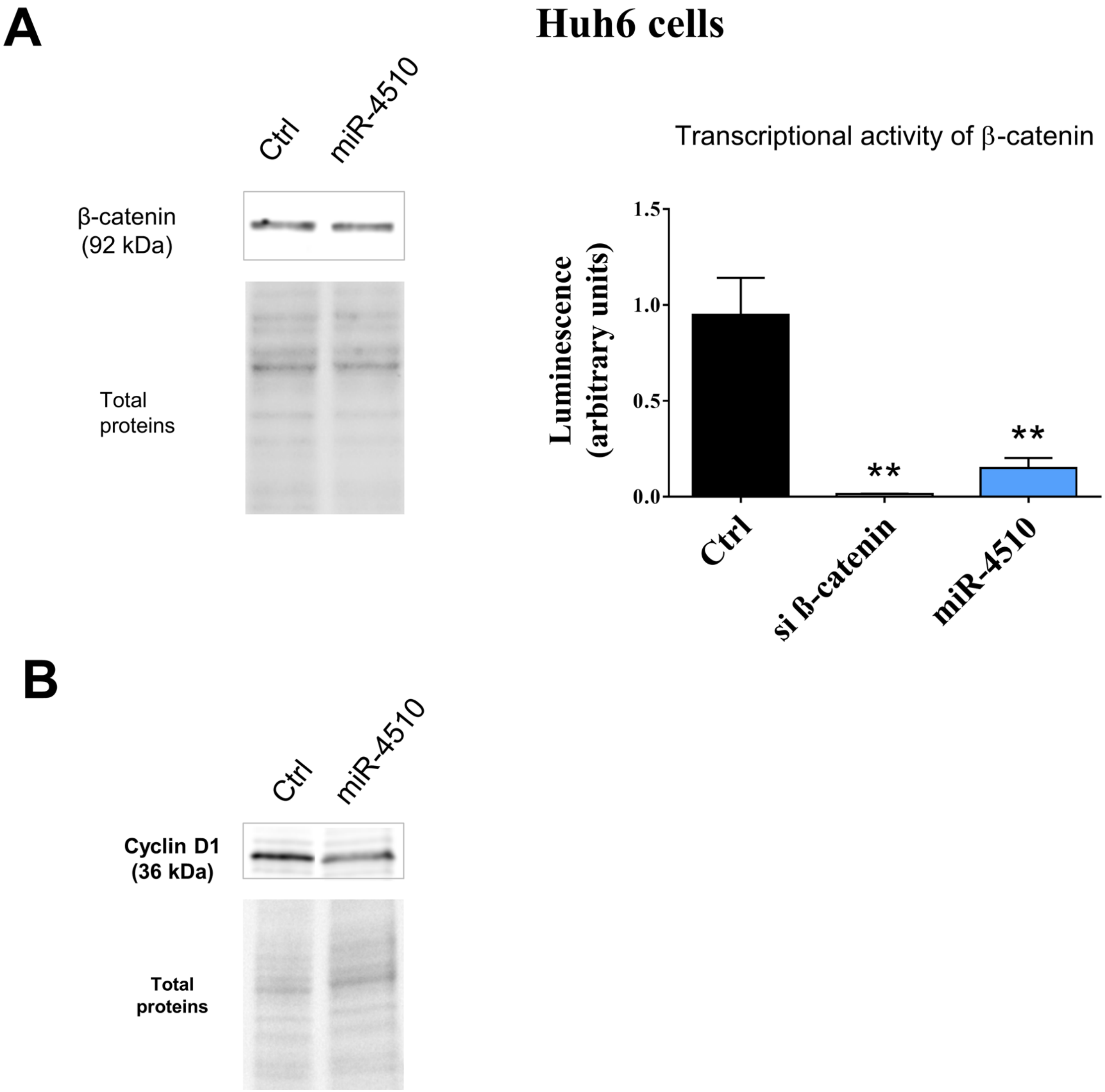

Supplementary Figure 7: Effect of miR-4510 on Wnt/β-catenin pathway and Cyclin D1 in Huh6 cells. (A) Left panel: The expression of β-catenin was assessed by Western blotting in Huh6 cells 72hr after transfection with miR-4510 or a control RNA (Ctrl). One representative blot of 3 independent experiments is shown. Protein size is shown in brackets on the left of the blot. Right panel: The transcriptional activity of β-catenin was measured by TOPflash/FOPflash assay 72hr after transfection with si-β-catenin as positive control or miR-4510. Bars represent means + SEM (n=3, ANOVA $p<0.0001$). **(B)** The expression of Cyclin D1 protein was assessed by Western blotting in Huh6 cells 72hr after transfection with miR-4510 or a control RNA (Ctrl). One representative blot of 3 independent experiments is shown. Protein size is shown in brackets on the left of the blot. **(A-B)** All cropped blots retained at least 6 bandwidths above and below the bands. SYPRO Ruby-labeled proteins cropped blots correspond to the middle part of the labeled membrane. Dunnett's multiple-comparison post-test: **: $p < 0.01$.

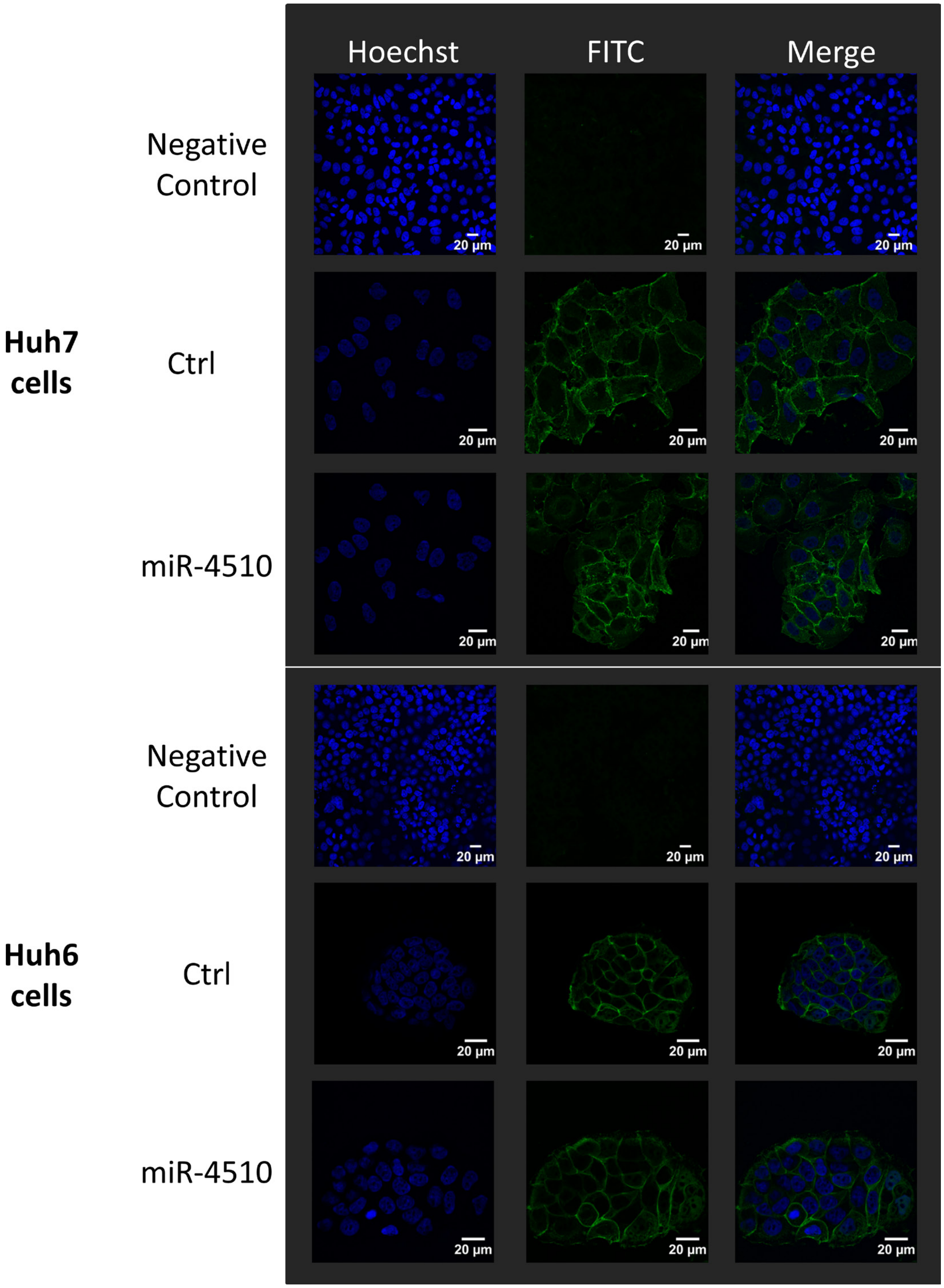

Supplementary Figure 8: MiR-4510 does not affect the subcellular localization of β-catenin protein. The subcellular distribution of β-catenin protein was determined by immunofluorescence staining in Huh7 and Huh6 cells 72hr after transfection with miR-4510 or a control RNA (Ctrl). Representative immunofluorescence staining for β-catenin protein in Huh7 (top panels) and Huh6 (bottom panels) cells (confocal microscopy). Scale bars, 20μm.

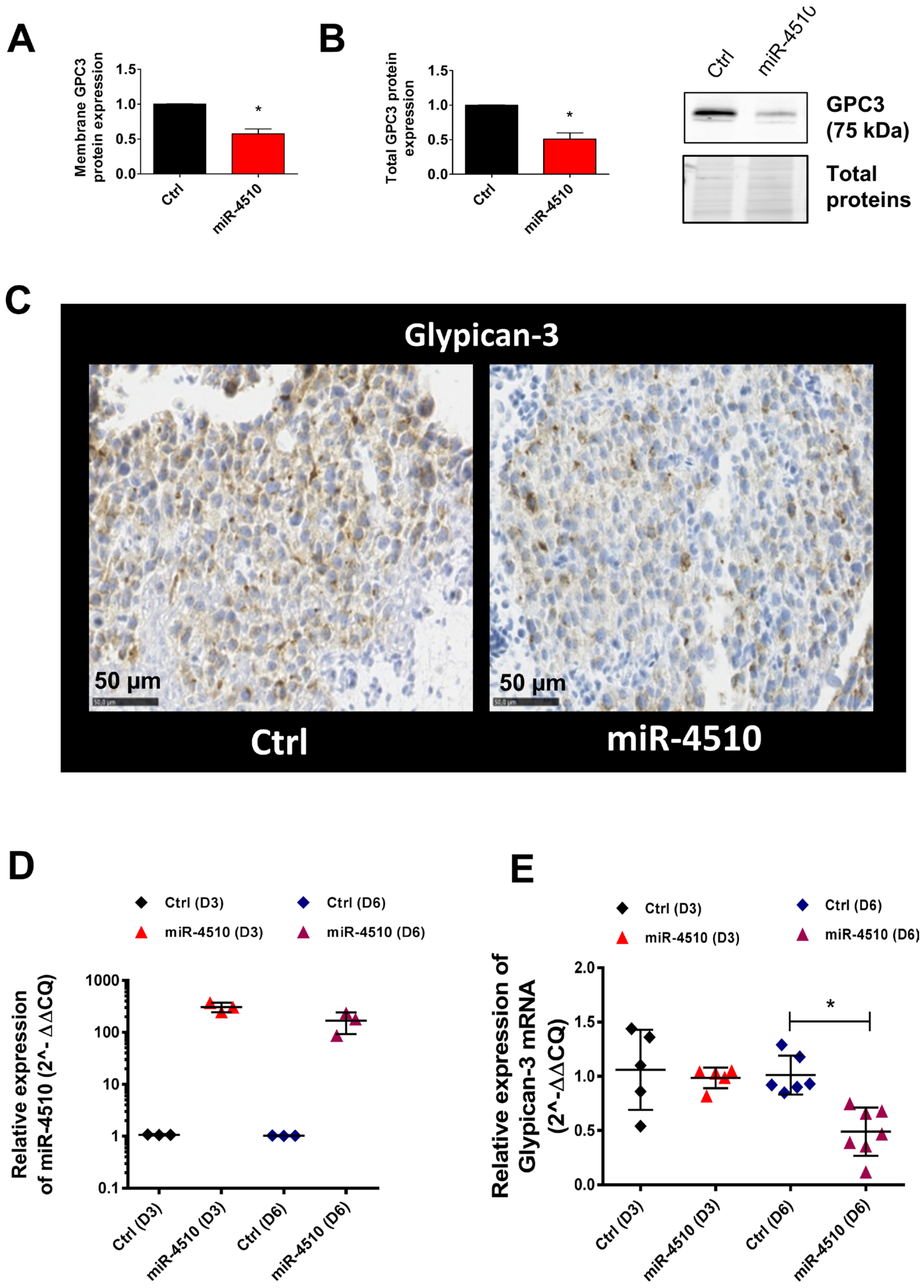

Supplementary Figure 9: MiR-4510 inhibits GPC3 expression *in vivo*. (A-B) Before implantation on CAM, GPC3 expression was measured in cultured Huh7 cells by flow cytometry **(A)** and Western blotting (**B**) 24 hours after transfection. Bars represent means + SEM (n=3). **(C)** GPC3 protein expression was measured in CAM-engrafted Huh7 cells by immunohistochemistry 3 days after implantation. One representative GPC3-stained section is shown (n=9). Scale bars, 50µm. **(D-E)** MiR-4510 and *GPC3* mRNA expression in CAM assay. MiR-4510 **(A)** and *GPC3* mRNA **(B)** expression was measured in CAM-engrafted Huh7 cells by real-time quantitative RT-PCR 3 and 6 days after implantation (miR-4510: n = 3; *GPC3* mRNA: n = 5 to 7). MiR-4510 expression was normalized to the amount of snoRNA-43, -44 and -73 and *GPC3* mRNA was normalized to the amount of 18s RNA. Mann-Whitney test, *p<0.05.

Supplementary Table 1: Primary step data of DF-FunREG screening. Standardized GFP/TOM ratio fold change mediated by each miRNA of the Human miRBase V17.0 miScript miRNA Mimic 96 Set (Qiagen) (three independent experiments are shown). The twenty miRNAs retained in the primary screen are shown in grey box

See Supplementary File 1

Supplementary Table 2: Result summary of the DF-FunREG screening and validation experiments on endogenous Glypican-3 expression

miRNA	Dual-Fluorescence FunREG screening			Effect on Endogenous Glypican-3			Algorithm of prediction
	3'-UTR	5'-UTR	3'+ 5'-UTR	Total	Membrane	mRNA	
miR-135b-3p	✓	-	-	✓	✓	-	RNAhybrid, Targetscan, miRanda, PICTAR5
miR-140-5p	✓	-	-	✓ (+)	✓ (+)	-	Targetscan, miRanda, PICTAR5, DIANAmt
miR-193a-3p	✓	-	-	-	-	ND	-
miR-203a-3p	-	-	✓	✓	-	✓ (+)	PICTAR5
miR-300	✓	-	-	-	-	ND	-
miR-376b-3p	✓	-	-	✓	✓	-	-
miR-4252	✓	-	-	✓	✓	-	-
miR-4460	-	-	✓	✓ (+)	✓ (+)	-	-
miR-4510	✓	-	-	✓	✓	✓	RNAhybrid, Targetscan
miR-4635	-	✓	-	✓	✓	-	RNAhybrid
miR-548aa	-	✓	-	✓	✓	-	-
miR-548v	-	-	✓	-	✓	-	-
miR-593-5p	✓	-	-	-	-	ND	-
miR-675	✓	-	-	-	-	ND	-

"✓": Negative effect; "✓ (+)": Positive effect; "-": no effect or no predicted interaction (right column); "ND": not determined.

Supplementary Table 3: Correlation between expression of *GPC3*-regulating miRNAs and clinical parameters. NS: not significant

	miR-4510	miR-203a-3p	miR-548aa	miR-376b-3p	miR-548v
**Gender**<br>F (17, 17%), M (85, 83%)	NS	NS	NS	NS	NS
**Age (years)**<br><63.5 (47, 46%), >=63.5 (55, 54%)	NS	NS	NS	NS	NS
**Ethiology**<br>AL (31, 31%) AL HBV (3, 3%) AL HBV HCV (1, 1%) AL HBV METAB (1, 1%) AL HCV (1, 1%) AL HM (3, 3%) AL METAB (1, 1%) HBV (5, 5%) HBV HCV (19, 19%) HBV OTHER (3, 3%) HCV (2, 2%) HM (11.5, 11.5%) METAB (4, 4%)	NS	NS	NS	NS	NS
**Number of distal nodules**	NS	NS	NS	NS	NS
**Poor prognosis**<br>good (11,46%), poor (13,54%)	NS	NS	NS	NS	NS
**Child Pugh (classe)**<br>A (87, 95%), B (5, 5%)	NS	NS	NS	NS	NS
**Ascites**<br>No (62, 94%), Yes (4, 6%)	NS	NS	NS	NS	NS
**AlphaFoeto (ng/ml)**<br><=20 (41, 43%), >20 (52, 54%)	NS	NS	NS	NS	NS
**Largest nodule diameter (cm)**<br><=50 (41, 41%), >50 (60, 59%)	NS	NS	NS	NS	NS
**Satellite nodules**<br>No (52, 52%), Yes (48, 48%)	NS	NS	**p=0,05<br>(↗ No)**	NS	NS
**Vascular invasion**<br>No (43, 43%), Yes (57, 57%)	NS	NS	NS	NS	NS
**Portal vein microscopic invasion**<br>No (43, 43%), Yes (56, 57%)	NS	NS	NS	NS	NS
**EdmoNSon grade**<br>I-II (45, 45%), III-IV (54, 55%)	NS	NS	NS	NS	NS
**Tumoral capsule**<br>Absent (25, 25%), Intact (16, 16%), Invaded (59, 59%)	NS	NS	NS	NS	NS
**Normal liver histology**<br>CIRR (34, 34%), NL (14, 14%), PF (17, 17%), HCA (36, 36%)	NS	NS	NS	NS	NS
**Metavir-fibrosis (non-tumorous liver)**<br>F0 (11, 11%), F1 (19, 20%) F2 (24, 25%) F3 (9, 9%) F4 (33,34%)	NS	NS	NS	NS	NS
**X5 gene score**<br>Good prognosis (45, 49%), Poor prognosis (47, 51%)	NS	NS	NS	NS	NS
**Mutation CTNNB1 status**<br>Mu (22, 41%), NMu (32, 59%)	NS	NS	NS	NS	NS
**Mutation P53 status**<br>Mu (23, 23%), NMu (79, 77%)	NS	NS	NS	**p=0,02<br>(↗ NMu)**	NS

AL: alcohol; CIRR: cirrhosis; F: female; HBV: hepatitis B virus; HCA: chronic active hepatitis ; HCV: hepatitis C virus; HM: hematochromatosis; M: male; METAB: metabolic syndrome; Mu: mutated; NL: normal liver; NMu: non mutated; PF: portal fibrosis. X5_gene.score: see Nault JC et al. Gastroenterology. 2013 Jul;145(1):176-87. doi: 10.1053/j.gastro.2013.03.051.

Supplementary Table 4: Clinical and genetic characteristics in a series of 117 liver samples (19 NTL and 98 HCC)

Patient features	Features	Number of cases (%)
Gender	Male	85 (83%)
Age mean (min, max)	mean (min, max)	57,2 (18-79)
Tumor differentiation (Edmondson)	I-II	45 (45%)
	III-IV	54 (55%)
Etiology	Alcohol	45 (44,5%)
	HCV	18 (17,6%)
	HBV	29 (28,4%)
	NASH	9 (8,8%)
	hemochromatosis	6 (5,8%)
	other	12 (11,7%)
Fibrosis in non-tumor liver (METAVIR)	F0	11 (11%)
	F1	19 (20%)
	F2	24 (25%)
	F3	9 (9%)
	F4	33 (34%)
CTNNB1	Mutated	38 (38,7%)
	non-mutated	60 (61,2%)
TP53	Mutated	23 (22,5%)
	non-mutated	79 (77,4%)
5_gene_score		
	P1	45 (48,9%)
	P2	47 (51%)
