# Supplementary material for: New tumor suppressor microRNAs target glypican-3 in human liver cancer
Source: Oncotarget. 2017 Apr 17;8(25):41211–26. doi: 10.18632/oncotarget.17162 (PMC5522324; doi:10.18632/oncotarget.17162)
Supplement: Supplementary file 2 [file oncotarget-08-41211-s002.docx]

**Supplementary Table 1: Primary step data of DF-FunREG screening.**

| **miRNA (miRBase V17.0)** | **Exp. 1** | **Exp. 2** | **Exp. 3** | **Mean** | **Standard Deviation** |
| --- | --- | --- | --- | --- | --- |
| hsa-miR-96 | -1,303414383 | -1,389258797 | -1,32792291 | -1,340 | 0,044 |
| hsa-miR-1271 | -0,933269979 | -0,897464658 | -0,94902164 | -0,927 | 0,026 |
| hsa-miR-1973 | -0,758593937 | -0,848380069 | -1,04232825 | -0,883 | 0,145 |
| hsa-miR-135b* | -1,045228656 | -1,109134924 | -0,32327853 | -0,826 | 0,436 |
| hsa-miR-300 | -0,845765032 | -0,813298723 | -0,80095531 | -0,820 | 0,023 |
| hsa-miR-323-3p | -0,659034151 | -0,680049868 | -0,8072553 | -0,715 | 0,080 |
| hsa-miR-140-5p | -0,832434578 | -0,796574479 | -0,48695828 | -0,705 | 0,190 |
| hsa-miR-4635 | -0,719025713 | -0,585069223 | -0,7946114 | -0,700 | 0,106 |
| hsa-miR-193a-3p | -0,665743165 | -0,580380189 | -0,77477387 | -0,674 | 0,097 |
| hsa-miR-525-5p | -0,544206161 | -0,623096391 | -0,8122538 | -0,660 | 0,138 |
| hsa-miR-548v | -0,750752637 | -0,567689085 | -0,22309641 | -0,514 | 0,268 |
| hsa-miR-4460 | -0,375561142 | -0,761083753 | -0,82322387 | -0,653 | 0,243 |
| hsa-miR-4510 | -0,599737325 | -0,631441427 | -0,7052536 | -0,645 | 0,054 |
| hsa-miR-4252 | -0,65163481 | -0,563456173 | -0,71901582 | -0,645 | 0,078 |
| hsa-miR-548aa | -0,572316151 | -0,620000313 | -0,69645923 | -0,630 | 0,063 |
| hsa-miR-376b | -0,426888827 | -0,69869484 | -0,58067322 | -0,569 | 0,136 |
| hsa-miR-593* | -0,606356304 | 0,098051759 | -0,45934597 | -0,323 | 0,372 |
| hsa-miR-4709-3p | -0,335743657 | -0,557884945 | -0,6886117 | -0,527 | 0,178 |
| hsa-miR-675 | -0,461662847 | -0,449084363 | -0,65080509 | -0,521 | 0,113 |
| hsa-miR-203 | -0,580054574 | -0,460594901 | -0,50092461 | -0,514 | 0,061 |
| hsa-miR-432* | -0,533014316 | -0,133973065 | -0,46285086 | -0,377 | 0,213 |
| hsa-miR-1909* | -0,147740505 | -0,601909009 | -0,35804015 | -0,369 | 0,227 |
| hsa-miR-4698 | -0,493915813 | -0,397138483 | -0,53695275 | -0,476 | 0,072 |
| hsa-miR-218-1* | -0,456937198 | -0,202371174 | -0,48567061 | -0,382 | 0,156 |
| hsa-miR-488* | -0,493407614 | -0,263806148 | -0,64861247 | -0,469 | 0,194 |
| hsa-miR-93* | -0,30074609 | -0,706993632 | -0,38817433 | -0,465 | 0,214 |
| hsa-miR-4802-5p | -0,504312162 | -0,517937842 | -0,36248577 | -0,462 | 0,086 |
| hsa-miR-744 | -0,539533669 | -0,465928892 | -0,3414889 | -0,449 | 0,100 |
| hsa-miR-4425 | -0,518139249 | -0,441727199 | -0,36788722 | -0,443 | 0,075 |
| hsa-miR-23a | -0,387557114 | -0,467894538 | -0,47053399 | -0,442 | 0,047 |
| hsa-miR-943 | -0,339773742 | -0,525993552 | -0,45746988 | -0,441 | 0,094 |
| hsa-miR-4266 | -0,477938741 | -0,421109764 | -0,3983271 | -0,432 | 0,041 |
| hsa-miR-1245b-3p | -0,343770341 | -0,444287646 | -0,49633271 | -0,428 | 0,078 |
| hsa-miR-4765 | -0,399849572 | 0,79 | -0,45259405 | -0,021 | 0,703 |
| hsa-miR-585 | -0,582136362 | -0,269241707 | 0,00834914 | -0,281 | 0,295 |
| hsa-miR-3121-5p | -0,336390416 | -0,518368522 | -0,4217398 | -0,425 | 0,091 |
| hsa-miR-609 | -0,325674908 | -0,527036633 | -0,39652152 | -0,416 | 0,102 |
| hsa-miR-4764-5p | -0,334622651 | -0,386941121 | -0,51926969 | -0,414 | 0,095 |
| hsa-miR-548ad | -0,323275778 | -0,359359083 | -0,54809371 | -0,410 | 0,121 |
| hsa-miR-548z | -0,262906575 | -0,33118445 | -0,63663382 | -0,410 | 0,199 |
| hsa-miR-518f* | -0,264823924 | -1,175653318 | -0,55379726 | -0,665 | 0,465 |
| hsa-miR-4640-5p | -0,425813536 | -0,521621678 | -0,27805225 | -0,408 | 0,123 |
| hsa-miR-26b* | -0,3399427 | -0,523876759 | -0,35716804 | -0,407 | 0,102 |
| hsa-miR-4445* | -0,613979797 | 1,561562226 | -0,17192357 | 0,259 | 1,150 |
| hsa-miR-3675-3p | -0,36673942 | -0,455423009 | -0,33514601 | -0,386 | 0,062 |
| hsa-miR-526a // hsa-miR-520c-5p // hsa-miR-518d-5p | -0,429980495 | -0,20384632 | -0,52070677 | -0,385 | 0,163 |
| hsa-miR-3929 | -0,342544884 | 0,79 | -0,42650354 | 0,007 | 0,679 |
| hsa-miR-4802-3p | -0,263616501 | -0,477412463 | -0,39197122 | -0,378 | 0,108 |
| hsa-miR-3545-5p | -0,421624319 | -0,267816464 | -0,43182146 | -0,374 | 0,092 |
| hsa-miR-182 | -0,405391396 | -0,37508863 | -0,33331568 | -0,371 | 0,036 |
| hsa-miR-4649-5p | -0,367078729 | -0,248978618 | -0,49128765 | -0,369 | 0,121 |
| hsa-miR-2277-5p | -0,28013088 | -0,482450263 | -0,33629848 | -0,366 | 0,104 |
| hsa-miR-524-5p | -0,25066881 | -0,527608095 | -0,31410436 | -0,364 | 0,145 |
| hsa-miR-449c | -0,367771603 | -0,328491012 | -0,39093156 | -0,362 | 0,032 |
| hsa-miR-196a | -0,379789559 | -0,293189032 | -0,41182847 | -0,362 | 0,061 |
| hsa-miR-31* | -0,002486533 | -0,789892735 | -0,28143046 | -0,358 | 0,399 |
| hsa-miR-3120-5p | -0,286058205 | -0,309696008 | -0,47745628 | -0,358 | 0,104 |
| hsa-miR-4524 | -0,273909548 | -0,401299713 | -0,37148149 | -0,349 | 0,067 |
| hsa-miR-4540 | -0,303029025 | -0,285121615 | -0,45789127 | -0,349 | 0,095 |
| hsa-miR-154* | -0,366425028 | -0,652886102 | -0,01814716 | -0,346 | 0,318 |
| hsa-miR-2115 | -0,5546272 | -0,19418377 | -0,2838163 | -0,344 | 0,188 |
| hsa-miR-4687-5p | -0,303721673 | -0,065863999 | -0,38423399 | -0,251 | 0,166 |
| hsa-miR-16 | -0,359323844 | -0,184256514 | -0,48173217 | -0,342 | 0,150 |
| hsa-miR-4498 | -0,388140053 | -0,413956134 | -0,22278898 | -0,342 | 0,104 |
| hsa-miR-99b* | -0,155619205 | -0,477525816 | -0,38267461 | -0,339 | 0,165 |
| hsa-miR-34b* | -0,289973032 | -2,166351712 | -0,38199201 | -0,946 | 1,058 |
| hsa-miR-3117-3p | -0,351014563 | -0,56849991 | -0,0762167 | -0,332 | 0,247 |
| hsa-miR-888* | 0,144557516 | -0,791201239 |  | -0,323 | 0,662 |
| hsa-miR-613 | -0,23013858 | -0,327036689 | -0,41020948 | -0,322 | 0,090 |
| hsa-miR-3915 | -0,19675331 | -0,446609689 | 0,24452151 | -0,133 | 0,350 |
| hsa-miR-449b* | -0,163355826 | -0,600717641 | -0,1935586 | -0,319 | 0,244 |
| hsa-miR-431* | -0,261519173 | -0,314935128 | -0,37955824 | -0,319 | 0,059 |
| hsa-miR-4660 | -0,214281561 | -0,280927284 | -0,45919194 | -0,318 | 0,127 |
| hsa-miR-451b | -0,59792109 | -0,211680913 | -0,1359128 | -0,315 | 0,248 |
| hsa-miR-129* | -0,200795951 | -0,297697544 | -0,44085929 | -0,313 | 0,121 |
| hsa-miR-4474-3p | -0,314295423 | -0,202139374 | -0,42236757 | -0,313 | 0,110 |
| hsa-miR-129-3p | -0,340559797 | -0,203297339 | -0,39300236 | -0,312 | 0,098 |
| hsa-miR-17* | -0,139381789 | -0,607767444 | -0,1888525 | -0,312 | 0,257 |
| hsa-miR-876-3p | -0,313722654 | -0,161518681 | -0,46009906 | -0,312 | 0,149 |
| hsa-miR-146a | -0,284981915 | -0,256247871 | -0,3842465 | -0,308 | 0,067 |
| hsa-let-7f-1* | -0,157660228 | -0,385311004 | -0,38007681 | -0,308 | 0,130 |
| hsa-miR-548u | -0,280593693 | -0,352728603 | -0,28549813 | -0,306 | 0,040 |
| hsa-miR-7-2* | -0,238215002 | -1,986559178 | -0,37133345 | -0,865 | 0,973 |
| hsa-miR-506 | -0,076478609 | -0,434053452 | -0,39555706 | -0,302 | 0,196 |
| hsa-miR-4781-3p | -0,206707434 | -0,285428546 | -0,41230572 | -0,301 | 0,104 |
| hsa-miR-125b | -0,333514126 | -0,346563299 | -0,21389775 | -0,298 | 0,073 |
| hsa-miR-122 | -0,373607849 | -0,311948896 | -0,19811469 | -0,295 | 0,089 |
| hsa-miR-619 | -0,214140548 | -0,351623871 | -0,30779093 | -0,291 | 0,070 |
| hsa-miR-1231 | -0,240990721 | -0,285767499 | -0,34252192 | -0,290 | 0,051 |
| hsa-miR-19b-1* | -0,293818432 | -0,479022228 | -0,09581737 | -0,290 | 0,192 |
| hsa-miR-3918 | -0,158078663 | -0,404925735 | -0,30387271 | -0,289 | 0,124 |
| hsa-miR-3126-5p | -0,217533258 | -0,447490474 | -0,19934765 | -0,288 | 0,138 |
| hsa-miR-4419a | -0,156098219 | -0,19311561 | -0,50694109 | -0,285 | 0,193 |
| hsa-miR-548e | -0,158750821 | -0,179532653 | -0,51282163 | -0,284 | 0,199 |
| hsa-miR-502-5p | -0,18025288 | -0,238111329 | -0,42430423 | -0,281 | 0,128 |
| hsa-miR-3692* | -0,073622769 | -0,532663468 | -0,226723 | -0,278 | 0,234 |
| hsa-miR-744* | -0,226106627 | -0,362128657 | -0,24402089 | -0,277 | 0,074 |
| hsa-miR-4751 | -0,206793836 | -0,300425755 | -0,32375245 | -0,277 | 0,062 |
| hsa-miR-3130-3p | -0,274647755 | -0,177389702 | -0,37381927 | -0,275 | 0,098 |
| hsa-miR-3613-5p | -0,267749326 | -0,195047521 | -0,36149312 | -0,275 | 0,083 |
| hsa-miR-508-5p | -0,17055896 | -0,321227173 | -0,33152999 | -0,274 | 0,090 |
| hsa-miR-19b-2* | -0,15681314 | -0,46929723 | -0,19392138 | -0,273 | 0,171 |
| hsa-miR-4441 | -0,172410268 | -0,397887302 | -0,24540362 | -0,272 | 0,115 |
| hsa-miR-4418 | -0,40300502 | -0,238475458 | -0,17342991 | -0,272 | 0,118 |
| hsa-miR-3925-5p | -0,300525972 | -0,284228107 | -0,22875873 | -0,271 | 0,038 |
| hsa-miR-654-5p | -0,333241624 | -0,372319921 | -0,10254249 | -0,269 | 0,146 |
| hsa-let-7a* | -0,259738384 | -0,68541155 | -0,27678694 | -0,407 | 0,241 |
| hsa-miR-132* | -0,037315542 | -0,638861711 | -0,12271313 | -0,266 | 0,325 |
| hsa-miR-1285 | -0,232388341 | -0,237109141 | -0,31988145 | -0,263 | 0,049 |
| hsa-miR-4734 | -0,133366022 | -0,351578997 | -0,30145453 | -0,262 | 0,114 |
| hsa-miR-4269 | -0,198106651 | -0,196366237 | -0,39069783 | -0,262 | 0,112 |
| hsa-miR-4665-5p | -0,117244543 | -0,470317494 | -0,1938787 | -0,260 | 0,186 |
| hsa-miR-4679 | -0,183038669 | -0,335523887 | -0,23916937 | -0,253 | 0,077 |
| hsa-miR-589* | -0,230737597 | 0,416756588 | -0,26868625 | -0,028 | 0,385 |
| hsa-miR-192* | -0,202214571 | -0,231473774 | -0,31382088 | -0,249 | 0,058 |
| hsa-miR-4712-5p | -0,255853244 | -0,153477368 | -0,33715361 | -0,249 | 0,092 |
| hsa-miR-3161 | -0,165616484 | -0,202658837 | -0,36563837 | -0,245 | 0,106 |
| hsa-miR-323-5p | -0,214735092 | -0,1573304 | -0,35596528 | -0,243 | 0,102 |
| hsa-miR-4530 | -0,119898055 | -0,395026303 | -0,21160978 | -0,242 | 0,140 |
| hsa-miR-449a | -0,242665291 | -0,43113671 | -0,04991634 | -0,241 | 0,191 |
| hsa-miR-3128 | -0,077692936 | -0,230130931 | -0,412856 | -0,240 | 0,168 |
| hsa-miR-4644 | -0,118682985 | -0,323886094 | -0,27614302 | -0,240 | 0,107 |
| hsa-miR-107 | -0,272227156 | -0,306629137 | -0,13150255 | -0,237 | 0,093 |
| hsa-miR-4524* | 0,007096167 | -1,922444474 | -0,48047947 | -0,799 | 1,003 |
| hsa-miR-1185 | -0,104309485 | -0,26770765 | -0,32149868 | -0,231 | 0,113 |
| hsa-miR-4677-5p | -0,351606627 | -0,115785845 | -0,22532672 | -0,231 | 0,118 |
| hsa-miR-379* | -0,142926187 | -0,196368729 | -0,34407971 | -0,228 | 0,104 |
| hsa-miR-1304 | -0,381341899 | -0,208444405 | -0,09229133 | -0,227 | 0,145 |
| hsa-miR-1307 | -0,162155832 | -0,159437586 | -0,35878608 | -0,227 | 0,114 |
| hsa-miR-577 | -0,058587467 | -0,278878506 | -0,34076781 | -0,226 | 0,148 |
| hsa-miR-518a-5p // hsa-miR-527 | -0,145553263 | -0,226631337 | -0,30132928 | -0,225 | 0,078 |
| hsa-miR-510 | -0,170839964 | -0,185329598 | -0,31535548 | -0,224 | 0,080 |
| hsa-miR-147b | -0,173819779 | -0,235839134 | -0,25956558 | -0,223 | 0,044 |
| hsa-miR-411* | -0,133653763 | -0,363284263 | -0,17113924 | -0,223 | 0,123 |
| hsa-miR-218 | -0,302097111 | -0,130563452 | -0,23513741 | -0,223 | 0,086 |
| hsa-miR-96* | -0,174159448 | -1,279418439 | -0,27053459 | -0,575 | 0,612 |
| hsa-let-7b* | -0,05114713 | -0,287447555 | -0,32712573 | -0,222 | 0,149 |
| hsa-miR-3150a-3p | -0,133796827 | -0,305890674 | -0,22589628 | -0,222 | 0,086 |
| hsa-miR-500a* | 0,035417572 | -0,530998305 | -0,16710656 | -0,221 | 0,287 |
| hsa-miR-4440 | -0,100055972 | -0,276771069 | -0,28164446 | -0,219 | 0,103 |
| hsa-miR-425* | -0,070695868 | -0,333524676 | -0,24988798 | -0,218 | 0,134 |
| hsa-miR-130b* | -0,124731896 | 0,495533289 | -0,30530066 | 0,022 | 0,420 |
| hsa-miR-196a* | -0,101728548 | -0,255304889 | -0,28445947 | -0,214 | 0,098 |
| hsa-miR-181c* | -0,015613026 | -0,410896799 |  | -0,213 | 0,280 |
| hsa-miR-4701-3p | -0,050050432 | -0,298622307 | -0,288234 | -0,212 | 0,141 |
| hsa-miR-4739 | -0,093880941 | -0,244573086 | -0,29437128 | -0,211 | 0,104 |
| hsa-miR-3187-5p | -0,188126771 | 0,9 | -0,23277394 | 0,160 | 0,642 |
| hsa-miR-93 | -0,270227655 | -0,157102103 | -0,20378818 | -0,210 | 0,057 |
| hsa-miR-4437 | -0,024572438 | -0,292923063 | -0,31070626 | -0,209 | 0,160 |
| hsa-miR-3186-3p | -0,055052611 | -0,236106101 | -0,33646404 | -0,209 | 0,143 |
| hsa-miR-145* | -0,07054371 | -0,324389536 | -0,23262109 | -0,209 | 0,129 |
| hsa-miR-151-5p | -0,207248168 | -0,147664413 | -0,26845925 | -0,208 | 0,060 |
| hsa-miR-1914* | 0,104545004 | -0,591345933 | -0,13448094 | -0,207 | 0,354 |
| hsa-let-7b | -0,218685516 | -0,193327838 | -0,20537191 | -0,206 | 0,013 |
| hsa-miR-3165 | -0,143851183 | -0,301177545 | -0,17104115 | -0,205 | 0,084 |
| hsa-miR-580 | -0,124844382 | -0,139814123 | -0,34990441 | -0,205 | 0,126 |
| hsa-miR-3682-5p | -0,124074218 | -0,209288365 | -0,27726445 | -0,204 | 0,077 |
| hsa-miR-125b-2* | -0,061989664 | -0,342479473 | -0,20382739 | -0,203 | 0,140 |
| hsa-miR-4434 | -0,298732422 | -0,126822575 | -0,17821477 | -0,201 | 0,088 |
| hsa-let-7i | -0,159004386 | -0,115775356 | -0,32814573 | -0,201 | 0,112 |
| hsa-miR-4779 | -0,099803773 | -0,143064341 | -0,35866335 | -0,201 | 0,139 |
| hsa-miR-196b | -0,297661736 | -0,223710972 | -0,07995992 | -0,200 | 0,111 |
| hsa-miR-29b-1* | 0,10716177 | -0,647457595 | -0,06048332 | -0,200 | 0,396 |
| hsa-miR-4784 | -0,085882021 | -0,26347118 | -0,25045937 | -0,200 | 0,099 |
| hsa-miR-4700-3p | -0,13383586 | -0,180647572 | -0,28302688 | -0,199 | 0,076 |
| hsa-miR-27b* | -0,313131002 | 0,569222213 | -0,08431775 | 0,057 | 0,458 |
| hsa-miR-92a-2* | -0,156461428 | -0,240586723 |  | -0,199 | 0,059 |
| hsa-miR-193a-5p | -0,183974136 | -0,218248565 | -0,19181461 | -0,198 | 0,018 |
| hsa-let-7e | -0,128184628 | -0,258845723 | -0,20370361 | -0,197 | 0,066 |
| hsa-miR-4728-5p | -0,08932393 | -0,405605201 | -0,09391568 | -0,196 | 0,181 |
| hsa-miR-4284 | -0,198149168 | -0,031836225 | -0,3580359 | -0,196 | 0,163 |
| hsa-miR-640 | -0,127466639 | -0,252590651 | -0,20569873 | -0,195 | 0,063 |
| hsa-miR-23a* | 0,079686264 | -0,414345856 | -0,24771863 | -0,194 | 0,251 |
| hsa-miR-551b* | -0,111306335 | -1,376460833 | -0,27681894 | -0,588 | 0,688 |
| hsa-miR-103a-2* | -0,185790067 | -0,13825178 | -0,25730784 | -0,194 | 0,060 |
| hsa-miR-1249 | -0,341714247 | -0,125625329 | -0,11154985 | -0,193 | 0,129 |
| hsa-miR-664* | -0,041126541 | -0,343054022 |  | -0,192 | 0,213 |
| hsa-miR-138 | -0,246502304 | -0,194146395 | -0,13122425 | -0,191 | 0,058 |
| hsa-miR-3134 | -0,181691982 | -0,145260592 | -0,24405108 | -0,190 | 0,050 |
| hsa-miR-4277 | -0,20468331 | -0,118719073 | -0,24695337 | -0,190 | 0,065 |
| hsa-miR-4522 | -0,282185532 | -0,084889927 | -0,203033 | -0,190 | 0,099 |
| hsa-miR-1228* | -0,017048407 | -0,389121786 | -0,16056543 | -0,189 | 0,188 |
| hsa-miR-1266 | -0,463591924 | -0,07301247 | -0,02952223 | -0,189 | 0,239 |
| hsa-miR-1288 | -0,178719838 | -0,147180236 | -0,24003664 | -0,189 | 0,047 |
| hsa-miR-4423-5p | -0,058697679 | -0,313996386 | -0,19287701 | -0,189 | 0,128 |
| hsa-miR-3612 | -0,091862238 | -0,340870088 | -0,13045037 | -0,188 | 0,134 |
| hsa-miR-190 | -0,164441417 | -0,167871149 | -0,2307658 | -0,188 | 0,037 |
| hsa-miR-7-1* | -0,095654884 | -0,179718336 | -0,28740114 | -0,188 | 0,096 |
| hsa-miR-1293 | -0,298297612 | 0,015654073 | -0,27837921 | -0,187 | 0,176 |
| hsa-miR-18a* | -0,159709228 | -1,210165773 | -0,21383432 | -0,528 | 0,591 |
| hsa-miR-3684 | -0,018476082 | -0,168127204 | -0,37364518 | -0,187 | 0,178 |
| hsa-miR-4674 | 0,084124468 | -0,06125576 | -0,58272153 | -0,187 | 0,351 |
| hsa-miR-345 | -0,12131129 | -0,156391114 | -0,27923593 | -0,186 | 0,083 |
| hsa-miR-4736 | -0,11906009 | -0,186768403 | -0,24847097 | -0,185 | 0,065 |
| hsa-miR-1260b | -0,142786511 | -0,151620223 | -0,25889004 | -0,184 | 0,065 |
| hsa-miR-3180 | -0,077661149 | -0,207090553 | -0,26779832 | -0,184 | 0,097 |
| hsa-miR-20b* | -0,273296315 | 0,239207482 | -0,51668833 | -0,184 | 0,386 |
| hsa-miR-4732-5p | -0,07937666 | -0,232785516 | -0,23707528 | -0,183 | 0,090 |
| hsa-miR-195 | -0,142025888 | -0,383591934 | -0,02102001 | -0,182 | 0,185 |
| hsa-miR-564 | -0,094006123 | -0,140552018 | -0,31179822 | -0,182 | 0,115 |
| hsa-let-7c | -0,239975821 | -0,268191517 | -0,03789843 | -0,182 | 0,126 |
| hsa-miR-608 | -0,142537601 | -0,105715469 | -0,2976001 | -0,182 | 0,102 |
| hsa-miR-9* | -0,049797885 | -0,29948087 | -0,19612659 | -0,182 | 0,125 |
| hsa-miR-629* | -0,094150419 | -0,267100104 |  | -0,181 | 0,122 |
| hsa-miR-1911* | -0,337846812 | -0,022944189 |  | -0,180 | 0,223 |
| hsa-miR-4515 | -0,029270084 | -0,232450344 | -0,27841326 | -0,180 | 0,133 |
| hsa-miR-192 | -0,203439096 | -0,328135524 | -0,00783464 | -0,180 | 0,161 |
| hsa-miR-379 | -0,409467697 | -0,200683735 | 0,07212914 | -0,179 | 0,242 |
| hsa-miR-1205 | -0,233488207 | -0,254393451 | -0,04988914 | -0,179 | 0,113 |
| hsa-miR-493 | -0,1283214 | -0,2058232 | -0,2012029 | -0,178 | 0,043 |
| hsa-miR-4670-5p | -0,392978425 | -0,128802234 | -0,01048735 | -0,177 | 0,196 |
| hsa-miR-517* | 0,025989401 | -0,405880869 | -0,15118408 | -0,177 | 0,217 |
| hsa-miR-3646 | -0,178137446 | -0,013475783 | -0,3390244 | -0,177 | 0,163 |
| hsa-miR-4738-3p | -0,144519258 | -0,063964264 | -0,3217567 | -0,177 | 0,132 |
| hsa-miR-3690 | -0,1957632 | -0,334527057 | 0,00045428 | -0,177 | 0,168 |
| hsa-miR-493* | -0,19936363 | -0,887857369 | -0,15291225 | -0,413 | 0,412 |
| hsa-miR-422a | -0,029433429 | -0,417158966 | -0,08142661 | -0,176 | 0,210 |
| hsa-miR-1287 | -0,09069086 | -0,21291628 | -0,21955269 | -0,174 | 0,073 |
| hsa-miR-4703-3p | -0,174269558 | -0,298926737 | -0,04941231 | -0,174 | 0,125 |
| hsa-miR-105* | -0,072366506 | -0,813272459 | -0,27486281 | -0,387 | 0,383 |
| hsa-miR-376a* | -0,140278465 | -0,03271299 | -0,34411104 | -0,172 | 0,158 |
| hsa-miR-4678 | -0,118965771 | -0,286011175 | -0,11197636 | -0,172 | 0,099 |
| hsa-miR-4756-5p | -0,119716627 | -0,330460734 | -0,06543506 | -0,172 | 0,140 |
| hsa-miR-376a | -0,139095386 | -0,266166236 | -0,10851904 | -0,171 | 0,084 |
| hsa-miR-140-3p | -0,17100646 | -0,109312506 | -0,23320529 | -0,171 | 0,062 |
| hsa-miR-26a-2* | 0,094159891 | -0,427463974 | -0,17999526 | -0,171 | 0,261 |
| hsa-miR-665 | -0,086323474 | -0,245307992 | -0,17970076 | -0,170 | 0,080 |
| hsa-miR-182* | -0,166365775 | -1,060814662 | -0,17446165 | -0,467 | 0,514 |
| hsa-miR-190b | -0,23833552 | -0,002598782 | -0,26980369 | -0,170 | 0,146 |
| hsa-miR-4536 | 0,035282517 | -0,347014889 | -0,19733325 | -0,170 | 0,193 |
| hsa-miR-4712-3p | -0,164247018 | -0,292655197 | -0,05191359 | -0,170 | 0,120 |
| hsa-miR-518c* | -0,154237186 | -0,184933978 |  | -0,170 | 0,022 |
| hsa-miR-4529-3p | -0,01627072 | -0,220447218 | -0,27160721 | -0,169 | 0,135 |
| hsa-miR-4733-3p | -0,098584727 | -0,156300732 | -0,25149508 | -0,169 | 0,077 |
| hsa-miR-27a* | -0,074776875 | -0,199844225 | -0,2316637 | -0,169 | 0,083 |
| hsa-miR-4675 | -0,142976048 | -0,111476717 | -0,24909251 | -0,168 | 0,072 |
| hsa-miR-4780 | -0,060695506 | -0,323746984 | -0,11822937 | -0,168 | 0,138 |
| hsa-miR-4684-3p | -0,395668384 | -0,040364596 | -0,06642001 | -0,167 | 0,198 |
| hsa-miR-4789-5p | -0,083263685 | -0,323740945 | -0,09261916 | -0,167 | 0,136 |
| hsa-let-7i* | -0,107665991 | -0,490936361 | 0,09931104 | -0,166 | 0,299 |
| hsa-miR-34a | -0,223160843 | -0,159251641 | -0,11468829 | -0,166 | 0,055 |
| hsa-miR-365* | -0,332088927 | 0,000896313 |  | -0,166 | 0,235 |
| hsa-miR-1255b | -0,101232411 | -0,162950703 | -0,23022506 | -0,165 | 0,065 |
| hsa-let-7d | -0,138806228 | -0,181476759 | -0,17246816 | -0,164 | 0,022 |
| hsa-miR-342-3p | -0,195164899 | -0,16053957 | -0,13591795 | -0,164 | 0,030 |
| hsa-miR-4688 | -0,085350853 | -0,145296987 | -0,26061632 | -0,164 | 0,089 |
| hsa-miR-15a* | -0,305259318 | -0,020616827 |  | -0,163 | 0,201 |
| hsa-miR-3125 | -0,052206611 | -0,201520663 | -0,23458502 | -0,163 | 0,097 |
| hsa-miR-4735-5p | -0,282579571 | -0,020593527 | -0,18493969 | -0,163 | 0,132 |
| hsa-miR-4670-3p | -0,18332751 | -0,104903635 | -0,1989364 | -0,162 | 0,050 |
| hsa-miR-224 | -0,00378891 | -0,091397697 | -0,39186 | -0,162 | 0,204 |
| hsa-let-7f | -0,157125628 | -0,125818619 | -0,20255878 | -0,162 | 0,039 |
| hsa-miR-5095 | -0,164861198 |  | -0,15844299 | -0,162 | 0,005 |
| hsa-miR-4473 | -0,120638252 | -0,041449566 | -0,32196986 | -0,161 | 0,145 |
| hsa-miR-4664-5p | -0,094383007 | -0,175541727 | -0,21184055 | -0,161 | 0,060 |
| hsa-miR-2278 | -0,008671747 | -0,21875747 | -0,25311458 | -0,160 | 0,132 |
| hsa-miR-764 | -0,10470659 | -0,100819594 | -0,27485101 | -0,160 | 0,099 |
| hsa-miR-3607-5p | -0,193678079 | -0,120895015 | -0,16579029 | -0,160 | 0,037 |
| hsa-miR-451 | -0,113947248 | -0,364124888 | -0,00220244 | -0,160 | 0,185 |
| hsa-miR-378b | -0,124644521 | -0,550072911 | 0,19521257 | -0,160 | 0,374 |
| hsa-miR-4723-3p | -0,046916754 | -0,334525492 | -0,09768424 | -0,160 | 0,154 |
| hsa-miR-3611 | -0,10492736 | -0,206632407 | -0,16682225 | -0,159 | 0,051 |
| hsa-miR-4326 | -0,053422674 | -0,254114247 | -0,16748262 | -0,158 | 0,101 |
| hsa-miR-4645-3p | 0,023904694 | 0,94 | -0,33962304 | 0,208 | 0,659 |
| hsa-miR-15b | -0,117064561 | -0,05307279 | -0,30335041 | -0,158 | 0,130 |
| hsa-miR-4633-5p | -0,101767076 | -0,196331084 | -0,17534789 | -0,158 | 0,050 |
| hsa-miR-4539 | -0,148632346 | -0,162937324 | -0,16024481 | -0,157 | 0,008 |
| hsa-miR-2116* | 0,039159058 | -0,444624361 | -0,06560667 | -0,157 | 0,255 |
| hsa-miR-4683 | 0,001805196 | -0,174821019 | -0,29755824 | -0,157 | 0,150 |
| hsa-miR-643 | -0,130971002 | -0,220861197 | -0,1178583 | -0,157 | 0,056 |
| hsa-let-7a | -0,201918436 | -0,185815073 | -0,0810222 | -0,156 | 0,066 |
| hsa-miR-1227 | -0,044550133 | -0,151970109 | -0,27135344 | -0,156 | 0,113 |
| hsa-miR-152 | -0,08901209 | -0,068772174 | -0,30801668 | -0,155 | 0,133 |
| hsa-miR-3681 | -0,137441056 | -0,059432576 | -0,26660115 | -0,154 | 0,105 |
| hsa-miR-4432 | -0,085170899 | -0,212676396 | -0,16401203 | -0,154 | 0,064 |
| hsa-miR-369-5p | -0,247445831 | -0,207347085 | -0,00659712 | -0,154 | 0,129 |
| hsa-miR-323b-5p | -0,38700363 | -0,031029237 | -0,03901783 | -0,152 | 0,203 |
| hsa-miR-4430 | -0,01421144 | -0,13623025 | -0,30149481 | -0,151 | 0,144 |
| hsa-miR-769-5p | -0,119529797 | -0,181537835 | -0,15045598 | -0,151 | 0,031 |
| hsa-miR-3150b-5p | -0,109459584 | 0,94 | -0,19038354 | 0,213 | 0,631 |
| hsa-miR-339-5p | -0,172250434 | -0,117395557 | -0,15962542 | -0,150 | 0,029 |
| hsa-miR-767-5p | -0,035715757 | -0,273185734 | -0,13886011 | -0,149 | 0,119 |
| hsa-miR-4512 | -0,129758952 | -0,44075105 | -0,16857799 | -0,246 | 0,169 |
| hsa-miR-4428 | -0,083323003 | -0,166294265 | -0,19503529 | -0,148 | 0,058 |
| hsa-miR-4652-5p | -0,191743137 | 0,95 | -0,10392327 | 0,218 | 0,635 |
| hsa-miR-548al | -0,072029913 | -0,210515569 | -0,16033113 | -0,148 | 0,070 |
| hsa-miR-4800-3p | -0,181148426 | -0,213405318 | -0,04444896 | -0,146 | 0,090 |
| hsa-miR-663b | -0,101282964 | -0,096449556 | -0,24113243 | -0,146 | 0,082 |
| hsa-miR-4501 | 0,032428967 | -0,192119509 | -0,27911633 | -0,146 | 0,161 |
| hsa-miR-26a-1* | 0,037685546 | -0,420716061 | -0,05459859 | -0,146 | 0,242 |
| hsa-miR-4417 | 0,050041073 | -0,244889143 | -0,23894541 | -0,145 | 0,169 |
| hsa-miR-611 | -0,052876716 | -0,33778294 | -0,04083289 | -0,144 | 0,168 |
| hsa-miR-4488 | -0,098590908 | -0,262889215 | -0,06920558 | -0,144 | 0,104 |
| hsa-miR-3914 | 0,003799767 | -0,180487427 | -0,25396771 | -0,144 | 0,133 |
| hsa-miR-1233 | -0,052645351 | -0,233898469 | -0,14265636 | -0,143 | 0,091 |
| hsa-miR-4270 | -0,159899899 | -0,073246818 | -0,1937732 | -0,142 | 0,062 |
| hsa-miR-2117 | -0,043442038 | -0,177420462 | -0,2058526 | -0,142 | 0,087 |
| hsa-miR-1286 | -0,181466678 | -0,184528026 | -0,05807354 | -0,141 | 0,072 |
| hsa-miR-3619-5p | -0,137627542 | -0,130457178 | -0,15527415 | -0,141 | 0,013 |
| hsa-miR-4790-3p | -0,029177509 | -0,175101315 | -0,21869545 | -0,141 | 0,099 |
| hsa-miR-4301 | -0,335382789 | -0,020193627 | -0,06704424 | -0,141 | 0,170 |
| hsa-let-7d* | -0,073046993 | -0,142675581 | -0,20617859 | -0,141 | 0,067 |
| hsa-miR-708* | -0,035974466 | -0,290794139 | -0,09297681 | -0,140 | 0,134 |
| hsa-miR-1296 | -0,076488687 | -0,12910141 | -0,21378679 | -0,140 | 0,069 |
| hsa-miR-4770 | -0,066348032 | -0,125391807 | -0,22758334 | -0,140 | 0,082 |
| hsa-miR-4754 | -0,020257971 | -0,059150223 | -0,338996 | -0,139 | 0,174 |
| hsa-miR-378g | -0,164215823 | -0,106758659 | -0,14493553 | -0,139 | 0,029 |
| hsa-miR-374b* | 0,068782118 | -0,311250958 | -0,17285272 | -0,138 | 0,192 |
| hsa-miR-1250 | -0,061391283 | -0,076506839 | -0,27669296 | -0,138 | 0,120 |
| hsa-miR-378h | -0,157519325 | -0,143638801 | -0,11324943 | -0,138 | 0,023 |
| hsa-miR-3689a-5p // hsa-miR-3689b // hsa-miR-3689e | -0,048095443 | -0,102498734 | -0,26336457 | -0,138 | 0,112 |
| hsa-miR-558 | -0,034476752 | -0,228986763 | -0,14944325 | -0,138 | 0,098 |
| hsa-miR-146b-5p | -0,084948165 | -0,043626416 | -0,28259357 | -0,137 | 0,128 |
| hsa-miR-765 | -0,016277177 | -0,195427564 | -0,19912614 | -0,137 | 0,105 |
| hsa-miR-196b* | -0,008899161 | -0,227377924 | -0,17258014 | -0,136 | 0,114 |
| hsa-miR-4692 | -0,140532997 | -0,107689574 | -0,15955377 | -0,136 | 0,026 |
| hsa-miR-3675-5p | 0,058557928 | -0,220345158 | -0,2455363 | -0,136 | 0,169 |
| hsa-miR-3074-3p | -0,081915318 | -0,000671608 | -0,32004748 | -0,134 | 0,166 |
| hsa-miR-4519 | -0,108845759 | -0,117779461 | -0,17438307 | -0,134 | 0,036 |
| hsa-miR-98 | -0,118934212 | -0,08975869 | -0,19168669 | -0,133 | 0,052 |
| hsa-miR-1178 | -0,092006582 | -0,116639324 | -0,19108514 | -0,133 | 0,052 |
| hsa-miR-597 | 0,022758404 | -0,209130941 | -0,21240392 | -0,133 | 0,135 |
| hsa-miR-4314 | -0,035569407 | -0,152667389 | -0,21003102 | -0,133 | 0,089 |
| hsa-miR-299-5p | -0,14514745 | -0,231520249 | -0,01927533 | -0,132 | 0,107 |
| hsa-miR-22* | -0,0276249 | -0,321789921 | -0,04618368 | -0,132 | 0,165 |
| hsa-miR-4659a-5p | 0,016356205 | 0,9 | -0,27969154 | 0,212 | 0,614 |
| hsa-miR-516b* // hsa-miR-516a-3p | -0,336340135 | -0,036745457 | -0,01781057 | -0,130 | 0,179 |
| hsa-miR-541 | -0,246733445 | -0,095692969 | -0,04812645 | -0,130 | 0,104 |
| hsa-miR-127-3p | -0,283319689 | -0,063761741 | -0,04296015 | -0,130 | 0,133 |
| hsa-let-7g* | -0,10640843 | -0,19589378 | -0,08710373 | -0,130 | 0,058 |
| hsa-miR-424* | -0,436225213 | 0,17787093 |  | -0,129 | 0,434 |
| hsa-miR-4643 | -0,140875145 | -0,190523417 | -0,05145359 | -0,128 | 0,070 |
| hsa-miR-183 | -0,12312728 | -0,331572557 | 0,07237685 | -0,127 | 0,202 |
| hsa-miR-4656 | -0,058443275 | -0,142156556 | -0,18079459 | -0,127 | 0,063 |
| hsa-miR-296-5p | -0,141012324 | -0,211142834 | -0,02828002 | -0,127 | 0,092 |
| hsa-miR-125a-5p | -0,252562182 | -0,105761007 | -0,02181096 | -0,127 | 0,117 |
| hsa-miR-367* | -0,203542006 | -0,049680547 |  | -0,127 | 0,109 |
| hsa-miR-222 | -0,049628247 | -0,129011436 | -0,20023339 | -0,126 | 0,075 |
| hsa-miR-3669 | -0,01073493 | -0,131910787 | -0,23317108 | -0,125 | 0,111 |
| hsa-miR-4706 | -0,063395638 | 0,95 | -0,18637896 | 0,233 | 0,624 |
| hsa-miR-4664-3p | 0,023230509 | -0,139621545 | -0,25815168 | -0,125 | 0,141 |
| hsa-miR-589 | 0,039807878 | -0,230085181 | -0,18350597 | -0,125 | 0,144 |
| hsa-miR-4726-5p | -0,034685135 | -0,104293599 | -0,23247967 | -0,124 | 0,100 |
| hsa-miR-361-5p | -0,137666244 | -0,196375544 | -0,03360134 | -0,123 | 0,082 |
| hsa-miR-380* | -0,019433147 | -1,934013159 | -0,22510659 | -0,726 | 1,051 |
| hsa-miR-885-5p | -0,077960235 | -0,167398391 | -0,12126966 | -0,122 | 0,045 |
| hsa-miR-1224-5p | -0,031979601 | -0,190993316 | -0,14334717 | -0,122 | 0,082 |
| hsa-miR-499-5p | -0,18300252 | -0,139373945 | -0,04307651 | -0,122 | 0,072 |
| hsa-miR-95 | -0,212763689 | -0,066423195 | -0,08604076 | -0,122 | 0,079 |
| hsa-miR-21* | 0,040758532 | -0,405722842 | 0,00398892 | -0,120 | 0,248 |
| hsa-miR-105 | -0,016328148 | -0,09737239 | -0,24727465 | -0,120 | 0,117 |
| hsa-miR-185* | -0,153947208 | -0,141219209 | -0,06347552 | -0,120 | 0,049 |
| hsa-miR-1283 | 0,019759993 | -0,168820975 | -0,2073476 | -0,119 | 0,122 |
| hsa-miR-454* | -0,068756084 | -0,961034234 | -0,16765528 | -0,399 | 0,489 |
| hsa-miR-3924 | -0,015984201 | -0,260754797 | -0,07548397 | -0,117 | 0,128 |
| hsa-miR-1262 | -0,054141022 | -0,168746553 | -0,12856106 | -0,117 | 0,058 |
| hsa-miR-936 | -0,055179828 | -0,246668849 | -0,04742933 | -0,116 | 0,113 |
| hsa-miR-1246 | -0,082674951 | -0,100274478 | -0,16219674 | -0,115 | 0,042 |
| hsa-miR-513b | -0,052257728 | -0,073965141 | -0,21787506 | -0,115 | 0,090 |
| hsa-miR-29a* | 0,052614579 | -0,253138011 | -0,14262594 | -0,114 | 0,155 |
| hsa-miR-933 | 0,006255031 | -0,140361557 | -0,20837599 | -0,114 | 0,110 |
| hsa-miR-4475 | -0,008408672 | -0,139458192 | -0,19335113 | -0,114 | 0,095 |
| hsa-miR-4650-3p | -0,049899072 | -0,200882661 | -0,09012762 | -0,114 | 0,078 |
| hsa-miR-3150b-3p | -0,081466656 | -0,118808721 | -0,13793628 | -0,113 | 0,029 |
| hsa-miR-33a | -0,182340918 | -0,110062203 | -0,04535409 | -0,113 | 0,069 |
| hsa-miR-409-5p | -0,188854552 | 0,005914412 | -0,1518198 | -0,112 | 0,103 |
| hsa-miR-4782-5p | -0,111511738 | -0,125062693 | -0,09592526 | -0,111 | 0,015 |
| hsa-miR-2682* | 0,169940692 | -0,391198256 |  | -0,111 | 0,397 |
| hsa-miR-7 | -0,233668455 | -0,07327575 | -0,02287853 | -0,110 | 0,110 |
| hsa-miR-320e | -0,036911794 | -0,159104876 | -0,13351747 | -0,110 | 0,064 |
| hsa-miR-186* | 0,034473903 | 0,875938629 | -0,2526111 | 0,219 | 0,587 |
| hsa-miR-4311 | -0,058997355 | -0,186602888 | -0,08120764 | -0,109 | 0,068 |
| hsa-miR-3616-5p | -0,015928531 | 0,99 | -0,20188498 | 0,257 | 0,641 |
| hsa-miR-1302 | -0,159677874 | 0,098649835 | -0,26479519 | -0,109 | 0,187 |
| hsa-miR-509-3p | -0,173432526 | -0,344432466 | 0,19234243 | -0,109 | 0,274 |
| hsa-let-7f-2* | -0,193687405 | -0,021974416 |  | -0,108 | 0,121 |
| hsa-miR-4730 | -0,098799674 | -0,153927398 | -0,07014738 | -0,108 | 0,043 |
| hsa-miR-3155 | 0,074976534 | -0,292194703 | -0,10518332 | -0,107 | 0,184 |
| hsa-miR-4774-3p | -0,191162462 | -0,136050098 | 0,00556285 | -0,107 | 0,101 |
| hsa-miR-3672 | -0,101819192 | 0,99 | -0,11222181 | 0,259 | 0,633 |
| hsa-miR-4485 | 0,041758844 | -0,294155447 | -0,06858928 | -0,107 | 0,171 |
| hsa-miR-4446-3p | -0,082924608 | 0,96 | -0,13097185 | 0,249 | 0,616 |
| hsa-miR-1207-3p | 0,00315319 | -0,156274834 | -0,16758383 | -0,107 | 0,095 |
| hsa-miR-4327 | -0,090741484 | -0,239712367 | 0,00982325 | -0,107 | 0,126 |
| hsa-miR-637 | 0,004978561 | -0,108317503 | -0,21641049 | -0,107 | 0,111 |
| hsa-miR-150* | -0,03847946 | 1,564160505 | -0,17442414 | 0,450 | 0,967 |
| hsa-miR-125b-1* | 0,078445244 | -0,245473811 | -0,15215989 | -0,106 | 0,167 |
| hsa-miR-4727-3p | -0,196747166 | -0,031516814 | -0,09090778 | -0,106 | 0,084 |
| hsa-miR-3164 | -0,017414198 | -0,060964657 | -0,24011398 | -0,106 | 0,118 |
| hsa-miR-1226* | -0,079210231 | 0,145668825 | -0,38404075 | -0,106 | 0,266 |
| hsa-miR-513a-5p | 0,044244341 | -0,166407084 | -0,19524265 | -0,106 | 0,131 |
| hsa-miR-4684-5p | 0,037574239 | -0,249468802 | -0,10444472 | -0,105 | 0,144 |
| hsa-miR-335* | -0,078803947 | -0,229410105 | -0,0064832 | -0,105 | 0,114 |
| hsa-miR-4274 | -0,018829299 | 0,94 | -0,19092501 | 0,243 | 0,609 |
| hsa-miR-4717-3p | -0,254183689 | -0,03345278 | -0,02588179 | -0,105 | 0,130 |
| hsa-miR-491-5p | -0,216553401 | -0,002421418 | -0,09380421 | -0,104 | 0,107 |
| hsa-miR-664 | -0,081886894 | -0,171362118 | -0,05867345 | -0,104 | 0,060 |
| hsa-miR-30c-2* | -0,00665138 | -0,195416519 | -0,10927638 | -0,104 | 0,095 |
| hsa-miR-499a-3p | 0,033304879 | -0,176065433 | -0,16644679 | -0,103 | 0,118 |
| hsa-miR-525-3p | -0,109927367 | -0,189586705 | -0,00919481 | -0,103 | 0,090 |
| hsa-miR-3605-3p | -0,050454794 | -0,116831516 | -0,13996738 | -0,102 | 0,046 |
| hsa-miR-4317 | -0,074919604 | -0,213046012 | -0,0176613 | -0,102 | 0,100 |
| hsa-miR-4662b | -0,008655621 | -0,087202549 | -0,20951513 | -0,102 | 0,101 |
| hsa-miR-3685 | -0,034688902 | -0,109988693 | -0,15815603 | -0,101 | 0,062 |
| hsa-miR-30c-1* | 0,038109565 | -0,213330662 | -0,12750911 | -0,101 | 0,128 |
| hsa-miR-519c-5p // hsa-miR-519b-5p // hsa-miR-523* // hsa-miR-518e* // hsa-miR-522* // hsa-miR-519a* | -0,009827008 | -0,078923798 | -0,21367311 | -0,101 | 0,104 |
| hsa-miR-3908 | -0,02284891 | -0,181568017 | -0,09658511 | -0,100 | 0,079 |
| hsa-miR-4662a-5p | -0,066827301 | -0,018412858 | -0,21500839 | -0,100 | 0,102 |
| hsa-miR-188-5p | -0,132826678 | -0,055609868 | -0,11087842 | -0,100 | 0,040 |
| hsa-miR-3122 | -0,209574412 | -0,012617086 | -0,0768647 | -0,100 | 0,100 |
| hsa-miR-2110 | -0,131058256 | -0,099775164 | -0,06780834 | -0,100 | 0,032 |
| hsa-miR-3185 | -0,036883275 | 0,028852908 | -0,29028206 | -0,099 | 0,169 |
| hsa-miR-4666-5p | -0,08029971 | -0,047178615 | -0,17080925 | -0,099 | 0,064 |
| hsa-miR-625* | -0,207602795 | 0,008981143 |  | -0,099 | 0,153 |
| hsa-miR-455-5p | -0,17289207 | -0,067399757 | -0,05669115 | -0,099 | 0,064 |
| hsa-miR-3160-3p | -0,14271638 | 0,045919397 | -0,1999432 | -0,099 | 0,129 |
| hsa-miR-503 | -0,050733513 | -0,096643351 | -0,1481602 | -0,099 | 0,049 |
| hsa-miR-423-3p | -0,270558481 | -0,022278959 | -0,00257712 | -0,098 | 0,149 |
| hsa-miR-629 | -0,410284733 | -0,116787155 | 0,23186397 | -0,098 | 0,321 |
| hsa-miR-526b | -0,018775187 | -0,093800169 | -0,18215441 | -0,098 | 0,082 |
| hsa-miR-33a* | -0,138955268 | -0,661592564 | -0,0571966 | -0,286 | 0,328 |
| hsa-miR-135a | -0,101196363 | -0,18872287 | -0,00412869 | -0,098 | 0,092 |
| hsa-miR-377* | -0,061314801 | -0,16920705 | -0,0629091 | -0,098 | 0,062 |
| hsa-miR-4695-5p | -0,049205526 | -0,069737035 | -0,17346816 | -0,097 | 0,067 |
| hsa-miR-2113 | -0,076093329 | 1 | -0,11704659 | 0,269 | 0,633 |
| hsa-miR-147 | -0,187423946 | 0,008492525 | -0,10833081 | -0,096 | 0,099 |
| hsa-miR-124 | -0,091451933 | -0,161347024 | -0,03282138 | -0,095 | 0,064 |
| hsa-miR-3941 | -0,002778665 | -0,029305133 | -0,25348803 | -0,095 | 0,138 |
| hsa-miR-606 | -0,02277777 | -0,181410129 | -0,08097105 | -0,095 | 0,080 |
| hsa-miR-218-2* | -0,038929848 | -0,15975132 | -0,08611181 | -0,095 | 0,061 |
| hsa-let-7e* | 0,082418169 | -0,203141185 | -0,16331629 | -0,095 | 0,155 |
| hsa-miR-2909 | -0,110417752 | -0,053261786 | -0,1189425 | -0,094 | 0,036 |
| hsa-miR-134 | -0,05343249 | -0,048374885 | -0,17967366 | -0,094 | 0,074 |
| hsa-miR-4323 | -0,08814868 | 1,1 | -0,09898626 | 0,304 | 0,689 |
| hsa-miR-4300 | -0,085232186 | -0,109035997 | -0,08605357 | -0,093 | 0,014 |
| hsa-miR-15a | -0,110532889 | -0,001881816 | -0,16757952 | -0,093 | 0,084 |
| hsa-miR-3136-5p | 0,05484239 | -0,217384266 | -0,11661952 | -0,093 | 0,138 |
| hsa-miR-1322 | 0,042181562 | -0,143291354 | -0,17734929 | -0,093 | 0,118 |
| hsa-miR-1269 | -0,048484466 | -0,102227825 | -0,12461093 | -0,092 | 0,039 |
| hsa-miR-2467-5p | -0,020354174 | -0,243882375 | -0,01107864 | -0,092 | 0,132 |
| hsa-miR-4708-5p | -0,072663589 | -0,212157406 | 0,01012426 | -0,092 | 0,112 |
| hsa-miR-10a* | -0,007301219 | -0,176470965 | -0,09016696 | -0,091 | 0,085 |
| hsa-miR-92a-1* | -0,190715221 | 0,008600203 |  | -0,091 | 0,141 |
| hsa-miR-1253 | -0,011871984 | 0,001582917 | -0,26198426 | -0,091 | 0,148 |
| hsa-miR-758 | -0,118188807 | -0,036612987 | -0,11666659 | -0,090 | 0,047 |
| hsa-miR-4719 | 0,001460354 | -0,217825458 | -0,05432606 | -0,090 | 0,114 |
| hsa-miR-214* | -0,006860129 | -0,132377355 | -0,13019797 | -0,090 | 0,072 |
| hsa-miR-4308 | 0,050798681 | -0,004912335 | -0,31355967 | -0,089 | 0,196 |
| hsa-miR-15b* | 0,091703751 | -0,241358134 | -0,11750758 | -0,089 | 0,168 |
| hsa-miR-1321 | 0,085695854 | -0,281906057 | -0,07092099 | -0,089 | 0,184 |
| hsa-miR-2681* | -0,029892923 | -0,841778364 | -0,14799452 | -0,340 | 0,439 |
| hsa-miR-4740-5p | -0,077361976 | -0,143913942 | -0,04500367 | -0,089 | 0,050 |
| hsa-miR-3142 | -0,039619872 | -0,03315373 | -0,19303699 | -0,089 | 0,090 |
| hsa-miR-153 | 0,0374058 | -0,160717857 | -0,14141947 | -0,088 | 0,109 |
| hsa-miR-4742-5p | -0,077780183 | -0,094525066 | -0,09088267 | -0,088 | 0,009 |
| hsa-miR-516b | -0,025529232 | -0,207906706 | -0,02850436 | -0,087 | 0,104 |
| hsa-miR-1275 | 0,089809336 | -0,236691013 | -0,11459317 | -0,087 | 0,165 |
| hsa-miR-3127-5p | -0,096986016 | -0,060715761 | -0,10289325 | -0,087 | 0,023 |
| hsa-miR-3664-3p | -0,38407052 | -0,01825842 | 0,14219029 | -0,087 | 0,270 |
| hsa-miR-610 | 0,116849693 | -0,22618673 | -0,15061554 | -0,087 | 0,180 |
| hsa-miR-340* | -0,041888414 | -0,125589116 | -0,09135142 | -0,086 | 0,042 |
| hsa-miR-3683 | -0,039845786 | -0,00820327 | -0,21070481 | -0,086 | 0,109 |
| hsa-miR-1260 | -0,022743631 | -0,057493456 | -0,17827476 | -0,086 | 0,082 |
| hsa-miR-3977 | 0,004744429 | -0,094025453 | -0,16749618 | -0,086 | 0,086 |
| hsa-miR-4761-5p | -0,092000793 | 0,008582861 | -0,17219351 | -0,085 | 0,091 |
| hsa-miR-485-5p | -0,162226538 | -0,030620471 | -0,06005117 | -0,084 | 0,069 |
| hsa-miR-3616-3p | -0,118505291 | -0,014245814 | -0,11914035 | -0,084 | 0,060 |
| hsa-miR-4511 | 0,022556522 | -0,042802776 | -0,22926436 | -0,083 | 0,131 |
| hsa-miR-4312 | -0,114656644 | 0,074739363 | -0,20936403 | -0,083 | 0,145 |
| hsa-miR-4654 | -0,171748759 | -0,162868155 | 0,08577832 | -0,083 | 0,146 |
| hsa-miR-329 | 0,067543007 | -0,176379871 | -0,13869786 | -0,083 | 0,131 |
| hsa-miR-4306 | -0,035591799 | -0,080776072 | -0,13073149 | -0,082 | 0,048 |
| hsa-miR-4723-5p | -0,054067451 | -0,074088986 | -0,11878686 | -0,082 | 0,033 |
| hsa-miR-1236 | 0,038949476 | -0,163789985 | -0,12171556 | -0,082 | 0,107 |
| hsa-miR-212 | -0,083871457 | -0,151685784 | -0,01092407 | -0,082 | 0,070 |
| hsa-miR-323b-3p | -0,289180887 | -0,142592271 | 0,18618024 | -0,082 | 0,243 |
| hsa-miR-106b* | -0,193104467 | -0,002263327 | -0,04876892 | -0,081 | 0,100 |
| hsa-miR-424 | -0,051169686 | -0,040170243 | -0,15111284 | -0,081 | 0,061 |
| hsa-miR-761 | -0,054909335 | -0,012911094 | -0,17455498 | -0,081 | 0,084 |
| hsa-miR-1197 | -0,118582265 | 0,028101881 | -0,15169512 | -0,081 | 0,096 |
| hsa-miR-3141 | -0,042897047 | -0,104380165 | -0,09374932 | -0,080 | 0,033 |
| hsa-miR-663 | -0,042229848 | 0,93 | -0,11741099 | 0,257 | 0,584 |
| hsa-miR-4523 | 0,07401898 | -0,052198024 | -0,26089767 | -0,080 | 0,169 |
| hsa-miR-3688-5p | 0,04228254 | -0,099940672 | -0,18113916 | -0,080 | 0,113 |
| hsa-miR-202* | 0,027531394 | -0,88776065 | -0,18604387 | -0,349 | 0,479 |
| hsa-miR-4752 | -0,022281278 | -0,187457177 | -0,02794004 | -0,079 | 0,094 |
| hsa-miR-3180-3p | 0,002628194 | -0,117955945 | -0,121868 | -0,079 | 0,071 |
| hsa-miR-4500 | 0,006018824 | -0,09371258 | -0,14946398 | -0,079 | 0,079 |
| hsa-miR-32* | 0,011701477 | -1,462506386 | -0,16974739 | -0,540 | 0,804 |
| hsa-miR-4796-3p | -0,026094473 | -0,108401312 | -0,10248078 | -0,079 | 0,046 |
| hsa-miR-767-3p | -0,215254815 | 0,088860275 | -0,10993332 | -0,079 | 0,154 |
| hsa-miR-4646-3p | -0,042063142 | 0,021777373 | -0,2153901 | -0,079 | 0,123 |
| hsa-miR-410 | -0,077366825 | -0,27448365 | 0,11618632 | -0,079 | 0,195 |
| hsa-miR-3133 | 0,05041774 | -0,139838566 | -0,14589202 | -0,078 | 0,112 |
| hsa-miR-297 | -0,200077362 | 0,025472599 | -0,05962838 | -0,078 | 0,114 |
| hsa-miR-628-5p | -0,001646533 | -0,19700988 | -0,03541755 | -0,078 | 0,104 |
| hsa-miR-3714 | -0,094828045 | 0,053124202 | -0,19115122 | -0,078 | 0,123 |
| hsa-miR-325 | 0,099133385 | -0,051332999 | -0,28065284 | -0,078 | 0,191 |
| hsa-miR-363* | -0,053719478 | -0,130842605 | -0,04726242 | -0,077 | 0,047 |
| hsa-miR-4786-5p | -0,056638848 | -0,051143127 | -0,12364501 | -0,077 | 0,040 |
| hsa-miR-3173-3p | -0,048821409 | 0,01311933 | -0,1944287 | -0,077 | 0,107 |
| hsa-miR-150 | -0,030617661 | -0,245727581 | 0,04687666 | -0,076 | 0,152 |
| hsa-miR-302d* | 0,071291681 | -0,25999442 | -0,0403571 | -0,076 | 0,169 |
| hsa-miR-4677-3p | -0,102039552 | -0,219735282 | 0,09273935 | -0,076 | 0,158 |
| hsa-miR-545* | 0,044437947 | 0,667532942 | -0,19620571 | 0,172 | 0,446 |
| hsa-miR-124* | 0,005673222 | -0,227185267 | -0,00606837 | -0,076 | 0,131 |
| hsa-miR-3157-5p | -0,058360441 | 0,014303719 | -0,18246611 | -0,076 | 0,099 |
| hsa-miR-676* | -0,008252812 | -0,235234932 | 0,01697769 | -0,076 | 0,139 |
| hsa-miR-135a* | -0,07831498 | -0,053382204 | -0,09475382 | -0,075 | 0,021 |
| hsa-miR-3658 | 0,0283501 | -0,047384278 | -0,20571353 | -0,075 | 0,119 |
| hsa-miR-1273e | 0,115419736 | -0,223972619 | -0,11499291 | -0,075 | 0,173 |
| hsa-miR-449b | -0,036203604 | -0,015872937 | -0,16983523 | -0,074 | 0,084 |
| hsa-miR-490-3p | -0,152771701 | -0,000394072 | -0,06853777 | -0,074 | 0,076 |
| hsa-miR-138-2* | -0,063231205 | -0,08454006 |  | -0,074 | 0,015 |
| hsa-miR-4759 | 0,04240701 | -0,0738165 | -0,18942441 | -0,074 | 0,116 |
| hsa-miR-507 | -0,024308636 | -0,162829409 | -0,0326794 | -0,073 | 0,078 |
| hsa-miR-103a | -0,182886894 | -0,147422066 | 0,11068015 | -0,073 | 0,160 |
| hsa-miR-195* | 0,044911271 | -0,093328185 | -0,17111386 | -0,073 | 0,109 |
| hsa-miR-302c* | 0,055709864 | -0,182717295 | -0,09245426 | -0,073 | 0,120 |
| hsa-miR-378* | 0,008086864 | -0,131033597 | -0,09382056 | -0,072 | 0,072 |
| hsa-miR-3157-3p | -0,01679337 | -0,046659071 | -0,15314994 | -0,072 | 0,072 |
| hsa-miR-634 | -0,049426223 | -0,08188336 | -0,08497437 | -0,072 | 0,020 |
| hsa-miR-873 | -0,089515931 | -0,168391392 | 0,04185679 | -0,072 | 0,106 |
| hsa-miR-130a* | 0,016854708 | 0,750311341 | -0,16068886 | 0,202 | 0,483 |
| hsa-miR-194* | -0,058658984 | -0,127594756 | -0,02785405 | -0,071 | 0,051 |
| hsa-miR-3159 | 0,041548057 | -0,148692569 | -0,10464095 | -0,071 | 0,100 |
| hsa-miR-4283 | 0,030878312 | -0,188277587 | -0,05305302 | -0,070 | 0,111 |
| hsa-miR-143 | -0,090937423 | -0,024634797 | -0,09449364 | -0,070 | 0,039 |
| hsa-miR-4257 | -0,031108114 | -0,089789804 | -0,08905379 | -0,070 | 0,034 |
| hsa-miR-4648 | -0,094090946 | -0,086079543 | -0,02841875 | -0,070 | 0,036 |
| hsa-miR-4639-5p | 0,049643976 | -0,036306745 | -0,2218374 | -0,070 | 0,139 |
| hsa-miR-3194-5p | -0,102422796 | 0,008662931 | -0,11466322 | -0,069 | 0,068 |
| hsa-miR-4662a-3p | 0,059193998 | -0,127277902 | -0,13976421 | -0,069 | 0,111 |
| hsa-miR-4724-5p | 0,01153633 | -0,0555633 | -0,16378439 | -0,069 | 0,088 |
| hsa-miR-155* | -0,041031471 | -0,097306392 |  | -0,069 | 0,040 |
| hsa-miR-365 | -0,111603083 | 0,005085301 | -0,10043244 | -0,069 | 0,064 |
| hsa-miR-3173-5p | -0,04328853 | -0,134848665 | -0,02860738 | -0,069 | 0,058 |
| hsa-miR-4433 | -0,002100854 | -0,247002822 | 0,04287089 | -0,069 | 0,156 |
| hsa-miR-135b | -0,067457242 | -0,053325189 | -0,0850543 | -0,069 | 0,016 |
| hsa-miR-3689f | 0,132113824 | -0,065527022 | -0,2723892 | -0,069 | 0,202 |
| hsa-miR-4533 | -0,020810814 | -0,08722979 | -0,09750817 | -0,069 | 0,042 |
| hsa-miR-125a-3p | -0,094900219 | -0,160071858 | 0,04951518 | -0,068 | 0,107 |
| hsa-miR-3940-5p | 0,063027604 | -0,224303637 | -0,04396524 | -0,068 | 0,145 |
| hsa-miR-4278 | -0,131200429 | -0,103786205 | 0,03024394 | -0,068 | 0,086 |
| hsa-miR-4731-5p | 0,039472654 | -0,013292753 | -0,22982187 | -0,068 | 0,143 |
| hsa-miR-4268 | 0,062143272 | -0,169487046 | -0,09624478 | -0,068 | 0,118 |
| hsa-miR-4424 | 0,019527065 | -0,219954498 | -0,00271258 | -0,068 | 0,132 |
| hsa-miR-499a-5p | 0,025885991 | -0,046412061 | -0,18211506 | -0,068 | 0,106 |
| hsa-miR-101* | 0,012078917 | -0,104815862 | -0,10989951 | -0,068 | 0,069 |
| hsa-miR-541* | -0,12856222 | -0,00608749 |  | -0,067 | 0,087 |
| hsa-miR-1292 | -0,08876195 | 0,007879375 | -0,11910846 | -0,067 | 0,066 |
| hsa-miR-1267 | -0,095291291 | -0,035351355 | -0,06879752 | -0,066 | 0,030 |
| hsa-miR-433 | 0,001257225 | -0,034274861 | -0,16616966 | -0,066 | 0,088 |
| hsa-miR-130b | -0,119090632 | -0,095987696 | 0,01622863 | -0,066 | 0,072 |
| hsa-miR-3166 | -0,200393544 | -0,051887008 | 0,05403664 | -0,066 | 0,128 |
| hsa-miR-3925-3p | 0,006629209 | -0,022996135 | -0,18150391 | -0,066 | 0,101 |
| hsa-miR-3139 | -0,013504415 | -0,141405721 | -0,0421909 | -0,066 | 0,067 |
| hsa-miR-641 | -0,091339891 | 0,119032694 | -0,22403022 | -0,065 | 0,173 |
| hsa-miR-4290 | 0,004223005 | -0,029154793 | -0,17064409 | -0,065 | 0,093 |
| hsa-miR-3201 | 0,009895586 | -0,040654439 | -0,16454677 | -0,065 | 0,090 |
| hsa-miR-30b | -0,086545839 | -0,076115688 | -0,03237355 | -0,065 | 0,029 |
| hsa-miR-646 | -0,162615791 | 0,97 | 0,03340751 | 0,280 | 0,605 |
| hsa-miR-185 | -0,04767125 | -0,010026709 | -0,13571859 | -0,064 | 0,065 |
| hsa-miR-486-5p | -0,137100455 | 0,009729093 | -0,06354025 | -0,064 | 0,073 |
| hsa-miR-4800-5p | 0,037288351 | -0,113932754 | -0,1142543 | -0,064 | 0,087 |
| hsa-miR-30c | -0,149877449 | -0,062057188 | 0,02117439 | -0,064 | 0,086 |
| hsa-miR-3944-3p | -0,165803104 | -0,01346392 | -0,01136062 | -0,064 | 0,089 |
| hsa-miR-1306 | -0,236357115 | -0,058287752 | 0,10477226 | -0,063 | 0,171 |
| hsa-miR-18b | 0,017170051 | -0,072912846 | -0,13374653 | -0,063 | 0,076 |
| hsa-miR-574-3p | -0,132095813 | 0,047701432 | -0,10364813 | -0,063 | 0,097 |
| hsa-miR-4742-3p | 0,005981201 | -0,112881748 | -0,08101271 | -0,063 | 0,062 |
| hsa-miR-1269b | 0,02582083 | -0,235390349 | 0,02186651 | -0,063 | 0,150 |
| hsa-miR-3943 | 0,012863879 | -0,210206103 | 0,00982747 | -0,063 | 0,128 |
| hsa-miR-4747-5p | 0,063641139 | -0,116280183 | -0,13455092 | -0,062 | 0,110 |
| hsa-miR-636 | 0,033170818 | 1 | -0,15784234 | 0,292 | 0,621 |
| hsa-miR-499-3p | -0,032065978 | 0,043601131 | -0,19725105 | -0,062 | 0,123 |
| hsa-miR-25* | -0,156923936 | 0,033172942 |  | -0,062 | 0,134 |
| hsa-miR-181c | -0,075687724 | -0,002810659 | -0,10685489 | -0,062 | 0,053 |
| hsa-miR-4324 | 0,096146306 | -0,097529407 | -0,18393837 | -0,062 | 0,143 |
| hsa-miR-28-5p | -0,115437092 | 0,054726742 | -0,12409637 | -0,062 | 0,101 |
| hsa-miR-4745-5p | 0,09559912 | -0,129818401 | -0,14753259 | -0,061 | 0,136 |
| hsa-miR-548ac | -0,037299251 | -0,034292345 | -0,10875701 | -0,060 | 0,042 |
| hsa-miR-877 | -0,015958478 | 0,022058261 | -0,18629094 | -0,060 | 0,111 |
| hsa-miR-548j | 0,018141038 | 0,96 | -0,13733104 | 0,280 | 0,594 |
| hsa-miR-3680 | 0,020005319 | 1,02 | -0,13917276 | 0,300 | 0,628 |
| hsa-miR-100* | 0,010084902 | -0,110634482 | -0,0766288 | -0,059 | 0,062 |
| hsa-miR-4494 | 0,120535332 | -0,218384485 | -0,07931169 | -0,059 | 0,170 |
| hsa-miR-143* | 0,00959702 | -0,127635719 |  | -0,059 | 0,097 |
| hsa-miR-3913-3p | 0,024600759 | -0,034263686 | -0,16708287 | -0,059 | 0,098 |
| hsa-miR-301a | -0,094183534 | 0,019594191 | -0,10201611 | -0,059 | 0,068 |
| hsa-miR-27b | -0,076755576 | -0,026307471 | -0,07333147 | -0,059 | 0,028 |
| hsa-miR-1264 | -0,050885042 | -0,066402622 | 0,56132755 | 0,148 | 0,358 |
| hsa-miR-3665 | 0,129013092 | -0,121276281 | -0,18268283 | -0,058 | 0,165 |
| hsa-miR-1245 | -0,06208198 | -0,014822611 | -0,09789431 | -0,058 | 0,042 |
| hsa-miR-4713-3p | 0,004888582 | -0,106635524 | -0,0730423 | -0,058 | 0,057 |
| hsa-miR-548t | -0,072471819 | -0,066049998 | -0,03546047 | -0,058 | 0,020 |
| hsa-miR-210 | -0,04661616 | -0,106441624 | -0,02016957 | -0,058 | 0,044 |
| hsa-miR-449c* | 0,069949385 | -0,197204554 | -0,04521736 | -0,057 | 0,134 |
| hsa-miR-4716-3p | 0,035300374 | -0,014778465 | -0,19293206 | -0,057 | 0,120 |
| hsa-miR-3919 | -0,076935013 | -0,011031146 | -0,08414145 | -0,057 | 0,040 |
| hsa-miR-27a | 0,014035375 | -0,028613603 | -0,15724994 | -0,057 | 0,089 |
| hsa-miR-548b-5p | 0,050027363 | -0,09268786 | -0,12892099 | -0,057 | 0,095 |
| hsa-miR-133a | -0,004266843 | -0,141067459 | -0,02618482 | -0,057 | 0,073 |
| hsa-miR-4788 | -0,02504306 | -0,145927867 | -0,00047557 | -0,057 | 0,078 |
| hsa-miR-4319 | -0,199741763 | 0,0463673 | -0,01765707 | -0,057 | 0,128 |
| hsa-miR-4763-5p | 0,004159917 | -0,013254207 | -0,16167933 | -0,057 | 0,091 |
| hsa-miR-617 | -0,018303738 | -0,070532633 | -0,08137267 | -0,057 | 0,034 |
| hsa-miR-1193 | 0,022033622 | -0,322911567 | 0,13188747 | -0,056 | 0,237 |
| hsa-miR-138-1* | -0,056274663 | -2,8099461906152+4,53236014182719i |  | -0,056 | #DIV/0! |
| hsa-miR-572 | -0,099232338 | 0,025400673 | -0,09425679 | -0,056 | 0,071 |
| hsa-miR-874 | -0,162242038 | -0,033540882 | 0,02926037 | -0,056 | 0,098 |
| hsa-miR-19a | -0,077327525 | -0,105004327 | 0,01603545 | -0,055 | 0,063 |
| hsa-miR-3132 | 0,013434159 | 0,013061345 | -0,19180064 | -0,055 | 0,118 |
| hsa-miR-4738-5p | 0,022841065 | -0,080993891 | -0,10668818 | -0,055 | 0,069 |
| hsa-miR-148b | -0,065221541 | 0,050419934 | -0,14852771 | -0,054 | 0,100 |
| hsa-miR-524-3p | -0,194368713 | -0,005560727 | 0,03668022 | -0,054 | 0,123 |
| hsa-miR-3670 | 0,002874203 | 0,036522073 | -0,20224701 | -0,054 | 0,129 |
| hsa-miR-3920 | -0,012272728 | 0,93 | -0,09567236 | 0,274 | 0,570 |
| hsa-miR-4285 | -0,04732933 | -0,111303646 | -0,00196183 | -0,054 | 0,055 |
| hsa-miR-518c | 0,052645715 | -0,06215849 | -0,15012734 | -0,053 | 0,102 |
| hsa-miR-23b | 0,013124036 | -0,113732437 | -0,05851139 | -0,053 | 0,064 |
| hsa-miR-3938 | 0,0514664 | -0,109226095 | -0,09831701 | -0,052 | 0,090 |
| hsa-miR-1303 | -0,01587246 | -0,019475825 | -0,11994209 | -0,052 | 0,059 |
| hsa-miR-762 | 0,001473023 | -0,100125846 | -0,05642072 | -0,052 | 0,051 |
| hsa-miR-4448 | -0,048580372 | 0,056192178 | -0,16212984 | -0,052 | 0,109 |
| hsa-miR-2682 | -0,039585257 | -0,006715794 | -0,10796476 | -0,051 | 0,052 |
| hsa-miR-3202 | -0,106139317 | -0,130005912 | 0,08220248 | -0,051 | 0,116 |
| hsa-miR-4673 | -0,029979383 | -0,003249375 | -0,12022583 | -0,051 | 0,061 |
| hsa-miR-520a-5p | 0,08740501 | -0,132236615 | -0,10743169 | -0,051 | 0,120 |
| hsa-miR-199b-5p | 0,016111389 | -0,092957902 | -0,07483135 | -0,051 | 0,058 |
| hsa-miR-362-3p | -0,041663159 | 0,009640505 | -0,11930559 | -0,050 | 0,065 |
| hsa-miR-337-5p | -0,043214479 | 0,99 | -0,05740311 | 0,296 | 0,601 |
| hsa-miR-492 | 0,094671871 | -0,145603939 | -0,0997689 | -0,050 | 0,128 |
| hsa-miR-4422 | 0,006481251 | 0,035147655 | -0,1920724 | -0,050 | 0,124 |
| hsa-miR-126* | 0,099410644 | -0,133711314 | -0,11599748 | -0,050 | 0,130 |
| hsa-miR-575 | -0,222687216 | 0,12286926 | 0,65201488 | 0,184 | 0,441 |
| hsa-miR-4659a-3p | -0,047066877 | 0,060664003 | -0,16293315 | -0,050 | 0,112 |
| hsa-miR-186 | -0,049609693 | -0,008022818 | -0,08972348 | -0,049 | 0,041 |
| hsa-miR-4729 | -0,072997858 | 0,012379759 | -0,08626634 | -0,049 | 0,054 |
| hsa-miR-4789-3p | -0,277454861 | 0,050974052 | 0,07989514 | -0,049 | 0,198 |
| hsa-miR-3974 | 0,099743125 | -0,22653189 | -0,01948875 | -0,049 | 0,165 |
| hsa-miR-3197 | 0,069904335 | -0,09887637 | -0,11704034 | -0,049 | 0,103 |
| hsa-miR-520g | 0,048101236 | -0,083789388 | -0,11026115 | -0,049 | 0,085 |
| hsa-miR-3605-5p | -0,13335141 | -0,2967434 | 0,28463411 | -0,048 | 0,300 |
| hsa-miR-1469 | -0,087794643 | 0,000214696 | -0,05781607 | -0,048 | 0,045 |
| hsa-miR-4778-3p | 0,081456135 | -0,08265229 | -0,14390425 | -0,048 | 0,117 |
| hsa-miR-3649 | 0,09627638 | -0,238685166 | -0,00209701 | -0,048 | 0,172 |
| hsa-miR-4685-3p | 0,086002728 | -0,089939749 | -0,13922774 | -0,048 | 0,118 |
| hsa-miR-18a | -0,130633377 | -0,073274674 | 0,06134363 | -0,048 | 0,099 |
| hsa-miR-566 | -0,228641966 | -0,070165078 | 0,15718193 | -0,047 | 0,194 |
| hsa-miR-3156-3p | 0,02925848 | -0,091966773 | -0,07844065 | -0,047 | 0,066 |
| hsa-miR-4263 | 0,075493453 | -0,082563512 | -0,13405576 | -0,047 | 0,109 |
| hsa-miR-3613-3p | 0,114690351 | 0,023468072 | -0,27919501 | -0,047 | 0,206 |
| hsa-miR-4470 | 0,063228411 | 0,007330929 | -0,21106142 | -0,047 | 0,145 |
| hsa-miR-4520a-5p // hsa-miR-4520b-5p | 0,064131835 | -0,042334753 | -0,16217935 | -0,047 | 0,113 |
| hsa-miR-515-5p | -0,005199977 | 0,97 | -0,08836581 | 0,292 | 0,589 |
| hsa-miR-938 | 0,018376102 | -0,078802473 | -0,0798104 | -0,047 | 0,056 |
| hsa-miR-92b | -0,009121727 | -0,54919888 | -0,08315899 | -0,214 | 0,293 |
| hsa-miR-556-5p | -0,194585919 | 0,011382254 | 0,04493256 | -0,046 | 0,130 |
| hsa-miR-29a | -0,039453645 | -0,042179306 | -0,05550347 | -0,046 | 0,009 |
| hsa-miR-378 | -0,091698084 | -0,047043977 | 0,00309809 | -0,045 | 0,047 |
| hsa-miR-4638-3p | 0,019777269 | -0,064027772 | -0,08818832 | -0,044 | 0,057 |
| hsa-miR-4773 | 0,032111983 | 0,0174778 | -0,18198569 | -0,044 | 0,120 |
| hsa-miR-4452 | -0,023991723 | -0,058425472 | -0,04988868 | -0,044 | 0,018 |
| hsa-miR-3191 | -0,039289068 | 1,04 | -0,04876075 | 0,317 | 0,626 |
| hsa-miR-582-5p | 0,009407594 | -0,039918575 | -0,10123711 | -0,044 | 0,055 |
| hsa-miR-3154 | 0,02228685 | 0,020031841 | -0,17256023 | -0,043 | 0,112 |
| hsa-miR-2053 | -0,029669929 | 0,021067025 | -0,12071798 | -0,043 | 0,072 |
| hsa-miR-219-5p | -0,005261679 | -0,043962483 | -0,07973783 | -0,043 | 0,037 |
| hsa-miR-5096 | 0,031364749 | -0,1206639 | -0,03952818 | -0,043 | 0,076 |
| hsa-miR-3591-5p | 0,030636295 | -0,097625547 | -0,06182649 | -0,043 | 0,066 |
| hsa-miR-206 | -0,090767164 | -0,02438163 | -0,01321324 | -0,043 | 0,042 |
| hsa-miR-3177-5p | 0,006755962 | 0,019119428 | -0,15385877 | -0,043 | 0,096 |
| hsa-miR-4700-5p | -0,028687114 | -0,060855508 | -0,03764999 | -0,042 | 0,017 |
| hsa-miR-4291 | 0,075179246 | -0,189501544 | -0,01286738 | -0,042 | 0,135 |
| hsa-miR-324-3p | 0,047831029 | -0,113952908 | -0,06104266 | -0,042 | 0,082 |
| hsa-miR-211 | 0,106577054 | -0,072160209 | -0,16141928 | -0,042 | 0,136 |
| hsa-miR-4445 | 0,044523336 | -0,148868347 | -0,02197806 | -0,042 | 0,098 |
| hsa-miR-136* | -0,015363308 | 0,672018793 | -0,06860564 | 0,196 | 0,413 |
| hsa-miR-548ai | 0,027022798 | -0,177273994 | 0,02591567 | -0,041 | 0,118 |
| hsa-miR-4710 | 0,02742156 | -0,036050829 | -0,11554273 | -0,041 | 0,072 |
| hsa-miR-4456 | -0,016218093 | -0,01956578 | -0,08820478 | -0,041 | 0,041 |
| hsa-miR-567 | 0,019623307 | 0,008980875 | -0,15253721 | -0,041 | 0,096 |
| hsa-miR-489 | -0,003789543 | 0,057721746 | -0,17698264 | -0,041 | 0,122 |
| hsa-miR-484 | -0,096020105 | -0,205336638 | 0,17875602 | -0,041 | 0,198 |
| hsa-miR-4646-5p | 0,025053835 | -0,001442841 | -0,14552016 | -0,041 | 0,092 |
| hsa-miR-520h | -0,019737856 | 0,027786515 | -0,12992668 | -0,041 | 0,081 |
| hsa-miR-3907 | -0,039650644 | -0,15133124 | 0,06937945 | -0,041 | 0,110 |
| hsa-miR-635 | 0,046355213 | -0,12704703 | -0,16315733 | -0,081 | 0,112 |
| hsa-miR-4737 | 0,050130605 | -0,171851678 | 0,00161 | -0,040 | 0,117 |
| hsa-miR-221 | -0,058676858 | -0,023764036 | -0,03727428 | -0,040 | 0,018 |
| hsa-miR-142-3p | -0,106746728 | -0,107494486 | 0,09522297 | -0,040 | 0,117 |
| hsa-miR-650 | -0,273348281 | -0,040037028 | 0,19445649 | -0,040 | 0,234 |
| hsa-miR-4691-3p | 0,02097467 | -0,01646957 | -0,12165435 | -0,039 | 0,074 |
| hsa-miR-3162-5p | 0,00782217 | -0,080776679 | -0,04264668 | -0,039 | 0,044 |
| hsa-miR-4514 | 0,007678831 | 0,016123003 | -0,13936426 | -0,039 | 0,087 |
| hsa-miR-122* | 0,031178578 | -0,613035475 | -0,10811492 | -0,230 | 0,339 |
| hsa-miR-639 | -0,083602271 | 0,031541086 | -0,06315792 | -0,038 | 0,061 |
| hsa-miR-4722-5p | 0,082447034 | -0,04982728 | -0,14727352 | -0,038 | 0,115 |
| hsa-miR-4745-3p | -0,010944688 | -0,125480714 | 0,02309504 | -0,038 | 0,078 |
| hsa-miR-1913 | 0,089314522 | -0,080734404 | -0,12130675 | -0,038 | 0,112 |
| hsa-miR-129-5p | -0,105002969 | -0,061687912 | 0,05465427 | -0,037 | 0,083 |
| hsa-miR-193b* | 0,012521326 | -0,12272311 | -0,00182274 | -0,037 | 0,074 |
| hsa-miR-556-3p | 0,07912198 | -0,126379534 | -0,06375899 | -0,037 | 0,105 |
| hsa-miR-1471 | 0,091431471 | -0,203096622 | 0,00192581 | -0,037 | 0,151 |
| hsa-miR-302a* | 0,026768994 | -1,454708004 | -0,09971054 | -0,509 | 0,821 |
| hsa-miR-548k | 0,064411606 | -0,031741275 | -0,14202775 | -0,036 | 0,103 |
| hsa-miR-600 | 0,019504596 | -0,00702158 | -0,12178447 | -0,036 | 0,075 |
| hsa-miR-3671 | -0,099601422 | 0,01535774 | -0,02365195 | -0,036 | 0,058 |
| hsa-miR-3196 | -0,281663935 | 0,050823352 | 0,12373209 | -0,036 | 0,216 |
| hsa-miR-3934 | 0,05961016 | -0,161774032 | -0,00412957 | -0,035 | 0,114 |
| hsa-miR-450b-3p | -0,169100584 | -0,026681596 | 0,08957486 | -0,035 | 0,130 |
| hsa-miR-4667-5p | 0,062304333 | -0,076500965 | -0,09150913 | -0,035 | 0,085 |
| hsa-miR-875-5p | -0,084125268 | -0,020840964 | -0,00041785 | -0,035 | 0,044 |
| hsa-miR-3913-5p | 0,081893671 | 0,026260564 | -0,21353468 | -0,035 | 0,157 |
| hsa-miR-200a* | 0,079460002 | -0,122818571 | -0,06184999 | -0,035 | 0,104 |
| hsa-miR-4686 | 0,002042689 | -0,008204395 | -0,09823993 | -0,035 | 0,055 |
| hsa-miR-512-5p | 0,045225789 | -0,119927143 | -0,02951532 | -0,035 | 0,083 |
| hsa-miR-216b | 0,003078544 | -0,00904012 | -0,09810978 | -0,035 | 0,055 |
| hsa-miR-4776-5p | -0,006664469 | -0,044976463 | -0,05217417 | -0,035 | 0,024 |
| hsa-miR-624 | 0,175113417 | -0,10023587 | -0,1785809 | -0,035 | 0,186 |
| hsa-miR-4725-5p | -0,010349585 | -0,045345352 | -0,04695434 | -0,034 | 0,021 |
| hsa-miR-384 | -0,015627641 | 1,03 | -0,05258789 | 0,321 | 0,615 |
| hsa-miR-591 | 0,018811814 | -0,134312361 | 0,01403531 | -0,034 | 0,087 |
| hsa-miR-1273c | -0,119509565 | 0,091968681 | -0,07379431 | -0,034 | 0,111 |
| hsa-miR-4694-5p | 0,051993154 | -0,081013425 | -0,07214684 | -0,034 | 0,074 |
| hsa-miR-579 | -0,064828284 | -0,033435593 | -0,00282162 | -0,034 | 0,031 |
| hsa-miR-3619-3p | 0,012090579 | -0,120718956 | 0,00775823 | -0,034 | 0,075 |
| hsa-let-7g | -0,05420758 | 0,019851861 | -0,06629565 | -0,034 | 0,047 |
| hsa-miR-4446-5p | 0,040157967 | -0,074464994 | -0,0657266 | -0,033 | 0,064 |
| hsa-miR-3116 | 0,040108557 | -0,0440684 | -0,09536286 | -0,033 | 0,068 |
| hsa-miR-130a | -0,1290303 | 0,13546698 | -0,10391108 | -0,032 | 0,146 |
| hsa-miR-3149 | -0,00848161 | -0,079324016 | -0,00934714 | -0,032 | 0,041 |
| hsa-miR-4642 | 0,030253802 | -0,095802919 | -0,03138201 | -0,032 | 0,063 |
| hsa-miR-483-3p | 0,050666289 | -0,004677059 | -0,14266329 | -0,032 | 0,100 |
| hsa-miR-3138 | 0,026199074 | 0,99 | -0,09023325 | 0,309 | 0,593 |
| hsa-miR-4293 | 0,033891436 | -0,075905735 | -0,05352858 | -0,032 | 0,058 |
| hsa-miR-141* | -0,063332197 | 0,001506066 |  | -0,031 | 0,046 |
| hsa-miR-30a | -0,050695091 | 0,063508369 | -0,10515623 | -0,031 | 0,086 |
| hsa-miR-1244 | 0,010504105 | -0,110270799 | 0,0075017 | -0,031 | 0,069 |
| hsa-miR-3199 | 0,024907116 | 0,115448547 | -0,23153122 | -0,030 | 0,180 |
| hsa-miR-661 | 0,021392199 | -0,065049746 | -0,04749565 | -0,030 | 0,046 |
| hsa-miR-4294 | 0,028815801 | -0,154620835 | 0,03497273 | -0,030 | 0,108 |
| hsa-miR-374b | -0,07536114 | 0,071649546 | -0,08645977 | -0,030 | 0,088 |
| hsa-miR-4529-5p | -0,003847939 | -0,056038211 | -0,71819553 | -0,259 | 0,398 |
| hsa-miR-769-3p | 0,016072457 | 0,096829753 | -0,20269991 | -0,030 | 0,155 |
| hsa-miR-4521 | 0,033659966 | -0,058360337 | -0,06498213 | -0,030 | 0,055 |
| hsa-miR-4750 | 0,015334472 | 0,015573381 | -0,12001982 | -0,030 | 0,078 |
| hsa-miR-19b | -0,055536178 | -0,038831507 | 0,00685661 | -0,029 | 0,032 |
| hsa-miR-4713-5p | -0,053919424 | -0,004259957 | 0,48101087 | 0,141 | 0,296 |
| hsa-miR-1914 | 0,14410596 | -0,238477264 | 0,00767091 | -0,029 | 0,194 |
| hsa-miR-616* | -0,047988448 | 0,696127482 | -0,0097042 | 0,213 | 0,419 |
| hsa-miR-4298 | 0,08449637 | -0,086039711 | -0,0848301 | -0,029 | 0,098 |
| hsa-miR-576-3p | 0,07179773 | -0,118558639 | -0,03938971 | -0,029 | 0,096 |
| hsa-miR-4321 | 0,119949512 | -0,086490603 | -0,11904673 | -0,029 | 0,130 |
| hsa-miR-1911 | 0,04718977 | 0,96 | -0,10357638 | 0,301 | 0,575 |
| hsa-miR-4704-3p | 0,017569094 | -0,046447575 | -0,05537461 | -0,028 | 0,040 |
| hsa-miR-331-3p | -0,159070848 | -0,004215002 | 0,07927977 | -0,028 | 0,121 |
| hsa-miR-3192 | 0,046894149 | -0,068747628 | -0,05996885 | -0,027 | 0,064 |
| hsa-miR-3200-5p | -0,115019211 | -0,016531786 | 0,05126996 | -0,027 | 0,084 |
| hsa-miR-4689 | 0,067084942 | 0,000334734 | -0,14743715 | -0,027 | 0,110 |
| hsa-miR-4657 | -0,033358034 | 0,026767453 | -0,07326074 | -0,027 | 0,050 |
| hsa-miR-1909 | 0,009758625 | 0,040286119 | -0,12958395 | -0,027 | 0,091 |
| hsa-miR-647 | -0,012843624 | -0,001272391 | -0,06521216 | -0,026 | 0,034 |
| hsa-miR-605 | 0,040522964 | -0,022332478 | -0,09712964 | -0,026 | 0,069 |
| hsa-miR-4305 | 0,056016702 | 0,010633198 | -0,14470522 | -0,026 | 0,105 |
| hsa-miR-3652 | 0,027210059 | -0,020925865 | -0,08401414 | -0,026 | 0,056 |
| hsa-miR-4462 | 0,065133002 | -0,090080452 | -0,05245782 | -0,026 | 0,081 |
| hsa-miR-583 | 0,030476703 | 0,007633403 | -0,11491802 | -0,026 | 0,078 |
| hsa-miR-184 | -0,147568688 | -0,077037613 | 0,14784428 | -0,026 | 0,154 |
| hsa-miR-3681* | 0,024805588 | -0,075919515 |  | -0,026 | 0,071 |
| hsa-miR-181b | -0,028961009 | 0,045668467 | -0,09274382 | -0,025 | 0,069 |
| hsa-miR-4459 | 0,181024632 | 0,02671971 | -0,28362651 | -0,025 | 0,237 |
| hsa-miR-548b-3p | 0,021033764 | -0,053273262 | -0,04362381 | -0,025 | 0,040 |
| hsa-miR-548x | 0,060981391 | -0,039213303 | -0,09657925 | -0,025 | 0,080 |
| hsa-miR-4467 | -0,035914767 | 0,093755364 | -0,13193059 | -0,025 | 0,113 |
| hsa-miR-1180 | 0,051733929 | 0,020402659 | -0,14563295 | -0,024 | 0,106 |
| hsa-miR-4260 | 0,134829376 | -0,00981244 | -0,19827304 | -0,024 | 0,167 |
| hsa-miR-627 | 0,017433463 | -0,025044173 | -0,0648053 | -0,024 | 0,041 |
| hsa-miR-1976 | 0,045455868 | 0,019274505 | -0,13638147 | -0,024 | 0,098 |
| hsa-miR-3131 | -0,021737735 | 0,05732034 | -0,10681897 | -0,024 | 0,082 |
| hsa-miR-513c | 0,007604187 | 0,036782916 | -0,11561707 | -0,024 | 0,081 |
| hsa-miR-4753-3p | 0,008860167 | 0,000701221 | -0,08033274 | -0,024 | 0,049 |
| hsa-miR-4795-3p | -0,000159302 | -0,024765134 | -0,04558575 | -0,024 | 0,023 |
| hsa-miR-3129-3p | 0,135264166 | -0,006492672 | -0,19913142 | -0,023 | 0,168 |
| hsa-miR-133b | -0,088627088 | -0,029632222 | 0,05231625 | -0,022 | 0,071 |
| hsa-miR-4303 | 0,098195731 | -0,068734576 | -0,09512027 | -0,022 | 0,105 |
| hsa-miR-1200 | 0,047772056 | -0,057696539 | -0,05468515 | -0,022 | 0,060 |
| hsa-miR-1468 | -0,020782418 | 0,004536271 | -0,04798946 | -0,021 | 0,026 |
| hsa-miR-518e | -0,02279562 | -0,15183732 | 0,11081196 | -0,021 | 0,131 |
| hsa-miR-29b | -0,077229535 | 0,066685554 | -0,05290107 | -0,021 | 0,077 |
| hsa-miR-598 | 0,064278279 | 0,036401277 | -0,16408069 | -0,021 | 0,125 |
| hsa-miR-548aj | 0,031479138 | -0,051214192 | -0,04259369 | -0,021 | 0,045 |
| hsa-miR-889 | -0,041489738 | 0,03756633 | -0,05725772 | -0,020 | 0,051 |
| hsa-miR-644 | -0,010700951 | -0,001983954 | -0,04770883 | -0,020 | 0,024 |
| hsa-miR-3146 | 0,111167681 | -0,003265211 | -0,16825976 | -0,020 | 0,140 |
| hsa-miR-539 | -0,09817433 | 0,000175927 | 0,03843497 | -0,020 | 0,070 |
| hsa-miR-3171 | 0,105513789 | -0,014947344 | -0,14995682 | -0,020 | 0,128 |
| hsa-miR-3944-5p | 0,052070383 | -0,040695136 | -0,07015498 | -0,020 | 0,064 |
| hsa-miR-4316 | 0,108892958 | -0,112783408 | -0,0544419 | -0,019 | 0,115 |
| hsa-miR-1281 | 0,010885174 | -0,132169168 | 0,06307969 | -0,019 | 0,101 |
| hsa-miR-587 | 0,040491022 | -0,113802375 | 0,01549697 | -0,019 | 0,083 |
| hsa-miR-1539 | 0,031367799 | -0,122510129 | 0,03381889 | -0,019 | 0,090 |
| hsa-miR-4421 | -0,054674677 | 0,053521413 | -0,05470996 | -0,019 | 0,062 |
| hsa-miR-16-2* | 0,00972141 | -0,046437309 |  | -0,018 | 0,040 |
| hsa-miR-423-5p | -0,070852547 | 0,055306999 | -0,0379012 | -0,018 | 0,065 |
| hsa-miR-3713 | 0,121736548 | -0,184037625 | 0,00907844 | -0,018 | 0,155 |
| hsa-miR-1273 | -0,05404732 | 0,93 | 0,01857162 | 0,298 | 0,548 |
| hsa-miR-4464 | 0,036969665 | -0,05687053 | -0,03232341 | -0,017 | 0,049 |
| hsa-miR-335 | -0,016934673 | 0,039930238 | -0,07505389 | -0,017 | 0,057 |
| hsa-miR-3545-3p | 0,089865986 | -0,126193693 | -0,0148833 | -0,017 | 0,108 |
| hsa-miR-3927 | 0,16006358 | -0,085071134 | -0,12611063 | -0,017 | 0,155 |
| hsa-miR-4513 | -0,217196499 | -0,031248215 | 0,19800007 | -0,017 | 0,208 |
| hsa-miR-548w | -0,284275282 | 0,003648362 | 0,23027279 | -0,017 | 0,258 |
| hsa-miR-144 | -0,021304442 | 0,030412599 | -0,05871737 | -0,017 | 0,045 |
| hsa-miR-526b* | -0,032789586 | 1,125892739 | -0,0001638 | 0,364 | 0,660 |
| hsa-miR-4486 | -0,19970645 | 0,033071166 | 0,11740746 | -0,016 | 0,164 |
| hsa-miR-1470 | 0,077460341 | -0,01247679 | -0,11404744 | -0,016 | 0,096 |
| hsa-miR-4302 | 0,009869414 | 0,008564629 | -0,06701828 | -0,016 | 0,044 |
| hsa-miR-29c | -0,093492673 | -0,036314822 | 0,08154954 | -0,016 | 0,089 |
| hsa-miR-132 | -0,016523184 | 0,025187275 | -0,05678041 | -0,016 | 0,041 |
| hsa-miR-2355-3p | -0,273357981 | 0,056632291 | 0,16933782 | -0,016 | 0,230 |
| hsa-miR-219-2-3p | -0,093293478 | 0,072224677 | -0,02615157 | -0,016 | 0,083 |
| hsa-miR-219-1-3p | -0,04843412 | 0,100954322 | -0,09955669 | -0,016 | 0,104 |
| hsa-miR-626 | 0,053962914 | 1,03 | -0,08531046 | 0,333 | 0,608 |
| hsa-miR-4280 | 0,097630663 | -0,09444562 | -0,05005862 | -0,016 | 0,101 |
| hsa-miR-4282 | 0,116260431 | -0,040149259 | -0,12204167 | -0,015 | 0,121 |
| hsa-miR-592 | -0,268482094 | 0,022667614 | 0,20002422 | -0,015 | 0,237 |
| hsa-miR-106a* | -0,070658142 | 0,041152256 |  | -0,015 | 0,079 |
| hsa-miR-3064-5p | -0,060412495 | 0,079572632 | -0,06339647 | -0,015 | 0,082 |
| hsa-miR-339-3p | -0,00129054 | -0,085982507 | 0,04404866 | -0,014 | 0,066 |
| hsa-miR-766 | 0,089163115 | -0,078487882 | -0,05313993 | -0,014 | 0,090 |
| hsa-miR-940 | -0,049962338 | 0,007415972 | 0,00024557 | -0,014 | 0,031 |
| hsa-miR-4721 | 0,117854418 | 0,017998157 | -0,17789367 | -0,014 | 0,150 |
| hsa-miR-24 | -0,149405044 | -0,006177821 | 0,11369974 | -0,014 | 0,132 |
| hsa-miR-3147 | 0,018286436 | -0,162208862 | 0,10217576 | -0,014 | 0,135 |
| hsa-miR-604 | -0,044777446 | 0,035772669 | -0,03181961 | -0,014 | 0,043 |
| hsa-miR-488 | -0,004960196 | -0,025925914 | -0,00991098 | -0,014 | 0,011 |
| hsa-miR-921 | 0,016920332 | -0,111087714 | 0,05409779 | -0,013 | 0,087 |
| hsa-miR-4264 | 0,129314684 | -0,077294101 | -0,091481 | -0,013 | 0,124 |
| hsa-miR-4444 | 0,040886496 | -0,038162968 | -0,04161895 | -0,013 | 0,047 |
| hsa-miR-4722-3p | 0,047491149 | 0,008741406 | -0,09485998 | -0,013 | 0,074 |
| hsa-miR-4741 | 0,119525857 | -0,05919337 | -0,09860977 | -0,013 | 0,116 |
| hsa-miR-509-5p | 0,05565007 | 0,000561628 | -0,0944632 | -0,013 | 0,076 |
| hsa-miR-487b | -0,116683218 | 0,080717833 | -0,00220769 | -0,013 | 0,099 |
| hsa-miR-145 | 0,037906789 | -0,053613016 | -0,02241191 | -0,013 | 0,047 |
| hsa-miR-183* | 0,059952259 | -0,071601261 | -0,02611923 | -0,013 | 0,067 |
| hsa-miR-4804-3p | 0,061614384 | 1,04 | -0,08670399 | 0,338 | 0,612 |
| hsa-miR-4769-3p | 0,08580591 | -0,123663126 | 0,00027653 | -0,013 | 0,105 |
| hsa-miR-487a | 0,10159988 | -0,075352574 | -0,0618257 | -0,012 | 0,098 |
| hsa-miR-1294 | -0,013640451 | 0,027918957 | -0,04822601 | -0,011 | 0,038 |
| hsa-miR-3687 | 0,070867904 | 0,033493763 | -0,13703544 | -0,011 | 0,111 |
| hsa-miR-3529 | 0,075932234 | 0,071616534 | -0,17902788 | -0,010 | 0,146 |
| hsa-miR-29b-2* | -0,043555474 | -1,268477642 | 0,02265855 | -0,430 | 0,727 |
| hsa-miR-576-5p | -0,00020106 | 0,034324902 | -0,06538447 | -0,010 | 0,051 |
| hsa-miR-370 | -0,096877102 | 0,041121943 | 0,02510858 | -0,010 | 0,075 |
| hsa-miR-4650-5p | -0,013122315 | 0,005639245 | -0,02306176 | -0,010 | 0,015 |
| hsa-miR-3168 | 0,028405907 | 0,019552974 | -0,07826667 | -0,010 | 0,059 |
| hsa-miR-1268 | 0,059612498 | -0,132882714 | 0,04304831 | -0,010 | 0,107 |
| hsa-miR-3137 | 0,049713213 | 0,001083293 | -0,08085451 | -0,010 | 0,066 |
| hsa-miR-4506 | 0,114503554 | -0,067411994 | -0,07668778 | -0,010 | 0,108 |
| hsa-miR-4746-3p | 0,12004056 | -0,067389017 | -0,08125411 | -0,010 | 0,112 |
| hsa-miR-1825 | -0,014296502 | 0,94 | -0,00461096 | 0,307 | 0,548 |
| hsa-miR-181a | -0,050250058 | 0,013818289 | 0,00809694 | -0,009 | 0,035 |
| hsa-miR-4489 | -0,043579957 | -0,027300392 | 0,04290287 | -0,009 | 0,046 |
| hsa-miR-1912 | 0,012912056 | 0,060556753 | -0,10141483 | -0,009 | 0,083 |
| hsa-miR-1265 | 0,028206425 | 0,079925853 | -0,13273728 | -0,008 | 0,111 |
| hsa-miR-4691-5p | 0,050659597 | -0,041469652 | -0,03354225 | -0,008 | 0,051 |
| hsa-miR-2964a-3p | 0,106574924 | -0,011425886 | -0,11943578 | -0,008 | 0,113 |
| hsa-miR-4757-3p | 0,157632719 | 0,048642009 | -0,23048595 | -0,008 | 0,200 |
| hsa-miR-200b | -0,048369632 | 0,042193507 | -0,01802182 | -0,008 | 0,046 |
| hsa-miR-1273g | 0,049804284 | -0,092842138 | 0,01936762 | -0,008 | 0,075 |
| hsa-miR-651 | 0,017841288 | 0,040013758 | -0,0810474 | -0,008 | 0,064 |
| hsa-miR-215 | 0,069615876 | -0,025804536 | -0,0662273 | -0,007 | 0,070 |
| hsa-miR-4528 | -0,002957116 | 0,062060535 | -0,08100127 | -0,007 | 0,072 |
| hsa-miR-4443 | 0,16622036 | -0,051314571 | -0,13653241 | -0,007 | 0,156 |
| hsa-miR-548g | 0,163013152 | 0,040841099 | -0,22526699 | -0,007 | 0,199 |
| hsa-miR-4714-5p | -0,00818166 | -0,083989596 | 0,07168858 | -0,007 | 0,078 |
| hsa-miR-4525 | -0,016107585 | 0,004783923 | -0,00893353 | -0,007 | 0,011 |
| hsa-miR-571 | 0,020597615 | -0,064885231 | 0,02494923 | -0,006 | 0,051 |
| hsa-miR-4531 | -0,240875933 | -0,004476405 | 0,22665557 | -0,006 | 0,234 |
| hsa-miR-586 | 0,063087091 | -0,065036951 | -0,01607576 | -0,006 | 0,065 |
| hsa-miR-3169 | -0,065478449 | 0,118654233 | -0,07079771 | -0,006 | 0,108 |
| hsa-miR-4787-3p | -0,033699745 | 0,039852802 | -0,02355433 | -0,006 | 0,040 |
| hsa-miR-3662 | 0,055696721 | -0,0028082 | -0,06906167 | -0,005 | 0,062 |
| hsa-miR-3975 | 0,124130707 | -0,148344231 | 0,00807272 | -0,005 | 0,137 |
| hsa-miR-4680-3p | 0,024010811 | 1,04 | -0,03464321 | 0,343 | 0,604 |
| hsa-miR-378f | 0,098825061 | 0,017110906 | -0,13181372 | -0,005 | 0,117 |
| hsa-miR-518a-3p | 0,008812134 | 0,022367433 | -0,04703382 | -0,005 | 0,037 |
| hsa-miR-181a* | 0,138947868 | -0,073805296 | -0,07982074 | -0,005 | 0,125 |
| hsa-miR-3656 | 0,069162516 | 0,006817368 | -0,09033915 | -0,005 | 0,080 |
| hsa-miR-4769-5p | 0,128598097 | -0,186509043 | 0,04380268 | -0,005 | 0,163 |
| hsa-miR-3691-5p | -0,163137028 | 0,007421005 | 0,1423011 | -0,004 | 0,153 |
| hsa-miR-4484 | 0,061275478 | 0,057008219 | -0,1302244 | -0,004 | 0,109 |
| hsa-miR-652 | -0,04875384 | -0,022530395 | 0,05948021 | -0,004 | 0,056 |
| hsa-miR-573 | 0,171982886 | -0,072537103 | -0,11014328 | -0,004 | 0,153 |
| hsa-miR-603 | 0,072939685 | -0,091990714 | 0,0084509 | -0,004 | 0,083 |
| hsa-miR-3678-3p | -0,057407574 | 0,017053985 | 0,02983586 | -0,004 | 0,047 |
| hsa-miR-653 | -0,074202333 | 0,093854632 | -0,03004998 | -0,003 | 0,087 |
| hsa-miR-4697-3p | -0,067085364 | 0,066491565 | -0,00889375 | -0,003 | 0,067 |
| hsa-miR-3180-5p | 0,015039037 | 0,063720211 | -0,08711099 | -0,003 | 0,077 |
| hsa-miR-892a | 0,022011621 | 0,006726179 | -0,03683652 | -0,003 | 0,031 |
| hsa-miR-548ae | 0,015509073 | 0,00419529 | -0,02747978 | -0,003 | 0,022 |
| hsa-miR-4724-3p | 0,295154629 | -0,088369497 | -0,21446332 | -0,003 | 0,265 |
| hsa-miR-599 | 0,02444643 | 0,050577728 | -0,0804326 | -0,002 | 0,069 |
| hsa-miR-151b | -0,046195519 | 0,065931767 | -0,02465357 | -0,002 | 0,060 |
| hsa-miR-4799-5p | 0,07418002 | -0,061371613 | -0,01616534 | -0,001 | 0,069 |
| hsa-miR-648 | 0,031989483 | 0,06344443 | -0,09815493 | -0,001 | 0,086 |
| hsa-miR-548a-5p | 0,037881246 | -0,063524621 | 0,02314056 | -0,001 | 0,055 |
| hsa-miR-193b | 0,118421353 | -0,193671996 | 0,07284133 | -0,001 | 0,169 |
| hsa-miR-452 | 0,081153026 | 0,040211907 | -0,12325748 | -0,001 | 0,108 |
| hsa-miR-2392 | 0,053309365 | 0,088854203 | -0,14355691 | 0,000 | 0,125 |
| hsa-miR-612 | 0 | 0 | 0 | 0,000 | 0,000 |
| hsa-miR-548l | 0,011031603 | -0,04825625 | 0,03723553 | 0,000 | 0,044 |
| hsa-miR-1273f | -0,063707092 | 0,138404509 | -0,07366765 | 0,000 | 0,120 |
| hsa-miR-495 | 0,03181508 | 0,009075883 | -0,0391255 | 0,001 | 0,036 |
| hsa-miR-623 | -0,116157839 | -0,062289275 | 0,18071098 | 0,001 | 0,158 |
| hsa-miR-581 | 0,128219132 | 0,038918235 | -0,16383188 | 0,001 | 0,150 |
| hsa-miR-4504 | -0,25469822 | 0,091467302 | 0,1666495 | 0,001 | 0,225 |
| hsa-miR-154 | -0,094134945 | 0,059034459 | 0,03974635 | 0,002 | 0,083 |
| hsa-miR-30e | -0,023623199 | 0,010189246 | 0,01871829 | 0,002 | 0,022 |
| hsa-miR-4253 | 0,071556501 | -0,00792843 | -0,05719094 | 0,002 | 0,065 |
| hsa-miR-584 | 0,109124638 | -0,179307989 | 0,07822006 | 0,003 | 0,158 |
| hsa-miR-4720-3p | 0,618468898 | 0,005263268 | 0,00086461 | 0,208 | 0,355 |
| hsa-miR-202 | 0,09325586 | 0,023227631 | -0,10682623 | 0,003 | 0,102 |
| hsa-miR-30e* | 0,10787653 | -0,09692449 | -0,00022605 | 0,004 | 0,102 |
| hsa-miR-3177-3p | -0,068926831 | 0,041788368 | 0,0388254 | 0,004 | 0,063 |
| hsa-miR-4799-3p | 0,002701794 | 0,126002311 | -0,1170165 | 0,004 | 0,122 |
| hsa-miR-3620 | -0,008336923 | 0,063209515 | -0,04290118 | 0,004 | 0,054 |
| hsa-miR-708 | -0,02199851 | 0,100162179 | -0,06547189 | 0,004 | 0,086 |
| hsa-miR-3973 | 0,143186039 | -0,066583356 | -0,06384181 | 0,004 | 0,120 |
| hsa-miR-361-3p | 0,14571966 | -0,150071951 | 0,01798057 | 0,005 | 0,148 |
| hsa-miR-885-3p | 0,070622476 | 0,034210222 | -0,09096929 | 0,005 | 0,085 |
| hsa-miR-1284 | 0,075886407 | 0,029541476 | -0,09021016 | 0,005 | 0,086 |
| hsa-miR-3174 | 0,218799302 | -0,084450694 | -0,11809747 | 0,005 | 0,186 |
| hsa-miR-4771 | 0,071054064 | 0,065490952 | -0,11971408 | 0,006 | 0,109 |
| hsa-miR-1254 | 0,038121624 | 0,112740094 | -0,1339705 | 0,006 | 0,127 |
| hsa-miR-711 | -0,071122001 | 0,030600939 | 0,06023099 | 0,007 | 0,069 |
| hsa-miR-3928 | 0,097532704 | 0,086707895 | -0,16436359 | 0,007 | 0,148 |
| hsa-miR-3922-5p | -0,043280699 | 0,048706058 | 0,01463889 | 0,007 | 0,047 |
| hsa-miR-18b* | 0,070048253 | -1,002144168 | -0,05654753 | -0,330 | 0,586 |
| hsa-miR-548n | 0,093089291 | 0,99 | -0,07957156 | 0,335 | 0,574 |
| hsa-miR-372 | 0,121644833 | -0,071555747 | -0,02956287 | 0,007 | 0,102 |
| hsa-miR-3144-5p | 0,0649374 | 0,018582719 | -0,06208466 | 0,007 | 0,064 |
| hsa-miR-554 | 0,046683764 | 0,074203779 | -0,09918754 | 0,007 | 0,093 |
| hsa-miR-373 | 0,094313145 | 0,98 | -0,07982523 | 0,331 | 0,568 |
| hsa-miR-1225-5p | 0,07501198 | -0,012123078 | -0,04083753 | 0,007 | 0,060 |
| hsa-miR-3917 | 0,205335992 | -0,033527157 | -0,149714 | 0,007 | 0,181 |
| hsa-miR-4653-3p | 0,165833063 | -0,147567994 | 0,00500763 | 0,008 | 0,157 |
| hsa-miR-4509 | 0,168003003 | -0,082323285 | -0,06232873 | 0,008 | 0,139 |
| hsa-miR-337-3p | -0,020850102 | 0,039578034 | 0,00489979 | 0,008 | 0,030 |
| hsa-miR-4652-3p | 0,049847212 | 0,071263601 | -0,09629276 | 0,008 | 0,091 |
| hsa-miR-3647-3p | 0,005881914 | 0,062337059 | -0,04041458 | 0,009 | 0,051 |
| hsa-miR-4740-3p | -0,246808034 | 0,044197562 | 0,23052522 | 0,009 | 0,241 |
| hsa-miR-3650 | -0,196316231 | 0,016652523 | 0,20854595 | 0,010 | 0,203 |
| hsa-miR-4758-5p | -0,727989022 | 0,063388487 | -0,04408581 | -0,236 | 0,429 |
| hsa-miR-891b | -0,059415724 | -0,011767943 | 0,10107191 | 0,010 | 0,082 |
| hsa-miR-3654 | 0,016662825 | -0,080034065 | 0,09340405 | 0,010 | 0,087 |
| hsa-miR-4315 | 0,116371995 | -0,022384124 | -0,0635853 | 0,010 | 0,094 |
| hsa-miR-4466 | 0,017775103 | 0,020488028 | -0,00768643 | 0,010 | 0,016 |
| hsa-miR-3151 | 0,085364299 | -0,011188181 | -0,04359012 | 0,010 | 0,067 |
| hsa-miR-520d-5p | 0,105045444 | 0,011343115 | -0,08562475 | 0,010 | 0,095 |
| hsa-miR-34b | 0,029937091 | 0,05459646 | -0,0537552 | 0,010 | 0,057 |
| hsa-miR-103b | 0,164783357 | 0,004775783 | -0,13873158 | 0,010 | 0,152 |
| hsa-miR-548d-5p | 0,137818493 | 0,024366285 | -0,13110195 | 0,010 | 0,135 |
| hsa-miR-4307 | -0,062195876 | 0,014275139 | 0,08015597 | 0,011 | 0,071 |
| hsa-miR-1237 | -0,094056785 | 0,08829516 | 0,03823967 | 0,011 | 0,094 |
| hsa-miR-4442 | -0,24296702 | 0,102385095 | 0,17318186 | 0,011 | 0,223 |
| hsa-miR-1183 | 0,089313436 | 0,097380467 | -0,15387996 | 0,011 | 0,143 |
| hsa-miR-92a | 0,183262331 | 0,010750669 | -0,15970294 | 0,011 | 0,171 |
| hsa-miR-670 | -0,050745494 | 0,072759258 | 0,01284262 | 0,012 | 0,062 |
| hsa-miR-466 | 0,124620434 | -0,056051891 | -0,03359838 | 0,012 | 0,098 |
| hsa-miR-3647-5p | 0,169868187 | -0,040137209 | -0,0937946 | 0,012 | 0,139 |
| hsa-miR-4792 | 0,112848201 | -0,092224537 | 0,01535247 | 0,012 | 0,103 |
| hsa-miR-632 | 0,15512975 | -0,040689507 | -0,07818113 | 0,012 | 0,125 |
| hsa-miR-16-1* | -0,018237224 | 0,042503527 |  | 0,012 | 0,043 |
| hsa-miR-630 | 0,058889149 | -0,320607495 | 0,29814821 | 0,012 | 0,312 |
| hsa-miR-622 | 0,213643588 | 0,002331919 | -0,17935305 | 0,012 | 0,197 |
| hsa-miR-3184 | 0,004866821 | 0,089537529 | -0,05714575 | 0,012 | 0,074 |
| hsa-miR-935 | -0,086562704 | 0,047567645 | 0,07653352 | 0,013 | 0,087 |
| hsa-miR-615-5p | -0,053419547 | -0,016466434 | 0,10805165 | 0,013 | 0,085 |
| hsa-miR-208b | -0,052780403 | 0,084334161 | 0,0075883 | 0,013 | 0,069 |
| hsa-miR-1290 | 0,088806205 | -0,028116353 | -0,0213742 | 0,013 | 0,066 |
| hsa-miR-4774-5p | 0,134569339 | -0,140486677 | 0,04533837 | 0,013 | 0,140 |
| hsa-miR-552 | 0,028564668 | 0,125541848 | -0,11418998 | 0,013 | 0,121 |
| hsa-miR-10a | -0,115910467 | 0,081263513 | 0,07473314 | 0,013 | 0,112 |
| hsa-miR-4490 | 0,106266034 | 0,067339069 | -0,13313 | 0,013 | 0,128 |
| hsa-miR-4262 | 0,096708842 | 0,091595325 | -0,14762748 | 0,014 | 0,140 |
| hsa-miR-148b* | -0,073619459 | 0,100795184 |  | 0,014 | 0,123 |
| hsa-miR-4645-5p | 0,128984569 | 0,020635821 | -0,10883428 | 0,014 | 0,119 |
| hsa-miR-4472 | 0,060350758 | -0,006222686 | -0,01274773 | 0,014 | 0,040 |
| hsa-miR-548f | 0,091998844 | 1,02 | -0,06435088 | 0,349 | 0,586 |
| hsa-miR-4477a | 0,008794923 | 0,066534186 | -0,03373768 | 0,014 | 0,050 |
| hsa-miR-4502 | -0,018382938 | 0,017793488 | 0,04318219 | 0,014 | 0,031 |
| hsa-miR-4258 | 0,047031616 | -0,048655509 | 0,04443178 | 0,014 | 0,055 |
| hsa-miR-4289 | -0,316986259 | 0,109258482 | 0,25119556 | 0,014 | 0,296 |
| hsa-miR-4487 | -0,007214374 | 0,05017373 | 0,00057892 | 0,015 | 0,031 |
| hsa-miR-4758-3p | 0,0503236 | 0,002495632 | -0,00830089 | 0,015 | 0,031 |
| hsa-miR-3673 | 0,065109969 | -0,036892208 | 0,01655051 | 0,015 | 0,051 |
| hsa-miR-4762-3p | -0,042940903 | 0,086266266 | 0,00156311 | 0,015 | 0,066 |
| hsa-miR-30a* | 0,137571667 | -0,083490124 | -0,00896057 | 0,015 | 0,112 |
| hsa-miR-429 | 0,06827498 | 0,052015753 | -0,07287373 | 0,016 | 0,077 |
| hsa-miR-4476 | 0,003848415 | -0,014319716 | 0,05798689 | 0,016 | 0,038 |
| hsa-miR-4637 | -0,01046364 | 0,022175963 | 0,03639786 | 0,016 | 0,024 |
| hsa-miR-4287 | 0,080323216 | 0,072442085 | -0,10442554 | 0,016 | 0,104 |
| hsa-miR-298 | -0,014987741 | 0,125196247 | -0,06176612 | 0,016 | 0,097 |
| hsa-miR-4703-5p | 0,041824967 | 0,070374704 | -0,06313496 | 0,016 | 0,070 |
| hsa-miR-1228 | 0,125169283 | -0,084597118 | 0,00915249 | 0,017 | 0,105 |
| hsa-miR-302f | 0,177755131 | -0,098397025 | -0,02842398 | 0,017 | 0,144 |
| hsa-miR-221* | 0,014923565 | 0,019179563 |  | 0,017 | 0,003 |
| hsa-miR-3198 | 0,061404064 | 0,019849039 | -0,03000848 | 0,017 | 0,046 |
| hsa-miR-4714-3p | 0,083845799 | -0,03499249 | 0,00289318 | 0,017 | 0,061 |
| hsa-miR-1224-3p | 0,084705692 | -0,10765199 | 0,07539993 | 0,017 | 0,108 |
| hsa-miR-4711-5p | -0,017109232 | 1,04 | 0,05213433 | 0,358 | 0,591 |
| hsa-miR-4318 | 0,101952767 | -0,19774014 | 0,14865709 | 0,018 | 0,188 |
| hsa-miR-4717-5p | 0,075468677 | -0,171166896 | 0,14873198 | 0,018 | 0,168 |
| hsa-miR-1229 | 0,055843526 | -0,023131242 | 0,02046515 | 0,018 | 0,040 |
| hsa-miR-2114* | 0,066548827 | -0,031045331 |  | 0,018 | 0,069 |
| hsa-miR-658 | 0,138814087 | -0,027192941 | -0,05583842 | 0,019 | 0,105 |
| hsa-miR-1323 | 0,097717636 | 1,08 | -0,05999409 | 0,373 | 0,618 |
| hsa-miR-4682 | 0,017359782 | 0,141956014 | -0,10090148 | 0,019 | 0,121 |
| hsa-miR-759 | 0,058394889 | 0,092065915 | -0,09196693 | 0,019 | 0,098 |
| hsa-miR-4458 | -0,139354537 | 0,196575518 | 0,00162893 | 0,020 | 0,169 |
| hsa-miR-3666 | 0,101486081 | 0,040731083 | -0,08317115 | 0,020 | 0,094 |
| hsa-miR-199a-3p // hsa-miR-199b-3p | -0,000715729 | -0,045312892 | 0,10524248 | 0,020 | 0,077 |
| hsa-miR-485-3p | 0,123721478 | -0,059732182 | -0,0046733 | 0,020 | 0,094 |
| hsa-miR-2277-3p | -0,02121391 | 0,110430445 | -0,02987504 | 0,020 | 0,079 |
| hsa-miR-3065-3p | 0,102388227 | -0,015327399 | -0,02693121 | 0,020 | 0,072 |
| hsa-miR-30b* | -0,009468954 | 0,049808376 |  | 0,020 | 0,042 |
| hsa-miR-204 | -0,079731891 | 0,04193715 | 0,09845236 | 0,020 | 0,091 |
| hsa-miR-920 | -0,110412015 | 0,005483037 | 0,16581431 | 0,020 | 0,139 |
| hsa-miR-9 | -0,006390698 | -0,138147705 | 0,20573765 | 0,020 | 0,174 |
| hsa-miR-181d | -0,092891557 | 0,001684155 | 0,15246771 | 0,020 | 0,124 |
| hsa-miR-330-5p | -0,013753379 | -0,121248043 | 0,19668853 | 0,021 | 0,162 |
| hsa-miR-4772-5p | 0,121001494 | 0,049913076 | -0,1086131 | 0,021 | 0,118 |
| hsa-miR-1280 | 0,141964918 | -0,040946862 | -0,03842676 | 0,021 | 0,105 |
| hsa-miR-4753-5p | 0,094376378 | 0,069749272 | -0,09979853 | 0,021 | 0,106 |
| hsa-miR-645 | 0,179637866 | -0,056085079 | -0,05905513 | 0,021 | 0,137 |
| hsa-miR-19a* | 0,136958277 | 0,861586181 | -0,09381037 | 0,302 | 0,499 |
| hsa-miR-4299 | 0,063154973 | 0,057557004 | -0,05586328 | 0,022 | 0,067 |
| hsa-miR-106a | 0,082823504 | -0,059004135 | 0,04105754 | 0,022 | 0,073 |
| hsa-miR-4288 | -0,043835763 | 0,082819263 | 0,02601791 | 0,022 | 0,063 |
| hsa-miR-4743 | 0,078972229 | -0,007907227 | -0,00563237 | 0,022 | 0,050 |
| hsa-miR-4505 | 0,019964374 | 0,124435796 | -0,07861678 | 0,022 | 0,102 |
| hsa-miR-20b | -0,008982854 | -0,004153938 | 0,07969858 | 0,022 | 0,050 |
| hsa-miR-3190 | 0,101527191 | 0,041984738 | -0,0766322 | 0,022 | 0,091 |
| hsa-miR-431 | 0,018421263 | 0,175591836 | -0,12635332 | 0,023 | 0,151 |
| hsa-miR-374a | 0,081413806 | 0,055084898 | -0,06864659 | 0,023 | 0,080 |
| hsa-miR-4261 | 0,053301278 | 0,097452341 | -0,08266856 | 0,023 | 0,094 |
| hsa-miR-331-5p | -0,038598029 | 0,04618448 | 0,0610938 | 0,023 | 0,054 |
| hsa-miR-455-3p | 0,066837125 | 0,062873033 | -0,06049989 | 0,023 | 0,072 |
| hsa-miR-4755-3p | 0,079859639 | 0,045750242 | -0,05547117 | 0,023 | 0,070 |
| hsa-miR-4265 | 0,086146748 | 0,027789955 | -0,0429518 | 0,024 | 0,065 |
| hsa-miR-522 | 0,184663558 | -0,003738071 | -0,10885836 | 0,024 | 0,149 |
| hsa-miR-518f | 0,038982179 | -0,074035656 | 0,1075906 | 0,024 | 0,092 |
| hsa-miR-1908 | 0,142309871 | 0,032653518 | -0,10213956 | 0,024 | 0,122 |
| hsa-miR-3663-3p | 0,120767139 | 0,97 | -0,07216 | 0,340 | 0,554 |
| hsa-miR-200b* | 0,085013018 | -0,057609863 | 0,04569189 | 0,024 | 0,074 |
| hsa-miR-2116 | -0,136149758 | 0,126743924 | 0,08264878 | 0,024 | 0,141 |
| hsa-miR-4276 | 0,137340599 | -0,02703687 | -0,03656225 | 0,025 | 0,098 |
| hsa-miR-4735-3p | 0,189865288 | 0,038979774 | -0,15453129 | 0,025 | 0,173 |
| hsa-miR-3189-3p | 0,037594507 | -0,108339642 | 0,1457242 | 0,025 | 0,127 |
| hsa-miR-3926 | 0,058299362 | 0,127131459 | -0,11023122 | 0,025 | 0,122 |
| hsa-miR-194 | -0,122670472 | 0,075930829 | 0,12218592 | 0,025 | 0,130 |
| hsa-miR-548c-5p | 0,067301411 | 0,97 | -0,01673026 | 0,340 | 0,547 |
| hsa-miR-4495 | -0,217984343 | 0,0109672 | 0,2838068 | 0,026 | 0,251 |
| hsa-miR-770-5p | 0,066045005 | -0,006745097 | 0,01838653 | 0,026 | 0,037 |
| hsa-miR-1245b-5p | -0,034705288 | 0,126766016 | -0,01436988 | 0,026 | 0,088 |
| hsa-miR-513a-3p | 0,156839461 | -0,001772252 | -0,07734588 | 0,026 | 0,120 |
| hsa-miR-4474-5p | -0,065154497 | 0,054049525 | 0,08931699 | 0,026 | 0,081 |
| hsa-miR-99a* | -0,128279511 | 0,355999506 | -0,14948784 | 0,026 | 0,286 |
| hsa-miR-4309 | 0,095579874 | -0,116019885 | 0,0999225 | 0,026 | 0,123 |
| hsa-miR-3674 | 0,052552442 | 0,005196488 | 0,02181601 | 0,027 | 0,024 |
| hsa-miR-3668 | 0,028620529 | -0,012453836 | 0,06455672 | 0,027 | 0,039 |
| hsa-miR-4292 | 0,14693448 | -0,143975634 | 0,07795163 | 0,027 | 0,152 |
| hsa-miR-501-5p | 0,120938211 | -0,043921815 | 0,00398681 | 0,027 | 0,085 |
| hsa-miR-197 | 0,042888446 | 0,049008928 | -0,01044422 | 0,027 | 0,033 |
| hsa-miR-570 | -0,090320962 | 0,186222631 | -0,01391065 | 0,027 | 0,143 |
| hsa-miR-1255a | 0,098220398 | 1,02 | -0,04265036 | 0,359 | 0,577 |
| hsa-miR-324-5p | 0,0992253 | -0,1490278 | 0,13362399 | 0,028 | 0,154 |
| hsa-miR-146b-3p | 0,090821926 | -0,053246349 | 0,04676237 | 0,028 | 0,074 |
| hsa-miR-1184 | 0,038874845 | -0,119828336 | 0,16603794 | 0,028 | 0,143 |
| hsa-miR-595 | 0,008657072 | -0,030781624 | 0,10801287 | 0,029 | 0,072 |
| hsa-miR-4527 | 0,069875392 | 0,046857996 | -0,03040976 | 0,029 | 0,053 |
| hsa-miR-3680* | -0,031249297 | 0,089133723 |  | 0,029 | 0,085 |
| hsa-miR-181a-2* | 0,016509556 | 0,041451057 |  | 0,029 | 0,018 |
| hsa-miR-28-3p | -0,064382587 | 0,204363419 | -0,05272096 | 0,029 | 0,152 |
| hsa-miR-532-5p | 0,014310431 | 0,042592484 | 0,03074663 | 0,029 | 0,014 |
| hsa-miR-302b* | 0,092948985 | -0,985233937 | -0,03385347 | -0,309 | 0,589 |
| hsa-miR-4794 | 0,007313366 | 0,128757501 | -0,04689137 | 0,030 | 0,090 |
| hsa-miR-29c* | 0,123615438 | -0,028520924 | -0,00513225 | 0,030 | 0,082 |
| hsa-miR-668 | 0,176999721 | -0,190095016 | 0,10427662 | 0,030 | 0,194 |
| hsa-miR-137 | 0,046757838 | 0,100085418 | -0,05550723 | 0,030 | 0,079 |
| hsa-miR-548am | 0,141132032 | 0,061454747 | -0,11053519 | 0,031 | 0,129 |
| hsa-miR-4313 | 0,082474175 | 1,12 | -0,02108539 | 0,394 | 0,631 |
| hsa-miR-549 | 0,098061735 | -0,023068463 | 0,01793867 | 0,031 | 0,062 |
| hsa-miR-1203 | 0,123122961 | 0,007276443 | -0,036856 | 0,031 | 0,083 |
| hsa-miR-3186-5p | 0,09959099 | 0,018934432 | -0,02480963 | 0,031 | 0,063 |
| hsa-miR-519a | 0,09315889 | -0,072732918 | 0,07361645 | 0,031 | 0,091 |
| hsa-miR-4793-5p | 0,042392415 | 1,05 | 0,02127274 | 0,371 | 0,588 |
| hsa-miR-3152-5p | 0,093303587 | 0,085521984 | -0,08191947 | 0,032 | 0,099 |
| hsa-miR-497 | 0,256947708 | -0,111582017 | -0,04819751 | 0,032 | 0,197 |
| hsa-miR-1225-3p | 0,024087498 | 0,017334455 | 0,05579196 | 0,032 | 0,021 |
| hsa-miR-4748 | -0,021981568 | 0,053554271 | 0,06813261 | 0,033 | 0,048 |
| hsa-miR-20a | -0,066250736 | 0,117053953 | 0,04902775 | 0,033 | 0,093 |
| hsa-miR-421 | -0,028568622 | 0,116449261 | 0,01226157 | 0,033 | 0,075 |
| hsa-miR-425 | 0,000916492 | 0,042972181 | 0,05651767 | 0,033 | 0,029 |
| hsa-miR-490-5p | 0,017482758 | 0,060967917 | 0,02217484 | 0,034 | 0,024 |
| hsa-miR-578 | 0,104647532 | 0,073162356 | -0,07677009 | 0,034 | 0,097 |
| hsa-miR-3689d | 0,034429202 | 0,123956854 | -0,05679917 | 0,034 | 0,090 |
| hsa-miR-378e | 0,094878249 | 0,039432006 | -0,03137314 | 0,034 | 0,063 |
| hsa-miR-187 | -0,041298536 | 0,070182619 | 0,07431881 | 0,034 | 0,066 |
| hsa-miR-32 | -0,003411344 | 0,035472124 | 0,07146731 | 0,035 | 0,037 |
| hsa-miR-1234 | 0,004811273 | -0,015726498 | 0,11557437 | 0,035 | 0,071 |
| hsa-miR-3170 | 0,105469586 | 0,117292112 | -0,11767322 | 0,035 | 0,132 |
| hsa-miR-662 | 0,091719561 | 0,031119154 | -0,01749845 | 0,035 | 0,055 |
| hsa-miR-3145-3p | 0,050569425 | 1,02 | 0,01984781 | 0,363 | 0,569 |
| hsa-miR-378i | 0,247443474 | -0,116651982 | -0,02471805 | 0,035 | 0,189 |
| hsa-miR-4435 | 0,058374444 | 0,070822977 | -0,02289809 | 0,035 | 0,051 |
| hsa-miR-1252 | 0,066029993 | 0,032185373 | 0,00822136 | 0,035 | 0,029 |
| hsa-miR-3140-5p | 0,16027559 | 0,00557665 | -0,05939259 | 0,035 | 0,113 |
| hsa-miR-4786-3p | 0,094072655 | 0,058844543 | -0,0457495 | 0,036 | 0,073 |
| hsa-miR-4267 | 0,047976752 | 0,067300755 | -0,00679905 | 0,036 | 0,038 |
| hsa-miR-4461 | -0,008082142 | 0,232519639 | -0,11579118 | 0,036 | 0,178 |
| hsa-miR-671-3p | 0,043845547 | 0,211899601 | -0,14699224 | 0,036 | 0,180 |
| hsa-miR-4426 | 0,083649449 | -0,009157841 | 0,03459897 | 0,036 | 0,046 |
| hsa-let-7a-2* | 0,150044353 | 0,861443869 | -0,07691536 | 0,312 | 0,490 |
| hsa-miR-519d | 0,062384825 | 0,029357551 | 0,0185087 | 0,037 | 0,023 |
| hsa-miR-34c-5p | -0,020453001 | 0,07263128 | 0,05826711 | 0,037 | 0,050 |
| hsa-miR-659 | 0,071767533 | 0,050997013 | -0,01200759 | 0,037 | 0,044 |
| hsa-miR-4804-5p | 0,106980083 | -0,046189462 | 0,05007861 | 0,037 | 0,077 |
| hsa-miR-369-3p | -0,001519895 | 0,058871947 | 0,05378769 | 0,037 | 0,033 |
| hsa-miR-4520a-3p | 0,027274148 | 0,112309905 | -0,0283942 | 0,037 | 0,071 |
| hsa-miR-4699-3p | 0,104974704 | 1,16 | -0,03047005 | 0,412 | 0,652 |
| hsa-miR-545 | 0,151715047 | 0,052223616 | -0,09042418 | 0,038 | 0,122 |
| hsa-miR-4427 | 0,196618517 | -0,034552241 | -0,04824757 | 0,038 | 0,138 |
| hsa-miR-877* | 0,122739619 | -0,065943084 | 0,05784466 | 0,038 | 0,096 |
| hsa-miR-1282 | -0,119073283 | 0,02268774 | 0,21123409 | 0,038 | 0,166 |
| hsa-miR-1251 | -0,042209609 | 0,098412538 | 0,05869726 | 0,038 | 0,072 |
| hsa-miR-4259 | -0,172513907 | 0,017399217 | 0,27030268 | 0,038 | 0,222 |
| hsa-miR-3614-3p | 0,117927615 | -0,022540392 | 0,02118733 | 0,039 | 0,072 |
| hsa-miR-532-3p | -0,056793709 | 0,169949331 | 0,00365368 | 0,039 | 0,117 |
| hsa-miR-4480 | 0,079759721 | 0,065444901 | -0,02732261 | 0,039 | 0,058 |
| hsa-miR-2467-3p | 0,153506515 | -0,037623206 | 0,00290777 | 0,040 | 0,101 |
| hsa-miR-1910 | -0,006632794 | 0,120544593 | 0,00494444 | 0,040 | 0,070 |
| hsa-miR-4795-5p | 0,222604324 | -0,101444751 | -0,00208506 | 0,040 | 0,166 |
| hsa-miR-544b | 0,05115878 | 0,123770339 | -0,05559988 | 0,040 | 0,090 |
| hsa-miR-4676-5p | 0,076085684 | 0,038437677 | 0,00482428 | 0,040 | 0,036 |
| hsa-miR-3145-5p | 0,089094661 | 0,063090322 | -0,03249346 | 0,040 | 0,064 |
| hsa-miR-891a | 0,108873591 | -0,055982997 | 0,06797216 | 0,040 | 0,086 |
| hsa-miR-4447 | 0,113028876 | 0,063173433 | -0,05512923 | 0,040 | 0,086 |
| hsa-miR-151-3p | 0,070566228 | 0,064942765 | -0,01432368 | 0,040 | 0,047 |
| hsa-miR-383 | 0,141175603 | 0,094837549 | -0,11373544 | 0,041 | 0,136 |
| hsa-miR-548an | 0,037556889 | -0,06994772 | 0,15469391 | 0,041 | 0,112 |
| hsa-miR-548q | 0,136697465 | -0,007188449 | -0,00706896 | 0,041 | 0,083 |
| hsa-miR-4668-3p | 0,1226865 | 0,040918939 | -0,04115373 | 0,041 | 0,082 |
| hsa-miR-338-5p | -0,048576567 | 0,086705074 | 0,08448495 | 0,041 | 0,077 |
| hsa-miR-4534 | 0,126375441 | 0,087065386 | -0,09053219 | 0,041 | 0,116 |
| hsa-miR-30d | -0,000937318 | 0,069820974 | 0,05465438 | 0,041 | 0,037 |
| hsa-miR-494 | -0,028620575 | 0,150965097 | 0,00212193 | 0,041 | 0,096 |
| hsa-miR-942 | 0,02576863 | 1,05 | 0,057395 | 0,378 | 0,582 |
| hsa-miR-1263 | 0,115980207 | 0,080525313 | -0,07088746 | 0,042 | 0,099 |
| hsa-miR-4659b-5p | 0,063931069 | 0,018480194 | 0,0432914 | 0,042 | 0,023 |
| hsa-miR-3923 | 0,102077874 | 0,069547645 | -0,04419672 | 0,042 | 0,077 |
| hsa-miR-4503 | 0,100223682 | -0,178235783 | 0,20603668 | 0,043 | 0,198 |
| hsa-miR-4763-3p | -0,056168337 | 0,106253513 | 0,07851024 | 0,043 | 0,087 |
| hsa-miR-4699-5p | 0,034618279 | 0,117622922 | -0,02339067 | 0,043 | 0,071 |
| hsa-miR-4787-5p | 0,15807364 | 0,033511401 | -0,06257268 | 0,043 | 0,111 |
| hsa-miR-149 | -0,099891439 | 0,159236018 | 0,07052303 | 0,043 | 0,132 |
| hsa-miR-4272 | 0,152453758 | 0,012893016 | -0,03516868 | 0,043 | 0,097 |
| hsa-miR-375 | 0,031496532 | 0,080427477 | 0,01999746 | 0,044 | 0,032 |
| hsa-miR-144* | 0,000641314 | 0,193435714 | -0,06149308 | 0,044 | 0,133 |
| hsa-miR-3156-5p | -0,191041522 | 0,048921911 | 0,27508611 | 0,044 | 0,233 |
| hsa-miR-568 | 0,006730031 | 0,018748542 | 0,1076631 | 0,044 | 0,055 |
| hsa-miR-631 | 0,067065605 |  | 0,0218374 | 0,044 | 0,032 |
| hsa-miR-3661 | 0,114821549 | 0,069680708 | -0,05054934 | 0,045 | 0,085 |
| hsa-miR-24-2* | -0,020367044 | 0,110388801 |  | 0,045 | 0,092 |
| hsa-miR-4532 | 0,135095563 | 0,002071185 | -0,00154533 | 0,045 | 0,078 |
| hsa-miR-4482 | 0,10201806 | 0,99 | -0,00940471 | 0,361 | 0,548 |
| hsa-miR-380 | 0,067510089 | 0,102896376 | -0,03094836 | 0,046 | 0,069 |
| hsa-miR-4438 | 0,116524873 | -0,037642152 | 0,06060742 | 0,046 | 0,078 |
| hsa-miR-4423-3p | 0,139942532 | 0,065890325 | -0,06620598 | 0,047 | 0,104 |
| hsa-miR-378d | 0,071470983 | 0,015227666 | 0,05414933 | 0,047 | 0,029 |
| hsa-miR-2115* | 0,077848579 | 0,01610397 |  | 0,047 | 0,044 |
| hsa-miR-328 | -0,023200333 | 0,057116734 | 0,1073134 | 0,047 | 0,066 |
| hsa-miR-146a* | 0,037050905 | 0,0571208 |  | 0,047 | 0,014 |
| hsa-miR-518d-3p | 0,06044257 | -0,034109984 | 0,11502035 | 0,047 | 0,075 |
| hsa-miR-3140-3p | 0,128421955 | 0,145839796 | -0,13230921 | 0,047 | 0,156 |
| hsa-miR-4328 | 0,097512194 | 0,123921794 | -0,07878926 | 0,048 | 0,110 |
| hsa-miR-4790-5p | 0,153277989 | -0,004169278 | -0,006123 | 0,048 | 0,091 |
| hsa-miR-34a* | 0,121676225 | -0,025693566 |  | 0,048 | 0,104 |
| hsa-miR-3148 | 0,227975378 | 0,021572147 | -0,1055527 | 0,048 | 0,168 |
| hsa-miR-3182 | 0,202771679 | 0,016068528 | -0,07470838 | 0,048 | 0,141 |
| hsa-miR-31 | 0,006003271 | -0,013979036 | 0,15377049 | 0,049 | 0,092 |
| hsa-miR-205* | 0,118259195 | -0,021020153 |  | 0,049 | 0,098 |
| hsa-miR-553 | 0,149437713 | 1 | -0,05202519 | 0,366 | 0,558 |
| hsa-miR-512-3p | 0,094916375 | -0,057643738 | 0,10992563 | 0,049 | 0,093 |
| hsa-miR-3135b | -0,226736408 | 0,85 | -0,47573739 | 0,049 | 0,705 |
| hsa-miR-142-5p | -0,031740396 | -0,005242063 | 0,18459682 | 0,049 | 0,118 |
| hsa-miR-4286 | 0,184784519 | 1,01 | -0,08595556 | 0,370 | 0,571 |
| hsa-miR-4279 | 0,110748297 | -0,124441877 | 0,16412208 | 0,050 | 0,154 |
| hsa-miR-3936 | 0,063589222 | 0,092928749 | -0,00570444 | 0,050 | 0,051 |
| hsa-miR-5047 | 0,145479846 | 0,070396877 | -0,0650449 | 0,050 | 0,107 |
| hsa-miR-411 | 0,044696517 | 0,084852847 | 0,02208968 | 0,051 | 0,032 |
| hsa-miR-548y | 0,049545442 | 0,091025137 | 0,01160842 | 0,051 | 0,040 |
| hsa-miR-3144-3p | 0,148684772 | -0,007020499 | 0,0108449 | 0,051 | 0,085 |
| hsa-miR-302e | 0,096993945 | 0,137768906 | -0,08193852 | 0,051 | 0,117 |
| hsa-miR-3126-3p | 0,09353147 | 0,087146519 | -0,02777155 | 0,051 | 0,068 |
| hsa-miR-519e* | 0,179145273 | -0,076695227 |  | 0,051 | 0,181 |
| hsa-miR-299-3p | 0,15828402 | 0,049506593 | -0,0539014 | 0,051 | 0,106 |
| hsa-miR-4671-5p | 0,245578322 | -0,118661652 | 0,02740613 | 0,051 | 0,183 |
| hsa-miR-3667-3p | 0,021658479 | 0,119243251 | 0,0135569 | 0,051 | 0,059 |
| hsa-miR-1208 | 0,293491793 | 0,212977856 | -0,35186616 | 0,052 | 0,352 |
| hsa-miR-3911 | 0,03496154 | 1,09 | 0,06811628 | 0,398 | 0,600 |
| hsa-miR-4638-5p | 0,131848726 | 0,97 | -0,02865204 | 0,358 | 0,536 |
| hsa-miR-4756-3p | 0,085960109 | 0,0528528 | 0,01627645 | 0,052 | 0,035 |
| hsa-miR-642b | -0,047216861 | 0,036187898 | 0,1662667 | 0,052 | 0,108 |
| hsa-miR-548ab | 0,216658474 | 0,105564621 | -0,1668524 | 0,052 | 0,197 |
| hsa-miR-2861 | 0,237108108 | -0,034875825 | -0,04685502 | 0,052 | 0,161 |
| hsa-miR-548a-3p | 0,110076426 | 0,048914781 | -0,00341247 | 0,052 | 0,057 |
| hsa-miR-326 | 0,020156542 | 0,048415038 | 0,0873775 | 0,052 | 0,034 |
| hsa-miR-4757-5p | 0,107409454 | 0,113977567 | -0,06522308 | 0,052 | 0,102 |
| hsa-miR-498 | -0,014332517 | 0,151314065 | 0,01929252 | 0,052 | 0,088 |
| hsa-miR-4663 | 0,11600409 | 0,115374081 | -0,07483215 | 0,052 | 0,110 |
| hsa-miR-4732-3p | 0,164131504 | -0,056595775 | 0,04979174 | 0,052 | 0,110 |
| hsa-miR-26b | -0,010152 | 0,03203964 | 0,13578443 | 0,053 | 0,075 |
| hsa-miR-548i | 0,205335629 | 0,023044728 | -0,06984248 | 0,053 | 0,140 |
| hsa-miR-4776-3p | 0,098695372 | 0,034943821 | 0,02582037 | 0,053 | 0,040 |
| hsa-miR-3074-5p | 0,07122122 | 0,090312875 | -0,00158752 | 0,053 | 0,048 |
| hsa-miR-3667-5p | -0,066947505 | 0,077189658 | 0,15102255 | 0,054 | 0,111 |
| hsa-miR-559 | 0,00757023 | 0,138045249 | 0,01737192 | 0,054 | 0,073 |
| hsa-miR-3120-3p | 0,062467246 | 0,179098093 | -0,07712114 | 0,055 | 0,128 |
| hsa-miR-1243 | 0,06998211 | 0,163199856 | -0,06824985 | 0,055 | 0,116 |
| hsa-miR-128 | -0,062669465 | 0,002181145 | 0,22629057 | 0,055 | 0,152 |
| hsa-miR-3937 | 0,100890957 | -0,012267861 | 0,07787008 | 0,055 | 0,060 |
| hsa-miR-3614-5p | 0,053733503 | 0,05787585 | 0,05496985 | 0,056 | 0,002 |
| hsa-miR-4768-3p | 0,148366969 | 0,110407533 | 0,0007624 | 0,087 | 0,077 |
| hsa-miR-483-5p | 0,015210427 | -0,101169667 | 0,25353951 | 0,056 | 0,181 |
| hsa-miR-208a | -0,120910353 | 0,035541901 | 0,25393853 | 0,056 | 0,188 |
| hsa-miR-3136-3p | -0,005236544 | 0,017227663 | 0,15659208 | 0,056 | 0,088 |
| hsa-miR-3916 | 0,030868969 | 0,039681068 | 0,09932202 | 0,057 | 0,037 |
| hsa-miR-301b | 0,199030774 | 0,048783255 | -0,07792243 | 0,057 | 0,139 |
| hsa-miR-3921 | 0,073469881 | 0,040864958 | 0,05576628 | 0,057 | 0,016 |
| hsa-miR-4731-3p | 0,081433059 | 0,039514003 | 0,04954103 | 0,057 | 0,022 |
| hsa-miR-3175 | 0,085087811 | 0,032168524 | 0,05338298 | 0,057 | 0,027 |
| hsa-miR-4697-5p | 0,096956619 | 0,092338991 | -0,0182336 | 0,057 | 0,065 |
| hsa-miR-4766-3p | 0,17191067 | 0,112473721 | -0,11330499 | 0,057 | 0,150 |
| hsa-miR-4304 | 0,082325494 | 0,075926656 | 0,01322394 | 0,057 | 0,038 |
| hsa-miR-4760-5p | 0,128246765 | 1,04 | -0,01354562 | 0,385 | 0,572 |
| hsa-miR-21 | 0,032335192 | 0,110584043 | 0,02960076 | 0,058 | 0,046 |
| hsa-miR-1248 | -0,013233173 |  | 0,12828197 | 0,058 | 0,100 |
| hsa-miR-139-5p | -0,052677209 | 0,214356699 | 0,01161026 | 0,058 | 0,139 |
| hsa-miR-4761-3p | 0,109513424 | -0,06486996 | 0,12901515 | 0,058 | 0,107 |
| hsa-miR-548h | 0,218667559 | 0,076978938 | -0,12162058 | 0,058 | 0,171 |
| hsa-miR-1972 | 0,059320168 | 0,07154714 | 0,04428387 | 0,058 | 0,014 |
| hsa-miR-2681 | 0,121186086 | 1,01 | -0,00429385 | 0,376 | 0,553 |
| hsa-miR-4497 | 0,079229535 | 0,135668553 | -0,03949689 | 0,058 | 0,089 |
| hsa-miR-4429 | 0,144884777 | 0,016506655 | 0,01515736 | 0,059 | 0,075 |
| hsa-miR-514b-5p | 0,11763779 | 0,093816399 | -0,03470154 | 0,059 | 0,082 |
| hsa-miR-502-3p | 0,189773047 | 1,09 | -0,07167904 | 0,403 | 0,609 |
| hsa-miR-4537 | 0,921916759 | 0,121636604 | -0,00317686 | 0,347 | 0,502 |
| hsa-miR-3591-3p | 0,153264245 | 0,130145639 | -0,10533489 | 0,059 | 0,143 |
| hsa-miR-3648 | 0,160588673 | 0,031412199 | -0,01344148 | 0,060 | 0,090 |
| hsa-miR-3679-5p | 0,063118094 | 0,123851077 | -0,00777941 | 0,060 | 0,066 |
| hsa-miR-656 | 0,120868296 | 0,031215933 | 0,0275156 | 0,060 | 0,053 |
| hsa-miR-4439 | 0,07898788 | 0,117198117 | -0,01589117 | 0,060 | 0,069 |
| hsa-miR-1343 | 0,163920563 | 0,055743624 | -0,03904175 | 0,060 | 0,102 |
| hsa-miR-4658 | 0,196907884 | 0,041842401 | -0,05729591 | 0,060 | 0,128 |
| hsa-miR-4796-5p | -0,096027681 | -0,058455485 | 0,33613583 | 0,061 | 0,239 |
| hsa-miR-148a* | 0,072521514 | 0,017734338 | 0,09194353 | 0,061 | 0,038 |
| hsa-miR-614 | 0,235452273 | -0,012254152 | -0,03963788 | 0,061 | 0,152 |
| hsa-miR-4653-5p | 0,098956646 | 0,15663575 | -0,07099849 | 0,062 | 0,118 |
| hsa-miR-2114 | 0,150367919 | 0,117652822 | -0,08305605 | 0,062 | 0,126 |
| hsa-miR-1206 | -0,053969415 | 0,11287858 | 0,12616309 | 0,062 | 0,100 |
| hsa-miR-4681 | 0,146872115 | 0,075783494 | -0,03735971 | 0,062 | 0,093 |
| hsa-miR-1301 | 0,186984161 | 0,067682423 | -0,06900846 | 0,062 | 0,128 |
| hsa-miR-450b-5p | 0,062123769 | -0,081790335 | 0,20638636 | 0,062 | 0,144 |
| hsa-miR-4778-5p | 0,198837048 | -0,002759343 | -0,00903704 | 0,062 | 0,118 |
| hsa-miR-412 | -0,218141066 | 0,026412174 | 0,38024836 | 0,063 | 0,301 |
| hsa-miR-3189-5p | 0,070084443 | 0,155694616 | -0,03703534 | 0,063 | 0,097 |
| hsa-miR-3622b-5p | 0,119001775 | 0,080185818 | -0,00708274 | 0,064 | 0,065 |
| hsa-miR-4468 | 0,127713399 | 1,04 | 0,00087657 | 0,390 | 0,567 |
| hsa-miR-376c | -0,126823998 | 0,088988472 | 0,23115218 | 0,064 | 0,180 |
| hsa-miR-3127-3p | 0,097083042 | 0,134649288 | -0,03781257 | 0,065 | 0,091 |
| hsa-miR-1305 | 0,130558177 | 0,09739486 | -0,0333887 | 0,065 | 0,087 |
| hsa-miR-505 | 0,114301459 | 0,080537928 | -0,00024615 | 0,065 | 0,059 |
| hsa-miR-760 | -0,067180951 | 0,05680801 | 0,20605691 | 0,065 | 0,137 |
| hsa-miR-1238 | 0,143646412 | 0,04421817 | 0,00782519 | 0,065 | 0,070 |
| hsa-miR-939 | 0,1477086 | -0,017098754 | 0,06509155 | 0,065 | 0,082 |
| hsa-miR-875-3p | 0,042479991 | 0,08728304 | 0,06601596 | 0,065 | 0,022 |
| hsa-miR-3153 | 0,08227755 | 0,96 | 0,05042249 | 0,364 | 0,516 |
| hsa-miR-3972 | 0,120520242 | 0,110462833 | -0,03139869 | 0,067 | 0,085 |
| hsa-miR-23c | 0,054136383 | 0,129161458 | 0,01674553 | 0,067 | 0,057 |
| hsa-miR-3065-5p | 0,198856497 | -0,026326132 | 0,02796819 | 0,067 | 0,118 |
| hsa-miR-371-5p | 0,127320744 | 0,147970488 | -0,07477762 | 0,067 | 0,123 |
| hsa-miR-649 | 0,158694638 | -0,120906727 | 0,16286047 | 0,067 | 0,163 |
| hsa-miR-374a* | 0,00315409 | 0,13061933 |  | 0,067 | 0,090 |
| hsa-miR-3910 | 0,086940086 | 0,048494876 | 0,06527041 | 0,067 | 0,019 |
| hsa-miR-3179 | 0,157591465 | 0,013996059 | 0,02957755 | 0,067 | 0,079 |
| hsa-miR-149* | -0,096167902 | 0,230518675 |  | 0,067 | 0,231 |
| hsa-miR-4668-5p | 0,068076845 | 0,101953515 | 0,03256318 | 0,068 | 0,035 |
| hsa-miR-3655 | 0,165442443 | 1,13 | -0,02961767 | 0,422 | 0,621 |
| hsa-miR-1278 | 0,173277904 | 0,007903071 | 0,02259915 | 0,068 | 0,092 |
| hsa-miR-4436a | 0,195981324 | 0,040256296 | -0,03233254 | 0,068 | 0,117 |
| hsa-miR-517c | 0,126109621 | 0,081547422 | -0,0035967 | 0,068 | 0,066 |
| hsa-miR-888 | 0,100672313 | 1,01 | 0,03555971 | 0,382 | 0,545 |
| hsa-miR-548ah | 0,330168748 | -0,025808097 | -0,10000149 | 0,068 | 0,230 |
| hsa-miR-3200-3p | 0,130631905 | 1,06 | 0,00561889 | 0,399 | 0,576 |
| hsa-miR-519c-3p | 0,155007484 | -0,037401924 | 0,08784448 | 0,068 | 0,098 |
| hsa-miR-1 | -0,004802192 | 0,186203138 | 0,02527037 | 0,069 | 0,103 |
| hsa-miR-551a | 0,042501753 | 0,109435956 | 0,05518484 | 0,069 | 0,036 |
| hsa-miR-381 | -0,000588503 | 0,166543129 | 0,041689 | 0,069 | 0,087 |
| hsa-miR-4273 | 0,015154505 | 0,145165103 | 0,0475228 | 0,069 | 0,068 |
| hsa-miR-2052 | 0,180557579 | 0,106960294 | -0,07782838 | 0,070 | 0,133 |
| hsa-miR-4639-3p | 0,114333445 | 0,100709631 | -0,00524743 | 0,070 | 0,065 |
| hsa-miR-523 | -0,153460825 | 0,153492042 | 0,2101333 | 0,070 | 0,196 |
| hsa-miR-1181 | 0,150795999 | 0,94 | -0,00872021 | 0,361 | 0,508 |
| hsa-miR-4766-5p | 0,098057698 | 0,066144252 | 0,04905494 | 0,071 | 0,025 |
| hsa-miR-4782-3p | 0,060292272 | 0,18613041 | -0,0323918 | 0,071 | 0,110 |
| hsa-miR-4693-3p | 0,232444601 | 1,02 | -0,08927872 | 0,388 | 0,571 |
| hsa-miR-4746-5p | -0,096323826 | 0,007698903 | 0,30451646 | 0,072 | 0,208 |
| hsa-miR-222* | 0,032459083 | 0,111558555 |  | 0,072 | 0,056 |
| hsa-miR-596 | 0,179616652 | -0,085306051 | 0,12266337 | 0,072 | 0,139 |
| hsa-miR-660 | 0,149168286 | 0,103343057 | -0,03543707 | 0,072 | 0,096 |
| hsa-miR-4685-5p | 0,196395537 | 0,026973777 | -0,00616675 | 0,072 | 0,109 |
| hsa-miR-342-5p | 0,031729863 | 0,094923916 | 0,09141078 | 0,073 | 0,036 |
| hsa-miR-3123 | 0,133005639 | 1,06 | 0,01373635 | 0,402 | 0,573 |
| hsa-miR-33b* | 0,070585833 | 1,212718985 | 0,0765037 | 0,453 | 0,658 |
| hsa-miR-802 | 0,15180927 | 1,02 | -0,00459139 | 0,389 | 0,552 |
| hsa-miR-557 | 0,176167471 | 0,027051294 | 0,0192429 | 0,074 | 0,088 |
| hsa-miR-4783-3p | 0,099285226 | 0,107136233 | 0,01714315 | 0,075 | 0,050 |
| hsa-miR-514b-3p | 0,132256695 | 0,036990331 | 0,05521978 | 0,075 | 0,051 |
| hsa-miR-136 | 0,038118739 | 0,093295324 | 0,09319899 | 0,075 | 0,032 |
| hsa-miR-4436b-5p | -0,201084243 | 0,143685774 | 0,2858022 | 0,076 | 0,250 |
| hsa-miR-3689b* // hsa-miR-3689c | 0,247844651 | -0,016804252 | -0,00126689 | 0,077 | 0,149 |
| hsa-miR-4803 | 0,064714857 | 0,230340064 | -0,0647395 | 0,077 | 0,148 |
| hsa-miR-633 | 0,154391286 | 0,104459477 | -0,02853445 | 0,077 | 0,095 |
| hsa-miR-3622b-3p | 0,245000489 | 0,96 | -0,09134949 | 0,371 | 0,537 |
| hsa-miR-642a | 0,156863579 | 0,087343598 | -0,01353002 | 0,077 | 0,086 |
| hsa-miR-548p | 0,064450629 | 0,118749325 | 0,04867917 | 0,077 | 0,037 |
| hsa-miR-4295 | 0,118907044 | 0,085544213 | 0,02762233 | 0,077 | 0,046 |
| hsa-miR-1226 | 0,203454082 | 0,134989998 | -0,10580525 | 0,078 | 0,162 |
| hsa-miR-3152-3p | 0,158030005 | 0,132122126 | -0,05686748 | 0,078 | 0,117 |
| hsa-miR-4651 | 0,153397461 | 0,103886004 | -0,02342346 | 0,078 | 0,091 |
| hsa-miR-3651 | 0,114599685 | -0,108558235 | 0,23015434 | 0,079 | 0,172 |
| hsa-miR-3692 | 0,087344232 | -0,052612817 | 0,20155167 | 0,079 | 0,127 |
| hsa-miR-4320 | 0,172443678 | 0,053551889 | 0,01032743 | 0,079 | 0,084 |
| hsa-miR-508-3p | -0,039888377 | 0,060387731 | 0,21658029 | 0,079 | 0,129 |
| hsa-miR-4296 | 0,218059634 | 0,031937929 | -0,00822313 | 0,081 | 0,121 |
| hsa-miR-4671-3p | 0,182883849 | 0,073227741 | -0,01329532 | 0,081 | 0,098 |
| hsa-miR-519e | 0,197370728 | -0,082808495 | 0,12958393 | 0,081 | 0,146 |
| hsa-miR-3176 | 0,181450676 | 0,100101233 | -0,03727626 | 0,081 | 0,111 |
| hsa-miR-382 | 0,019197923 | 0,155765217 | 0,06992722 | 0,082 | 0,069 |
| hsa-miR-4690-5p | 0,141930695 | 0,005704272 | 0,09803469 | 0,082 | 0,070 |
| hsa-miR-501-3p | -0,005048425 | 0,198463905 | 0,05248684 | 0,082 | 0,105 |
| hsa-miR-924 | 0,082348091 | 0,063835852 | 0,10011498 | 0,082 | 0,018 |
| hsa-miR-4455 | 0,163367956 | 0,090444829 | -0,00738989 | 0,082 | 0,086 |
| hsa-miR-3118 | -0,010111346 | 0,222647161 | 0,03411379 | 0,082 | 0,124 |
| hsa-miR-2964a-5p | 0,074530186 | 0,116237222 | 0,05701797 | 0,083 | 0,030 |
| hsa-miR-24-1* | -0,0496592 | 0,215178155 |  | 0,083 | 0,187 |
| hsa-miR-4310 | 0,279517601 | -0,046027812 | 0,01484131 | 0,083 | 0,173 |
| hsa-miR-4755-5p | 0,152717929 | 1,04 | 0,01286404 | 0,402 | 0,557 |
| hsa-miR-3160-5p | 0,277907166 | 0,029313712 | -0,05723233 | 0,083 | 0,174 |
| hsa-miR-1827 | 0,062612798 | 0,161444913 | 0,0264933 | 0,084 | 0,070 |
| hsa-miR-4449 | 0,164151024 | 0,077364761 | 0,00914923 | 0,084 | 0,078 |
| hsa-miR-944 | 0,176675003 | 0,110803239 | -0,03677023 | 0,084 | 0,109 |
| hsa-miR-4767 | 0,150265054 | -0,053721842 | 0,1543678 | 0,084 | 0,119 |
| hsa-miR-3940-3p | 0,139405166 | 0,15898413 | -0,04728171 | 0,084 | 0,114 |
| hsa-miR-223* | 0,132836848 | 0,110628553 | 0,00802993 | 0,084 | 0,067 |
| hsa-miR-3143 | 0,197129475 | 0,003527118 | 0,05116717 | 0,084 | 0,101 |
| hsa-miR-520b | 0,267222201 | -0,033620653 | 0,02105355 | 0,085 | 0,160 |
| hsa-miR-4749-3p | 0,126673004 | 0,080846317 | 0,05020491 | 0,086 | 0,038 |
| hsa-miR-655 | 0,00552519 | 0,167078831 | 0,08556551 | 0,086 | 0,081 |
| hsa-miR-191 | 0,018966598 | 0,139736168 | 0,0997198 | 0,086 | 0,062 |
| hsa-miR-615-3p | -0,00780274 | 0,116667883 | 0,14969868 | 0,086 | 0,083 |
| hsa-miR-4431 | 0,094277183 | 0,146301161 | 0,01832801 | 0,086 | 0,064 |
| hsa-miR-890 | 0,23092993 | 0,040000946 | -0,01070901 | 0,087 | 0,127 |
| hsa-miR-1202 | 0,087818019 | 1,12 | 0,08574049 | 0,431 | 0,597 |
| hsa-miR-4720-5p | 0,150140611 | -0,00664881 | 0,11690465 | 0,087 | 0,083 |
| hsa-miR-4775 | 0,097738636 | 0,144252343 | 0,01866553 | 0,087 | 0,063 |
| hsa-miR-620 | 0,139881612 | -0,074313407 | 0,19572864 | 0,087 | 0,143 |
| hsa-miR-4696 | 0,090765792 | 0,211594215 | -0,04082823 | 0,087 | 0,126 |
| hsa-miR-3163 | 0,076720278 | 0,133299147 | 0,05382501 | 0,088 | 0,041 |
| hsa-miR-3664-5p | 0,134788595 | 0,092346884 | 0,03756858 | 0,088 | 0,049 |
| hsa-miR-4704-5p | 0,060970453 | 0,043363626 | 0,16099598 | 0,088 | 0,063 |
| hsa-miR-1537 | 0,133574966 | 0,007312008 | 0,12470873 | 0,089 | 0,070 |
| hsa-miR-10b | -0,032585686 | 0,226517629 | 0,07329612 | 0,089 | 0,130 |
| hsa-miR-1324 | 0,153129965 | 0,017067998 | 0,09825692 | 0,089 | 0,068 |
| hsa-miR-3194-3p | 0,180069173 | 0,134861051 | -0,04642193 | 0,090 | 0,120 |
| hsa-miR-3150a-5p | 0,09238562 | 1,02 | 0,08663645 | 0,400 | 0,537 |
| hsa-miR-25 | -0,012920675 | 0,125842151 | 0,1564798 | 0,090 | 0,090 |
| hsa-miR-548ak | 0,115712712 | 0,083789772 | 0,06997082 | 0,090 | 0,023 |
| hsa-miR-330-3p | 0,179701825 | 0,161458674 | -0,06914748 | 0,091 | 0,139 |
| hsa-miR-4649-3p | -0,003995429 | 0,004546254 | 0,27177292 | 0,091 | 0,157 |
| hsa-miR-22 | 0,089525444 | 0,168558099 | 0,01441191 | 0,091 | 0,077 |
| hsa-miR-1279 | 0,130295315 | 0,160012298 | -0,01755967 | 0,091 | 0,095 |
| hsa-miR-3960 | 0,076380597 | 0,223850208 | -0,02744087 | 0,091 | 0,126 |
| hsa-miR-127-5p | 0,064755294 | 0,101719559 | 0,10641068 | 0,091 | 0,023 |
| hsa-miR-4275 | 0,186001124 | 0,035207671 | 0,0519418 | 0,091 | 0,083 |
| hsa-miR-4676-3p | 0,116674577 | 0,049833607 | 0,1086512 | 0,092 | 0,036 |
| hsa-miR-1179 | 0,072404989 | 0,109047808 | 0,09390806 | 0,092 | 0,018 |
| hsa-miR-574-5p | 0,073374185 | 0,241057579 | -0,03854449 | 0,092 | 0,141 |
| hsa-miR-3688-3p | 0,068866027 | 1,08 | 0,11569783 | 0,422 | 0,571 |
| hsa-miR-1298 | 0,081575157 | 0,079775377 | 0,11799349 | 0,093 | 0,022 |
| hsa-miR-4798-5p | 0,085689618 | 0,07483416 | 0,12055916 | 0,094 | 0,024 |
| hsa-miR-199a-5p | -0,036377313 | 0,000586382 | 0,18705761 | 0,050 | 0,120 |
| hsa-miR-371b-5p | 0,092760153 | 0,095032228 | 0,15406078 | 0,114 | 0,035 |
| hsa-miR-500b | 0,175155526 | 0,060690732 | 0,04730416 | 0,094 | 0,070 |
| hsa-miR-548o | 0,098692324 | -0,043336446 | 0,22812113 | 0,094 | 0,136 |
| hsa-miR-3978 | 0,168956751 | 0,023387615 | 0,09452955 | 0,096 | 0,073 |
| hsa-miR-3130-5p | 0,109782098 | 1,1 | 0,08226433 | 0,431 | 0,580 |
| hsa-miR-624* | -0,065654227 | 0,345877699 | 0,00797549 | 0,096 | 0,219 |
| hsa-miR-200a | 0,038576368 | 0,091516741 | 0,15879475 | 0,096 | 0,060 |
| hsa-miR-100 | 0,140080243 | 0,10360685 | 0,0461294 | 0,097 | 0,047 |
| hsa-miR-1276 | -0,181507056 | 0,092282018 | 0,37917219 | 0,097 | 0,280 |
| hsa-miR-548d-3p | 0,147885396 | 0,094069117 | 0,04833533 | 0,097 | 0,050 |
| hsa-miR-582-3p | 0,177484433 | 0,141873054 | -0,02751298 | 0,097 | 0,110 |
| hsa-miR-1915 | 0,039487367 | 0,099533954 | 0,15282462 | 0,097 | 0,057 |
| hsa-miR-3606 | 0,159782769 | 0,138119907 | -0,00569739 | 0,097 | 0,090 |
| hsa-miR-33b | 0,0373301 | 0,154558809 | 0,10192696 | 0,098 | 0,059 |
| hsa-miR-3064-3p | 0,112905748 | 1,22 | 0,08319998 | 0,472 | 0,648 |
| hsa-miR-504 | 0,008391594 | 0,208620382 | 0,07982334 | 0,099 | 0,101 |
| hsa-miR-509-3-5p | 0,165203001 | 1,15 | 0,03296765 | 0,449 | 0,610 |
| hsa-miR-4661-5p | 0,19804951 | -0,187312461 | 0,28654194 | 0,099 | 0,252 |
| hsa-miR-3945 | 0,111223342 | -0,017758652 | 0,20442866 | 0,099 | 0,112 |
| hsa-miR-3621 | 0,114926681 | 0,070030548 | 0,11452013 | 0,100 | 0,026 |
| hsa-miR-92b* | 0,125934416 | 0,742719083 | 0,07424237 | 0,314 | 0,372 |
| hsa-miR-4479 | 0,160887643 | 0,016038326 | 0,12350949 | 0,100 | 0,075 |
| hsa-miR-302a | -0,036808586 | 0,328298057 | 0,00966805 | 0,100 | 0,199 |
| hsa-miR-3188 | -0,016658606 | 0,100597103 | 0,21785385 | 0,101 | 0,117 |
| hsa-miR-4760-3p | 0,088333822 | 0,214634549 | 0,00031796 | 0,101 | 0,108 |
| hsa-miR-517b | 0,085791349 | 0,116929282 | 0,1007284 | 0,101 | 0,016 |
| hsa-miR-3912 | 0,152550812 | 0,036284597 | 0,11615474 | 0,102 | 0,059 |
| hsa-miR-521 | 0,132253883 | 0,087754561 | 0,08759541 | 0,103 | 0,026 |
| hsa-miR-1273d | 0,152574346 | 0,134470548 | 0,02224703 | 0,103 | 0,071 |
| hsa-miR-1299 | 0,189692013 | 0,072683989 | 0,04729248 | 0,103 | 0,076 |
| hsa-miR-346 | 0,106475238 | 0,164971594 | 0,0385749 | 0,103 | 0,063 |
| hsa-miR-4666-3p | 0,135222911 | 0,000880302 | 0,17418569 | 0,103 | 0,091 |
| hsa-miR-657 | 0,246950417 | 0,138759619 | -0,07501901 | 0,104 | 0,164 |
| hsa-miR-4420 | 0,172817466 | 0,106226175 | 0,03180717 | 0,104 | 0,071 |
| hsa-miR-3119 | 0,155587315 | 0,149379913 | 0,00669539 | 0,104 | 0,084 |
| hsa-miR-141 | 0,016650633 | 0,140384512 | 0,15549096 | 0,104 | 0,076 |
| hsa-miR-126 | 0,049499213 | 0,230709379 | 0,03299256 | 0,104 | 0,110 |
| hsa-miR-3158-3p | 0,149596169 | 0,140259181 | 0,02335593 | 0,104 | 0,070 |
| hsa-miR-4251 | 0,11708549 | 0,226731443 | -0,03012985 | 0,105 | 0,129 |
| hsa-miR-198 | 0,180708424 | 0,144265727 | -0,0106706 | 0,105 | 0,102 |
| hsa-miR-4255 | 0,142017078 | 1,04 | 0,06804822 | 0,417 | 0,541 |
| hsa-miR-4481 | 0,182861773 | 0,103202332 | 0,02917667 | 0,105 | 0,077 |
| hsa-miR-4471 | 0,280846462 | 0,060737421 | -0,02522375 | 0,105 | 0,158 |
| hsa-miR-10b* | 0,097399482 | 0,182095004 | 0,03700166 | 0,105 | 0,073 |
| hsa-miR-4520b-3p | -0,029453129 |  | 0,24111713 | 0,106 | 0,191 |
| hsa-miR-3691-3p | 0,15696878 | 0,053063825 | 0,10779799 | 0,106 | 0,052 |
| hsa-miR-3622a-3p | 0,146058052 | 0,176501814 | -0,00109642 | 0,107 | 0,095 |
| hsa-miR-223 | 0,035281464 | 0,262110442 | 0,02415368 | 0,107 | 0,134 |
| hsa-miR-1261 | 0,130029026 | 0,076632742 | 0,11557068 | 0,107 | 0,028 |
| hsa-miR-618 | 0,121275488 | 0,158943024 | 0,04207367 | 0,107 | 0,060 |
| hsa-miR-378c | 0,1471964 | 0,054126692 | 0,12173131 | 0,108 | 0,048 |
| hsa-miR-4749-5p | -0,199521425 | 0,87 | -0,34545288 | 0,108 | 0,664 |
| hsa-miR-4641 | 0,088995185 | 0,184585849 | 0,05203325 | 0,109 | 0,068 |
| hsa-miR-3622a-5p | 0,106308213 | 0,103415137 | 0,11763072 | 0,109 | 0,008 |
| hsa-miR-4797-5p | 0,182700094 | 0,105605445 | 0,0404502 | 0,110 | 0,071 |
| hsa-miR-511 | 0,158765648 | 0,170652634 | 0,00089044 | 0,110 | 0,095 |
| hsa-miR-4450 | 0,093957436 | -0,005954447 | 0,24343102 | 0,110 | 0,126 |
| hsa-miR-892b | 0,145283729 | 0,143725144 | 0,04388234 | 0,111 | 0,058 |
| hsa-miR-4507 | 0,082815907 | 0,12839269 | 0,12362622 | 0,112 | 0,025 |
| hsa-miR-4297 | 0,211197901 | 0,011420198 | 0,11675095 | 0,113 | 0,100 |
| hsa-miR-340 | 0,040259052 | 0,268506706 | 0,03142708 | 0,113 | 0,134 |
| hsa-miR-1270 | 0,062936408 | 0,166619526 | 0,11117842 | 0,114 | 0,052 |
| hsa-miR-4777-3p | 0,210674977 | 0,127138444 | 0,00432929 | 0,114 | 0,104 |
| hsa-miR-676 | 0,342926899 | 0,083550545 | -0,08340212 | 0,114 | 0,215 |
| hsa-miR-452* | -0,057753782 | -1,07466378 | 1,47652583 | 0,115 | 1,284 |
| hsa-miR-4680-5p | 0,143856339 | 0,021143298 | 0,18015499 | 0,115 | 0,083 |
| hsa-miR-4647 | 0,145631512 | 0,013068128 | 0,18788487 | 0,116 | 0,091 |
| hsa-miR-3660 | 0,276866255 | -0,016314799 | 0,08622748 | 0,116 | 0,149 |
| hsa-miR-4465 | 0,08058828 | 0,102328327 | 0,16451365 | 0,116 | 0,044 |
| hsa-miR-4492 | 0,173404364 | 0,053977256 | 0,12217191 | 0,117 | 0,060 |
| hsa-miR-3617 | 0,177182618 | 0,195975248 | -0,02232296 | 0,117 | 0,121 |
| hsa-miR-601 | 0,171256869 | 0,017722355 | 0,16725924 | 0,119 | 0,088 |
| hsa-miR-155 | 0,189240209 | 0,064271242 | 0,10309082 | 0,119 | 0,064 |
| hsa-miR-4329 | 0,187546314 | 0,074989034 | 0,09489895 | 0,119 | 0,060 |
| hsa-miR-4667-3p | 0,14786768 | 0,096805283 | 0,11591208 | 0,120 | 0,026 |
| hsa-miR-320c | 0,225220133 | 0,076368082 | 0,0592005 | 0,120 | 0,091 |
| hsa-miR-2276 | 0,036531717 | 1,12 | 0,20505415 | 0,454 | 0,583 |
| hsa-miR-590-3p | 0,094132488 | 0,386183569 | -0,11759495 | 0,121 | 0,253 |
| hsa-miR-720 | 0,106417098 | 0,103654111 | 0,15432905 | 0,121 | 0,028 |
| hsa-miR-362-5p | 0,194472573 | 0,109020775 | 0,06131958 | 0,122 | 0,067 |
| hsa-miR-4762-5p | -0,046297564 | -0,039196528 | 0,45081527 | 0,122 | 0,285 |
| hsa-miR-1258 | 0,024204825 | 0,071154708 | 0,27107168 | 0,122 | 0,131 |
| hsa-miR-555 | 0,247451258 | -0,055799908 | 0,17489296 | 0,122 | 0,158 |
| hsa-miR-224* | 0,281849911 | -0,036498973 |  | 0,123 | 0,225 |
| hsa-miR-3618 | 0,062938308 | 0,189936488 | 0,1172536 | 0,123 | 0,064 |
| hsa-miR-409-3p | 0,027244684 | 0,16368317 | 0,17931678 | 0,123 | 0,084 |
| hsa-miR-139-3p | 0,045740342 | 0,101609267 | 0,22316889 | 0,124 | 0,091 |
| hsa-miR-4693-5p | 0,144854953 | 0,206757407 | 0,01916446 | 0,124 | 0,096 |
| hsa-miR-205 | 0,060415746 | 0,08470384 | 0,22580056 | 0,124 | 0,089 |
| hsa-miR-625 | 0,159127055 | 0,104630706 | 0,10824155 | 0,124 | 0,030 |
| hsa-miR-3609 | 0,121309314 | 0,113346614 | 0,13756281 | 0,124 | 0,012 |
| hsa-miR-4701-5p | 0,09012238 | 0,126218754 | 0,15891084 | 0,125 | 0,034 |
| hsa-miR-4716-5p | -0,162546329 | 0,82 | -0,28140446 | 0,125 | 0,605 |
| hsa-miR-4518 | 0,170191378 | 0,196086347 | 0,01036957 | 0,126 | 0,101 |
| hsa-miR-191* | 0,068563249 | 0,182872176 |  | 0,126 | 0,081 |
| hsa-miR-671-5p | 0,029492469 | 0,252621769 | 0,09751158 | 0,127 | 0,114 |
| hsa-miR-17 | 0,014689961 | 0,096423459 | 0,2705338 | 0,127 | 0,131 |
| hsa-miR-3193 | 0,093375931 | 0,214662337 | 0,07579373 | 0,128 | 0,076 |
| hsa-miR-4707-3p | 0,187549761 | 0,184889384 | 0,01178978 | 0,128 | 0,101 |
| hsa-miR-561 | 0,241325168 | 0,108635782 | 0,03682366 | 0,129 | 0,104 |
| hsa-miR-432 | 0,196398101 | 0,142244575 | 0,04869016 | 0,129 | 0,075 |
| hsa-miR-934 | 0,193441829 | 0,192694553 | 0,00152653 | 0,129 | 0,111 |
| hsa-miR-3607-3p | 0,205959442 | 1,04 | 0,05314985 | 0,433 | 0,531 |
| hsa-miR-4493 | 0,223947523 | 0,18082767 | -0,01605006 | 0,130 | 0,128 |
| hsa-miR-3121-3p | 0,172896681 | 0,196768937 | 0,01941234 | 0,130 | 0,096 |
| hsa-miR-3653 | 0,222226542 | 0,146471191 | 0,02045025 | 0,130 | 0,102 |
| hsa-miR-628-3p | 0,134994265 | 0,241584695 | 0,01435392 | 0,130 | 0,114 |
| hsa-miR-1182 | 0,110439322 | 0,15522238 | 0,1253295 | 0,130 | 0,023 |
| hsa-miR-373* | 0,030193163 | 0,369983648 | -0,00816429 | 0,131 | 0,208 |
| hsa-miR-4669 | 0,146844132 | 0,095332818 | 0,15652257 | 0,133 | 0,033 |
| hsa-miR-520f | -0,057433582 | 0,156519337 | 0,29978299 | 0,133 | 0,180 |
| hsa-miR-4727-5p | 0,177427084 | 0,149298583 | 0,07282669 | 0,133 | 0,054 |
| hsa-miR-550b | 0,165064181 | 0,218728594 | 0,01723878 | 0,134 | 0,104 |
| hsa-miR-3976 | 0,112805206 | -0,054747999 | 0,34508817 | 0,134 | 0,201 |
| hsa-miR-3942-3p | -0,022387603 | 0,14237806 | 0,28348479 | 0,134 | 0,153 |
| hsa-miR-4709-5p | 0,224685365 | 0,158342984 | 0,02308816 | 0,135 | 0,103 |
| hsa-miR-3181 | 0,014320653 | -0,006211929 | 0,39801459 | 0,135 | 0,228 |
| hsa-miR-4705 | 0,15037209 | 0,202088751 | 0,05537759 | 0,136 | 0,074 |
| hsa-miR-3167 | 0,256157713 | 0,109598041 | 0,0429978 | 0,136 | 0,109 |
| hsa-miR-214 | 0,044193366 | 0,287793564 | 0,07874945 | 0,137 | 0,132 |
| hsa-miR-1204 | 0,242935525 | 0,114353701 | 0,05520736 | 0,137 | 0,096 |
| hsa-miR-4715-3p | 0,146745754 | 0,208358281 | 0,05751204 | 0,138 | 0,076 |
| hsa-miR-876-5p | 0,097493783 | 0,257826146 | 0,060341 | 0,139 | 0,105 |
| hsa-miR-3135 | 0,151547966 | 0,143628772 | 0,12079372 | 0,139 | 0,016 |
| hsa-miR-1297 | 0,160092658 | 0,07766864 | 0,18025375 | 0,139 | 0,054 |
| hsa-miR-4694-3p | 0,241126542 | 0,15037586 | 0,02870465 | 0,140 | 0,107 |
| hsa-miR-500a | 0,167319962 | 0,17628108 | 0,07682868 | 0,140 | 0,055 |
| hsa-miR-4281 | 0,160436417 | 0,247245968 | 0,01348759 | 0,140 | 0,118 |
| hsa-miR-1277 | 0,181718343 | 0,080274802 | 0,16054022 | 0,141 | 0,054 |
| hsa-miR-4733-5p | 0,191094711 | 0,162050324 | 0,07026041 | 0,141 | 0,063 |
| hsa-miR-3115 | 0,213976534 | 0,207123734 | 0,00242262 | 0,141 | 0,120 |
| hsa-miR-616 | 0,203296665 | 0,23673831 | -0,01387824 | 0,142 | 0,136 |
| hsa-miR-4772-3p | 0,173352697 | 0,170135827 | 0,08271823 | 0,142 | 0,051 |
| hsa-miR-4764-3p | 0,178128641 | 0,224893389 | 0,02484924 | 0,143 | 0,105 |
| hsa-miR-4797-3p | 0,206676041 | 0,16058331 | 0,06114953 | 0,143 | 0,074 |
| hsa-miR-718 | 0,233768689 | 0,153290298 | 0,04243363 | 0,143 | 0,096 |
| hsa-miR-2355-5p | 0,189705751 | 1,17 | 0,09711789 | 0,486 | 0,595 |
| hsa-miR-543 | 0,006874756 | 0,23664847 | 0,18708522 | 0,144 | 0,121 |
| hsa-miR-3677-5p | 0,106878107 | 0,195332731 | 0,13027576 | 0,144 | 0,046 |
| hsa-miR-517a | 0,168224774 | 0,142009479 | 0,12562488 | 0,145 | 0,021 |
| hsa-miR-101 | -0,040900864 | 0,310616802 | 0,16709896 | 0,146 | 0,177 |
| hsa-miR-3155b | 0,192917882 | 0,13590686 | 0,10846299 | 0,146 | 0,043 |
| hsa-miR-4744 | 0,192049924 | 1,11 | 0,10091882 | 0,468 | 0,558 |
| hsa-miR-1915* | 0,138126416 | 0,601363288 | 0,15607227 | 0,299 | 0,262 |
| hsa-miR-320d | 0,204139855 | 0,049427852 | 0,18822391 | 0,147 | 0,085 |
| hsa-miR-4636 | 0,219069853 | 0,136676642 | 0,08666197 | 0,147 | 0,067 |
| hsa-miR-4499 | 0,173225025 | 0,203415273 | 0,06939975 | 0,149 | 0,070 |
| hsa-miR-4747-3p | 0,151646989 | 0,206894996 | 0,08886024 | 0,149 | 0,059 |
| hsa-miR-4659b-3p | 0,098792987 | 0,191740527 | 0,15938731 | 0,150 | 0,047 |
| hsa-miR-1207-5p | 0,107675233 | 0,002569546 | 0,34028893 | 0,150 | 0,173 |
| hsa-miR-3686 | 0,236018846 | 0,098999013 | 0,11744426 | 0,151 | 0,074 |
| hsa-miR-26a | 0,119444317 | 0,117922629 | 0,21515031 | 0,151 | 0,056 |
| hsa-miR-4781-5p | 0,279859558 | 0,123980667 | 0,04993993 | 0,151 | 0,117 |
| hsa-miR-548c-3p | 0,196843481 | 1,12 | 0,10620999 | 0,474 | 0,561 |
| hsa-miR-514 | 0,150152586 | 0,188781324 | 0,11973857 | 0,153 | 0,035 |
| hsa-miR-520e | 0,285851255 | 0,163792171 | 0,00928606 | 0,153 | 0,139 |
| hsa-miR-562 | 0,19879412 | 1,04 | 0,10789113 | 0,449 | 0,514 |
| hsa-miR-1295 | 0,198449107 | 1,13 | 0,11241053 | 0,480 | 0,564 |
| hsa-miR-377 | 0,169068238 | 0,202883693 | 0,09497311 | 0,156 | 0,055 |
| hsa-miR-563 | 0,20565913 | 0,177669439 | 0,08389644 | 0,156 | 0,064 |
| hsa-miR-486-3p | 0,055220209 | 0,242452266 | 0,1709908 | 0,156 | 0,094 |
| hsa-miR-4271 | 0,235389945 | 0,155110655 | 0,07854696 | 0,156 | 0,078 |
| hsa-miR-3676 | 0,08608493 | 0,162878197 | 0,22128224 | 0,157 | 0,068 |
| hsa-miR-371b-3p | 0,169915418 | 0,223385963 | 0,0774728 | 0,157 | 0,074 |
| hsa-miR-588 | 0,22495344 | 0,18230117 | 0,06521654 | 0,157 | 0,083 |
| hsa-miR-4526 | 0,17927042 | 0,175049194 | 0,1183842 | 0,158 | 0,034 |
| hsa-miR-34c-3p | 0,219915521 | 0,22444947 | 0,03256748 | 0,159 | 0,109 |
| hsa-miR-320b | 0,077606792 | 0,241505221 | 0,15928263 | 0,159 | 0,082 |
| hsa-miR-338-3p | 0,100204457 | 0,289613445 | 0,09188742 | 0,161 | 0,112 |
| hsa-miR-544 | 0,126577561 | 0,171616783 | 0,18730257 | 0,162 | 0,032 |
| hsa-miR-550a* | 0,124291451 | 0,199687147 |  | 0,162 | 0,053 |
| hsa-miR-518b | 0,220764662 | 0,154675883 | 0,11150178 | 0,162 | 0,055 |
| hsa-miR-3922-3p | 0,192108582 | 0,236892856 | 0,06619625 | 0,165 | 0,089 |
| hsa-miR-4793-3p | 0,282464352 | 0,119532148 | 0,09408587 | 0,165 | 0,102 |
| hsa-miR-4798-3p | 0,193892368 | 1,09 | 0,13863208 | 0,474 | 0,534 |
| hsa-miR-519b-3p | 0,242585569 | 0,165889784 | 0,09101834 | 0,166 | 0,076 |
| hsa-miR-548ag | 0,256069698 | 0,04676619 | 0,19689931 | 0,167 | 0,108 |
| hsa-miR-3124-3p | 0,101324377 |  | 0,23194491 | 0,167 | 0,092 |
| hsa-miR-551b | 0,056176855 | 0,3608869 | 0,08285552 | 0,167 | 0,169 |
| hsa-miR-200c* | 0,236822429 | 0,111013819 | 0,15276072 | 0,167 | 0,064 |
| hsa-miR-4483 | 0,156258312 | 0,275299752 | 0,07336521 | 0,168 | 0,102 |
| hsa-miR-106b | 0,101819377 | 0,182431994 | 0,22182888 | 0,169 | 0,061 |
| hsa-miR-4783-5p | 0,265191344 | 0,176628833 | 0,06564928 | 0,169 | 0,100 |
| hsa-miR-496 | 0,01502349 | 0,224304502 | 0,26887348 | 0,169 | 0,136 |
| hsa-miR-3129-5p | 0,189183645 | 0,061207318 | 0,25878075 | 0,170 | 0,100 |
| hsa-miR-4254 | 0,239684555 | 0,150133088 | 0,11991367 | 0,170 | 0,062 |
| hsa-miR-2054 | 0,061256569 | 0,069157957 | 0,37964632 | 0,170 | 0,182 |
| hsa-miR-188-3p | 0,071235178 | 0,231506547 | 0,21048283 | 0,171 | 0,087 |
| hsa-miR-3942-5p | 0,259194307 | 0,158052776 | 0,09828614 | 0,172 | 0,081 |
| hsa-miR-548s | 0,227390918 | 0,210699851 | 0,0792375 | 0,172 | 0,081 |
| hsa-miR-515-3p | 0,20076213 | 0,21898873 | 0,10671982 | 0,175 | 0,060 |
| hsa-miR-4256 | 0,289832753 | 0,20823835 | 0,03091839 | 0,176 | 0,132 |
| hsa-miR-4640-3p | 0,095157613 |  | 0,26026542 | 0,178 | 0,117 |
| hsa-miR-4801 | 0,133199376 | 0,305595929 | 0,09681282 | 0,179 | 0,112 |
| hsa-miR-200c | 0,121699666 | 0,233165717 | 0,18074611 | 0,179 | 0,056 |
| hsa-miR-448 | 0,051426966 | 0,245852416 | 0,24266573 | 0,180 | 0,111 |
| hsa-miR-4478 | 0,212102802 | 1,07 | 0,14876922 | 0,477 | 0,515 |
| hsa-miR-4728-3p | -0,03678556 | 0,186832905 | 0,39306722 | 0,181 | 0,215 |
| hsa-miR-4538 | 0,252884923 | 0,122498417 | 0,16882848 | 0,181 | 0,066 |
| hsa-miR-3183 | 0,311050815 | 0,035525218 | 0,19856313 | 0,182 | 0,139 |
| hsa-miR-491-3p | 0,06421994 | 0,279526486 | 0,20179895 | 0,182 | 0,109 |
| hsa-miR-4496 | 0,283369861 | 0,139300307 | 0,12345331 | 0,182 | 0,088 |
| hsa-miR-4491 | 0,236825591 | 1,05 | 0,12820138 | 0,472 | 0,504 |
| hsa-miR-1257 | 0,245629049 | 0,207280189 | 0,09725247 | 0,183 | 0,077 |
| hsa-miR-4711-3p | 0,186031004 | 0,26126334 | 0,10363915 | 0,184 | 0,079 |
| hsa-miR-374c | 0,223448032 | 0,220173205 | 0,10748396 | 0,184 | 0,066 |
| hsa-miR-4330 | 0,057473076 | 0,371224036 | 0,12400245 | 0,184 | 0,165 |
| hsa-miR-3187-3p | 0,262015999 | 0,018718121 | 0,27262466 | 0,184 | 0,144 |
| hsa-miR-4791 | 0,183434591 | 0,280368753 | 0,09278744 | 0,186 | 0,094 |
| hsa-miR-3158-5p | 0,175488094 | 0,312480539 | 0,07011387 | 0,186 | 0,122 |
| hsa-miR-30d* | 0,255125134 | -0,108895615 | 0,4121699 | 0,186 | 0,267 |
| hsa-miR-4715-5p | 0,076888212 | 0,287150422 | 0,19585323 | 0,187 | 0,105 |
| hsa-miR-607 | 0,224610945 | 1,1 | 0,15160292 | 0,492 | 0,528 |
| hsa-miR-550a | 0,138068169 |  | 0,23834285 | 0,188 | 0,071 |
| hsa-miR-4655-3p | 0,17673647 | 0,183732055 | 0,2051031 | 0,189 | 0,015 |
| hsa-miR-1268b | 0,210311204 | 0,096514533 | 0,26012829 | 0,189 | 0,084 |
| hsa-miR-590-5p | 0,13047999 | 0,316709599 | 0,12785675 | 0,192 | 0,108 |
| hsa-miR-148a | 0,127513635 | 0,228165902 | 0,22397759 | 0,193 | 0,057 |
| hsa-miR-593 | 0,288357994 | 0,174827813 | 0,12286742 | 0,195 | 0,085 |
| hsa-miR-505* | 0,099276776 | 0,458634709 | 0,03038168 | 0,196 | 0,230 |
| hsa-miR-4419b | 0,156468399 | 0,213848153 | 0,2197361 | 0,197 | 0,035 |
| hsa-miR-4535 | 0,256721366 | 1,13 | 0,13809 | 0,508 | 0,542 |
| hsa-miR-3657 | 0,214148455 | 0,31969637 | 0,06105309 | 0,198 | 0,130 |
| hsa-miR-3615 | 0,202108049 | 0,322888437 | 0,06993996 | 0,198 | 0,127 |
| hsa-miR-302b | 0,239190139 | 0,240285129 | 0,11565466 | 0,198 | 0,072 |
| hsa-miR-296-3p | 0,031087082 | 0,22049456 | 0,34579944 | 0,199 | 0,158 |
| hsa-miR-3124-5p | 0,313597845 | 0,245774761 | 0,04254746 | 0,201 | 0,141 |
| hsa-miR-4718 | 0,248891028 | 0,267226096 | 0,09352333 | 0,203 | 0,095 |
| hsa-miR-1538 | 0,281144973 |  | 0,12662506 | 0,204 | 0,109 |
| hsa-miR-4322 | 0,266299346 | 0,210270486 | 0,13811557 | 0,205 | 0,064 |
| hsa-miR-302d | 0,140604264 | 0,277706926 | 0,20143173 | 0,207 | 0,069 |
| hsa-miR-4457 | 0,107840996 | 0,281491059 | 0,23201805 | 0,207 | 0,089 |
| hsa-miR-520d-3p | 0,310364368 | 0,212679966 | 0,11191642 | 0,212 | 0,099 |
| hsa-miR-4655-5p | 0,262837135 | 0,180104612 | 0,19264724 | 0,212 | 0,045 |
| hsa-miR-3678-5p | 0,277234549 | 0,213435788 | 0,14850179 | 0,213 | 0,064 |
| hsa-miR-4768-5p | -0,056656513 | 0,181261409 | 0,51537498 | 0,213 | 0,287 |
| hsa-miR-4690-3p | 1,35863996 | 0,086697118 | 0,34014373 | 0,595 | 0,673 |
| hsa-miR-569 | 0,236620197 | 0,155570201 | 0,24810744 | 0,213 | 0,050 |
| hsa-miR-450a | 0,137217787 | 0,181475876 | 0,32163356 | 0,213 | 0,096 |
| hsa-miR-20a* | 0,223905148 | 1,51250222 | 0,20575487 | 0,647 | 0,749 |
| hsa-miR-367 | 0,140171326 | 0,262841528 | 0,24339196 | 0,215 | 0,066 |
| hsa-miR-454 | 0,357212333 | 1,13 | 0,08025629 | 0,522 | 0,544 |
| hsa-miR-1291 | 0,32331578 | 0,167989029 | 0,16695138 | 0,219 | 0,090 |
| hsa-miR-4633-3p | 0,163146938 | 0,311270994 | 0,18739896 | 0,221 | 0,079 |
| hsa-miR-937 | 0,107900796 | 0,341582028 | 0,21438819 | 0,221 | 0,117 |
| hsa-miR-4469 | 0,218435227 | 0,276587424 | 0,16947991 | 0,222 | 0,054 |
| hsa-miR-4785 | 0,24663943 | 0,080755769 | 0,33843726 | 0,222 | 0,131 |
| hsa-miR-4665-3p | -0,002216071 | 0,249609583 | 0,42970663 | 0,226 | 0,217 |
| hsa-miR-922 | 0,390694081 | 0,147290544 | 0,14422049 | 0,227 | 0,141 |
| hsa-miR-520a-3p | 0,193460972 | 0,131208437 | 0,36130045 | 0,229 | 0,119 |
| hsa-miR-497* | 0,159714937 | 0,299915816 |  | 0,230 | 0,099 |
| hsa-miR-4517 | 0,238908536 | 0,287326979 | 0,18100891 | 0,236 | 0,053 |
| hsa-miR-187* | 0,332291144 | 1,122477753 | 0,14091519 | 0,532 | 0,520 |
| hsa-miR-4661-3p | -0,105791528 | 0,243022075 | 0,57513345 | 0,237 | 0,340 |
| hsa-miR-217 | 0,301261521 | 0,174028841 | 0,24924941 | 0,242 | 0,064 |
| hsa-miR-3909 | 0,229886564 | 0,295710626 | 0,18849482 | 0,238 | 0,054 |
| hsa-miR-216a | 0,1915781 | 0,190334238 | 0,33243383 | 0,238 | 0,082 |
| hsa-miR-4453 | 0,285500033 | 1,18 | 0,19922454 | 0,555 | 0,543 |
| hsa-miR-3935 | 0,028201819 | 0,243207703 | 0,45604227 | 0,242 | 0,214 |
| hsa-miR-3679-3p | 0,283544261 | 0,295475202 | 0,15246158 | 0,244 | 0,079 |
| hsa-miR-1272 | 0,286738656 | 0,204023936 | 0,2538668 | 0,248 | 0,042 |
| hsa-miR-4477b | 0,327033441 | 0,245963361 | 0,17749318 | 0,250 | 0,075 |
| hsa-miR-302c | 0,289826623 | 0,278703878 | 0,21745914 | 0,262 | 0,039 |
| hsa-miR-99b | 0,158049044 | 0,280182012 | 0,34860181 | 0,262 | 0,097 |
| hsa-miR-4672 | 0,322301149 | 0,195372155 | 0,28181934 | 0,266 | 0,065 |
| hsa-miR-654-3p | 0,223723369 | 0,339281698 | 0,25953393 | 0,274 | 0,059 |
| hsa-miR-638 | 0,245332982 | 0,415040386 | 0,16656353 | 0,276 | 0,127 |
| hsa-miR-520c-3p | 0,502237224 | 0,257388252 | 0,08105306 | 0,280 | 0,212 |
| hsa-miR-4325 | 0,360758367 | 0,307808893 | 0,17693778 | 0,282 | 0,095 |
| hsa-miR-363 | 0,208447985 | 0,411722884 | 0,23839004 | 0,286 | 0,110 |
| hsa-miR-4454 | 0,316582422 | 0,21939658 | 0,32577523 | 0,287 | 0,059 |
| hsa-miR-23b* | 0,219985871 | 0,373044034 |  | 0,297 | 0,108 |
| hsa-miR-3689a-3p | 0,272354469 | 0,424371606 | 0,21608647 | 0,304 | 0,108 |
| hsa-miR-621 | 0,239319489 | 0,303751263 | 0,39566595 | 0,313 | 0,079 |
| hsa-miR-3677-3p | 0,356139868 | 0,353571941 | 0,23007375 | 0,313 | 0,072 |
| hsa-miR-4451 | 0,303860779 | 0,377157494 | 0,27160177 | 0,318 | 0,054 |
| hsa-miR-4436b-3p | 0,34509819 | 0,362277053 | 0,25118147 | 0,320 | 0,060 |
| hsa-miR-99a | 0,305688377 | 0,281056443 | 0,37362459 | 0,320 | 0,048 |
| hsa-miR-4725-3p | 0,230114606 | 0,510964865 | 0,21931896 | 0,320 | 0,165 |
| hsa-miR-4726-3p | 0,408656599 | 0,327274583 | 0,23233867 | 0,323 | 0,088 |
| hsa-miR-542-5p | 0,050088825 | 0,34031127 | 0,60267506 | 0,331 | 0,276 |
| hsa-miR-542-3p | 0,281418721 | 0,575439575 | 0,17993237 | 0,346 | 0,205 |
| hsa-miR-3659 | 0,235226566 | 0,350480532 | 0,45595983 | 0,347 | 0,110 |
| hsa-miR-675* | 0,270115054 | 0,458674096 | 0,32182983 | 0,350 | 0,097 |
| hsa-miR-1289 | 0,340985685 | 0,298043781 | 0,42364293 | 0,354 | 0,064 |
| hsa-miR-3178 | 0,331065095 | 0,439924936 | 0,29828344 | 0,356 | 0,074 |
| hsa-miR-3663-5p | 0,314705388 | 0,329617499 | 0,42967072 | 0,358 | 0,063 |
| hsa-miR-602 | 0,21428694 | 0,345258287 | 0,53634638 | 0,365 | 0,162 |
| hsa-miR-3682-3p | 0,354309166 | 0,469327582 | 0,31478327 | 0,379 | 0,080 |
| hsa-miR-371-3p | 0,429496299 | 0,420403665 | 0,30466203 | 0,385 | 0,070 |
| hsa-miR-1256 | 0,209481846 | 0,416140071 | 0,55906942 | 0,395 | 0,176 |
| hsa-miR-3117-5p | 0,409306032 | 0,455036263 | 0,40629953 | 0,424 | 0,027 |
| hsa-miR-4777-5p | 0,350097183 | 0,59524912 | 0,40226809 | 0,449 | 0,129 |
| hsa-miR-548m | 0,467019562 | 0,462878492 | 0,41867528 | 0,450 | 0,027 |
| hsa-miR-1247 | 0,413524478 | 0,547514244 | 0,41237405 | 0,458 | 0,078 |
| hsa-miR-516a-5p | 0,387075855 | 0,493433847 | 0,53794283 | 0,473 | 0,078 |
| hsa-miR-3610 | 0,457713237 | 0,476989479 | 0,4738458 | 0,470 | 0,010 |
| hsa-miR-320a | 0,390092368 | 0,525338849 | 0,53469144 | 0,483 | 0,081 |
| hsa-miR-4708-3p | 0,052484612 | 0,47569957 | 0,55268495 | 0,360 | 0,269 |
| hsa-miR-887 | 0,6063132 | 0,568045238 | 0,43553526 | 0,537 | 0,090 |
| hsa-miR-3939 | 0,749241214 | 0,647298641 | 0,46227392 | 0,620 | 0,145 |
| hsa-miR-3195 | 0,948004595 | 1,167086561 | 0,35026784 | 0,822 | 0,423 |
| hsa-miR-4632 | 0,785102007 | 0,876330968 | 0,91189154 | 0,858 | 0,065 |
| hsa-miR-4634 | 0,971813136 | 0,853008443 | 0,77921106 | 0,868 | 0,097 |
| hsa-miR-941 | 1,026029697 | 1,027483235 | 0,97263556 | 1,009 | 0,031 |
| hsa-miR-4695-3p | 1,253645866 | 1,199085702 | 1,41546374 | 1,289 | 0,113 |
